# Starvation Affects the Muscular Morphology, Antioxidant Enzyme Activity, Expression of Lipid Metabolism-Related Genes, and Transcriptomic Profile of Javelin Goby (*Synechogobius hasta*)

**DOI:** 10.1155/2022/7057571

**Published:** 2022-12-30

**Authors:** Xiangning Chen, Yili Xu, Xiangyu Cui, Siying Zhang, Xiangqi Zhong, Juntao Ke, Yuze Wu, Zhiyu Liu, Chaoqing Wei, Zhujin Ding, Jianhe Xu, Hanliang Cheng

**Affiliations:** ^1^Jiangsu Key Laboratory of Marine Biotechnology, Co-Innovation Center of Jiangsu Marine Bio-Industry Technology, Jiangsu Ocean University, Lianyungang 222005, China; ^2^Key Laboratory of Cultivation and High-Value Utilization of Marine Organisms, Fisheries Research Institute of Fujian, Xiamen 361000, China; ^3^Jiangsu Key Laboratory of Marine Bioresources and Environment, Jiangsu Ocean University, Lianyungang 222005, China

## Abstract

Fish in natural and cultivated environments can be challenged by starvation. However, inducing starvation in a controlled manner cannot only reduce feed consumption but also reduces aquatic eutrophication and even improves farmed fish quality. This study investigated the effects of starvation on the muscular function, morphology, and regulatory signaling in javelin goby (*Synechogobius hasta*) by evaluating the biochemical, histological, antioxidant, and transcriptional changes in the musculature of *S. hasta* subjected to 3, 7, and 14 days fasting. The muscle glycogen and triglyceride levels in *S. hasta* were gradually reduced under starvation, reaching their lowest at the end of the trial (*P* < 0.05). The levels of glutathione and superoxide dismutase were significantly elevated after 3–7 days of starvation (*P* < 0.05), but later returned to the level of the control group. The muscle of starved *S. hasta* developed structural abnormalities in some areas after 7 days of food deprivation, and more vacuolation and more atrophic myofibers were observed in 14-day fasted fish. The transcript levels of stearoyl-CoA desaturase 1 (*scd1*), the key gene involved in the biosynthesis of monounsaturated fatty acids, were markedly lower in the groups starved for 7 or more days (*P* < 0.05). However, the relative expressions of genes associated with lipolysis were decreased in the fasting experiment (*P* < 0.05). Similar declines in the transcriptional response to starvation were found in muscle *fatp1* and *ppar γ* abundance (*P* < 0.05). Furthermore, the *de novo* transcriptome of muscle tissue from the control, 3-day and 14-day starved *S. hasta* generated 79,255 unigenes. The numbers of differentially expressed genes (DEGs) identified by pairwise comparisons among three groups were 3276, 7354, and 542, respectively. The enrichment analysis revealed that the DEGs were primarily involved in metabolism-related pathways, including ribosome, TCA pathway, and pyruvate metabolism. Moreover, the qRT-PCR results of 12 DEGs validated the expression trends observed in the RNA-seq data. Taken together, these findings demonstrated the specific phenotypical and molecular responses of muscular function and morphology in starved *S. hasta*, which may offer preliminary reference data for optimizing operational strategies incorporating fasting/refeeding cycles in aquaculture.

## 1. Introduction

When faced with starvation stress, regardless of whether it is associated with nutrient deficiencies or environmental cues, fish have evolved many modifications in their behavior, morphology, physiology, and biochemistry that allow them to rebound from the negative effects of starvation [1, 2]. In fact, short-term fasting/refeeding cycles have been reported to trigger compensatory growth mechanisms [1–3]. The immediate response to food deprivation in many fish species includes suppressed growth parameters, which is inevitably accompanied by structural and functional alterations in metabolically active tissues. The liver generally plays a dominant role in regulating the metabolic changes of endogenous reserves to maintain the energy homeostasis of starving fish by consuming energy reserves, primarily hepatic fat. Aside from the liver, muscular, gastrointestinal, and other tissues also undergo significant alterations in energetic metabolism during food shortages [2, 4, 5]. Moreover, starved fish have been reported to exhibit inconsistencies in the mobilization of energy stores, particularly lipid utilization [1–3, 6, 7], which partly depends on the differences in fish species, storage sites, starvation duration, and other factors.

Muscle tissues are the main component of the fish body, contributing over 50% to the body mass of fish [8], and participate in multiple essential physiological processes at the mechanical and metabolic levels. Similar to the liver, the muscles are also an important energy reservoir in fish and are both anatomically and functionally influenced by nutrient deficiencies [2, 6, 9, 10]. Energy mobilization in the muscle of fasted fish can compensate for the lack of food intake to meet energy demands. However, this can induce/aggravate oxidative stress in the fish muscle due to an excessive increase in reactive oxygen species (ROS) generated by the aerobic metabolism of energy stores during food deprivation, particularly lipid oxidation [11]. The activation of the antioxidant system in fish under acute food deprivation has been reported in several previous studies [2, 12, 13], thereby preventing oxidative damage to lipids, proteins, and other macromolecules [14–16]. However, limited energy reserves can be completely exhausted if fasting is prolonged [2, 6], which can ultimately disturb muscular function and lead to adverse health effects or even death in cases of severe starvation. The utilization of energy reserves in starved fish is known to be species- and tissue-specific [2], and previous studies have also confirmed that growth performance can be substantially enhanced by incorporating short-term feeding-fasting cycles into fish culture protocols. Therefore, it would be meaningful to evaluate the influence of food deprivation on the muscle physiology, histology, and transcription of a variety of fish species, as this would provide key insights into the regulatory mechanisms of muscle metabolism in starved fish.

The javelin goby (*Synechogobius hasta*), a carnivorous fish species belonging to the Gobiidae family, is an important marine fish in several countries on the west Pacific coast, including the coast of Lianyungang, Jiangsu, China. In addition to its ecological relevance, this fish species has several advantages that make it uniquely suitable for fish farming, including fast growth rates, high nutritional value, strong disease resistance, and excellent adaptability to various water environments [5, 17, 18]. Research on *S. hasta* has primarily focused on taxonomic classification, evolutionary biology, and ecotoxicology [19–21]. For example, recent studies have characterized the biochemical and physiological changes of hepatic lipid metabolism in *S. hasta* exposed to heavy metal [20, 22, 23]. However, few studies have evaluated the effects of different aquaculture conditions on the nonhepatic organs of *S. hasta*. Our previous study demonstrated that short-term fasting could enhance hepatic fatty acid transport and lipid hydrolysis, damage the intestinal structure and integrity, and attenuate the digestive function of *S. hasta* [5]. Nevertheless, little is known regarding the molecular mechanisms through which fasting affects the *S. hasta* muscle. Therefore, our study sought to assess the phenotypic variations in histological, biochemical, and transcriptional profiles associated with the lipid metabolism of *S*. *hasta* muscle tissues as well as the muscular transcriptomic characteristics in response to starvation. The data from these integrative approaches provide crucial insights into the responses of *S. hasta* muscle tissues to fasting and the molecular mechanisms involved in coping with nutrient deprivation. In turn, these insights provide a theoretical basis for improving the quality of *S. hasta* and other farm-raised fish species by implementing reasonable fasting/refeeding strategies.

## 2. Methods and Materials

### 2.1. Ethics Statement

All experiments involving fish were conducted in compliance with the Care and Use of Laboratory Animals guidelines of Jiangsu Ocean University (Protocol No.: 2020-37) and were approved by the Institutional Animal Care and Use Committee of Jiangsu Ocean University.

### 2.2. Fish Maintenance and Experimental Design

The adult *S. hasta* were obtained from a local fish farm (Lianyungang, Jiangsu, China) and transported to the experimental facilities of the Jiangsu Key Laboratory of Marine Biotechnology (Lianyungang, Jiangsu, China). All fish were reared in 240 L indoor tanks as previously described [5, 17, 22]. Briefly, the fish were maintained in artificial seawater under the following physicochemical conditions: water temperature, 20–23°C; pH, 7.5–8.0; salinity, 18–21 ppt; dissolved oxygen, ≥6.0 mg/L; 12 h light/dark cycle. The fish were fed daily with minced shrimp scraps (5%–6% of fish body weight) at 8 : 30 and 17 : 00. The residual shrimp scraps were removed 30 min after the completion of feeding, and half of the seawater was renewed daily. Water quality was monitored regularly throughout the entire trial. *S. hasta* were kept in these experimental conditions for a 2-week acclimation period.

In the starvation experiment, similarly sized sixty *S. hasta* (36 ± 1.3 cm, 190 ± 2.3 g) were randomly allocated to four experimental groups (one control group and three starvation groups, five fish per group with each treatment in triplicate). More details on our experimental procedures are provided in our previous study [17]. Briefly, the control fish were fed with minced shrimp scraps twice per day until the entire experiment had concluded. The fish in starvation groups I, II, and III were starved for 3, 7, and 14 days, respectively. All fish were maintained in the aforementioned acclimation conditions and were euthanatized with 100 mg/L MS-222 at the end of the fasting trial.

### 2.3. Biochemical and Antioxidant Indices

Muscle tissues were isolated on ice, cleaned in ice-cold sterilized PBS, and accurately weighed. The muscle samples were homogenized in ice-cold sterilized PBS (weight: volume ratio = 1 : 4 or 1 : 9 g/mL) using an OSE-Y50 tissue grinder (Tiangen Biotech Co., Ltd., Beijing, China) and centrifuged at 2500 rpm/min at 4°C for 10 min. The resulting supernatant was collected for the evaluation of antioxidative enzymes (catalase (CAT), glutathione peroxidise (GSH-Px), and superoxide dismutase (SOD)), glutathione (GSH), total antioxidative capacity (T-AOC), and malondialdehyde (MDA) using the appropriate commercial kits (Nanjing Jiancheng, Nanjing, China).

The contents of glycogen, total triglyceride (TG), and total protein (TP) in muscle were measured using the corresponding kits (Nanjing Jiancheng, Nanjing, China) according to the manufacturer's instructions. All measurements were performed using a Multiskan FC photometer (Thermo Scientific, Waltham, MA, USA).

### 2.4. Hematoxylin and Eosin (HE) Staining

The freshly dissected muscle tissues were fixed in 10% neutral buffered formaldehyde and incubated overnight at 4°C. After dehydrating the samples in an ethanol gradient and clearing in xylene, square slices of muscle tissues (2.0 × 2.0 × 0.3 cm) were embedded in paraffin and sectioned at 4–5 *μ*m. The slices were then dewaxed, rehydrated with distilled water, and incubated with HE solution at room temperature (RT) for 5 min. Finally, once the samples were dehydrated in an ethanol gradient, cleared with xylene, and sealed with neutral gum, the stained muscular sections were photographed under a BX63 microscope (Olympus, Tokyo, Japan). The fiber diameter was determined by evaluating 150 randomly selected fibers (30 fibers × 5 randomly selected visions in each section).

### 2.5. RNA Isolation, Library Construction, and Sequencing

Muscle tissues from control *S. hasta*, 3-day and 14-day starved *S. hasta*, were named as MC, MS, and ML, respectively. These samples were flushed with RNase-free water, immediately snap frozen in liquid nitrogen, and kept at −80°C for subsequent RNA-seq analyses. The total RNA of individual samples (9 samples in total, 3 groups × 3 replicates) was extracted following standard protocols. The quantity and purity of the isolated RNA were then assessed using a BioPhotometer D30 UV-VIS photometer (Eppendorf, Hamburg, Germany), and RNA integrity and quality were assessed using 1% agarose gel electrophoresis and an Agilent 2100 Bioanalyzer (Agilent Technologies, Santa Clara, CA, USA), respectively.

High-quality RNA samples (OD260/OD280 = 1.98–2.10, RIN = 7.8–9.9) were pooled for cDNA library construction and then sequenced by Novogene Co., Ltd (Beijing, China) on an Illumina Hiseq sequencer. Details on these procedures are provided in the Supplementary Method.

### 2.6. Quality Control, *De Novo* Assembly, and Unigene Annotation

The raw RNA-seq reads in FASTQ format were first filtered using in-house Perl scripts and further cleaned by removing reads with adaptors, ploy-N, and low-quality reads (base quality < 20).

The clean reads were then assembled using the Trinity software [24] with all of the default parameters except for min_kmer_cov, which was set to 2. Basic annotation of unigenes and transcripts was carried out using seven commonly-used databases. Functional identification was conducted using BLASTx with an *E* value threshold of 1.0*E* − 5.

### 2.7. Screening and Analysis of Differentially Expressed Genes (DEGs)

The expression levels of clean reads mapped to unigenes were estimated using RSEM [25] and represented as FPKM values (the number of fragments per kilobase of transcript sequence per million base pairs sequenced). Differential expression analysis among different samples was conducted using the “DESeq” R package (1.10.1). *P* values were adjusted using the Benjamini and Hochberg method to control for false discovery rates (FDR). Genes were considered DEGs at an adjusted *P* < 0.05 and |log2(fold change)| ≥ 1.

GO enrichment analysis of the obtained DEGs was conducted using the GOseq R package [26], and KEGG pathway enrichment analyses were carried out using the KOBAS software [27]. Gene enrichment was considered significant at a corrected *P* < 0.05.

### 2.8. Quantitative Real-Time Reverse Transcription-PCR (qRT-PCR)

The mRNA expressions of lipid metabolism-associated genes and DEGs were measured by qRT-PCR using a StepOne Plus Real-Time PCR system (Applied Biosystems, Foster City, CA, USA). The cDNA synthesis and qRT-PCR assays were conducted according to previous reports with some modifications [5, 17, 28, 29]. Briefly, first-strand cDNA was synthesized from 1 *μ*g of the extracted total RNA sample using the PrimeScript™ RT Reagent Kit and gDNA Eraser (Takara, Otsu, Shiga, Japan), and then used as a template in the qRT-PCR reaction using the TB Green® Premix Ex Taq™ II (Takara). The amplification process was conducted in the following conditions: initial denaturation at 95°C for 30 s, followed by 40 cycles of 95°C for 5 s and 60°C for 30 s, and finally 95°C for 15 s, 60°C for 60 s, and 95°C for 15 s. The target gene expression was calculated as its abundance relative to the abundance of the internal reference gene following the relative standard curve method. Based on the amplification efficiency and dissociation curve of three housekeeping genes (*18* s, *β-actin*, and *gapdh*), *β-actin* was identified as the most stable internal standard in this study. Each reaction included serial dilutions of a DNA standard and a nontemplate control, and all samples were evaluated in triplicates. The gene-specific primers for qRT-PCR were synthesized by Sangon (Shanghai, China) and are summarized in Table 1.

### 2.9. Statistical Analysis

All the results were expressed as mean values ± standard deviation (SD) and assessed using GraphPad Prism 8.0 (GraphPad, La Jolla, CA, USA). If normality and equality of variance were satisfied, the data were analyzed via one-way analysis of variance (ANOVA) coupled with Tukey's test. Otherwise, Welch's ANOVA test was applied as a *post hoc* analysis to determine differences if the data did not satisfy variance homogeneity. Nonnormal data were compared using the Kruskal-Wallis test and Dunn's multiple comparisons. A *P* value < 0.05 was considered statistically significant.

## 3. Results

### 3.1. Biochemical and Antioxidant Activity in the Muscle of Starved *Synechogobius hasta*

**Figure 1:**
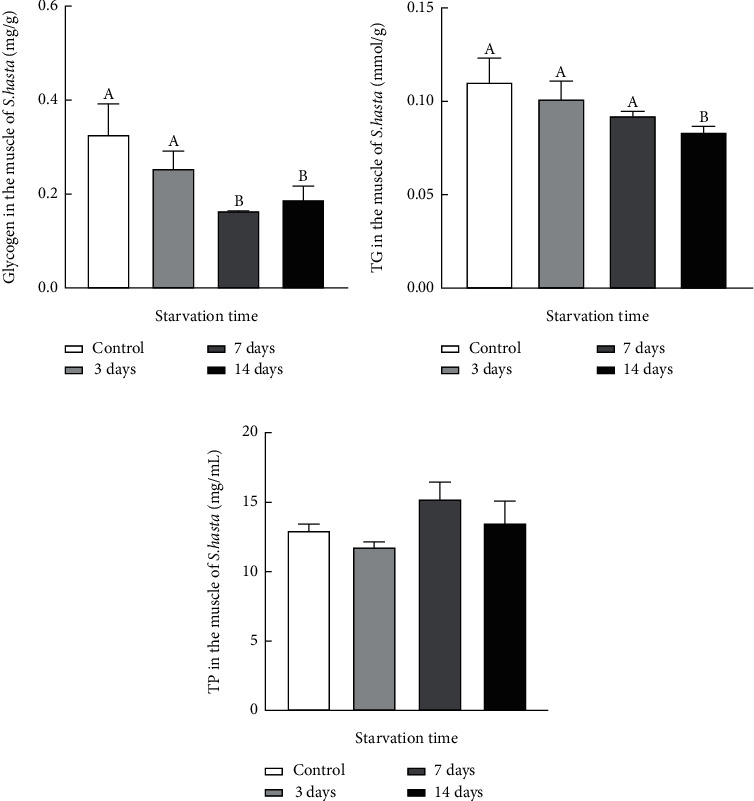
Effects of starvation on glycogen, triglyceride, and protein content in the muscle of *Synechogobius hasta*. Concentrations of glycogen (a), total triglyceride (b), and total protein (c) in the muscle of *S. hasta* subjected to different starvation periods. All values were represented as means ± SD (*n* = 5) and analyzed using one-way ANOVA. Different uppercase letters denote significant differences (*P* < 0.05).

No fatalities or gross abnormalities were observed in *S. hasta* throughout the course of the experiment. Both the average glycogen and TG content in the muscle showed a decrease with starvation time, reaching the lowest value at the end of the 14-day food deprivation period (*P* < 0.05; Figure 1). Moreover, no significant alterations in muscular TP were observed among any of the examined groups (*P* > 0.05).

**Figure 2:**
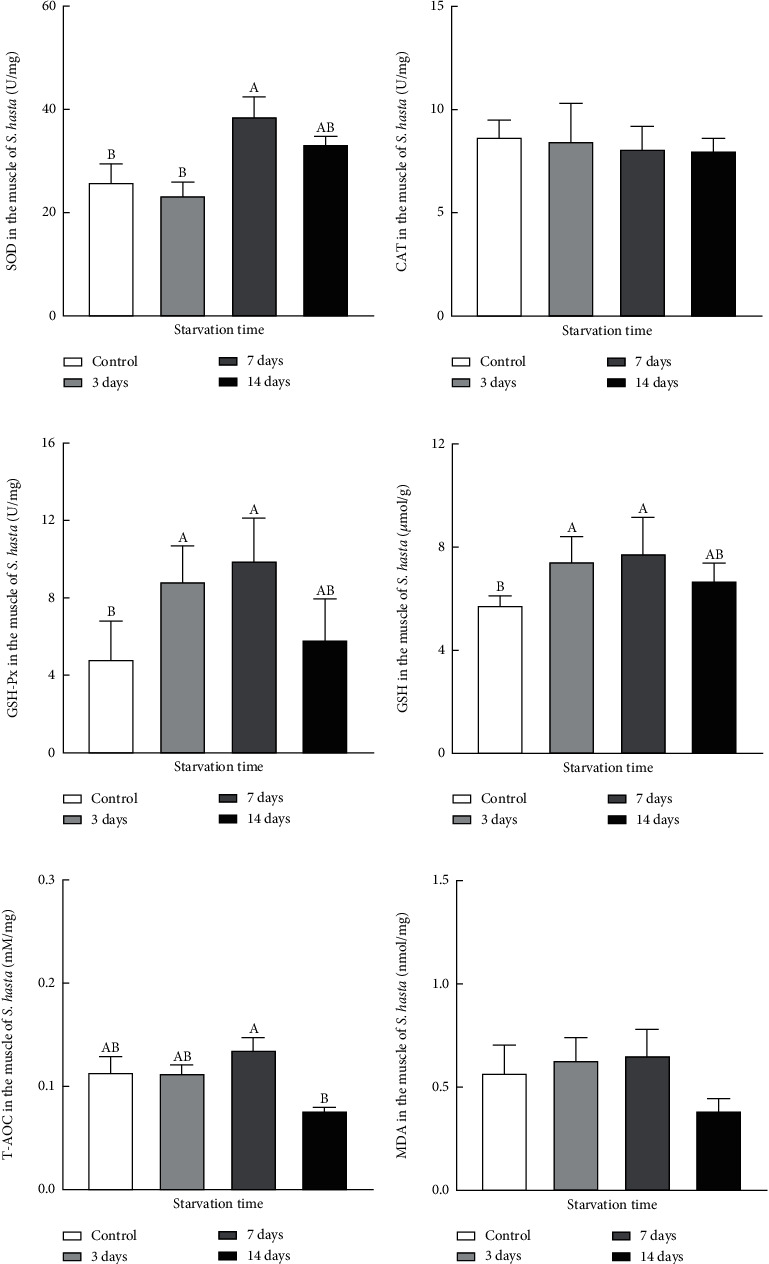
Effects of starvation on the muscular antioxidant capacity of *Synechogobius hasta.* Changes in SOD (a), CAT (b), GSH-Px (c), GSH (d), T-AOC (e), and MDA (f) in the muscle of *S. hasta* subjected to different starvation periods. Antioxidant index values were represented as means ± SD (*n* = 5) and evaluated using one-way ANOVA. Different uppercase letters denote significant differences (*P* < 0.05).

The muscle SOD activity was significantly elevated after food deprivation for 7 days (*P* < 0.05) and slightly decreased to the same level as the control when fasting was extended to 14 days (*P* > 0.05; Figure 2). GSH-Px and GSH in the muscle increased significantly from day 3 to day 7 of fasting (*P* < 0.05), and then gradually decreased at the end of the 14-day starvation period (*P* > 0.05; Figure 2). The muscle T-AOC in *S. hasta* tended to stay relatively stable for 0–7 days of food shortage (*P* > 0.05) but decreased significantly in fish starved for 14 days (*P* < 0.05) compared to the other groups (Figure 2). No significant changes in CAT or MDA were identified throughout the entire trial (*P* > 0.05; Figure 2).

### 3.2. Histological Structure of Starved *Synechogobius hasta* Muscle

**Figure 3:**
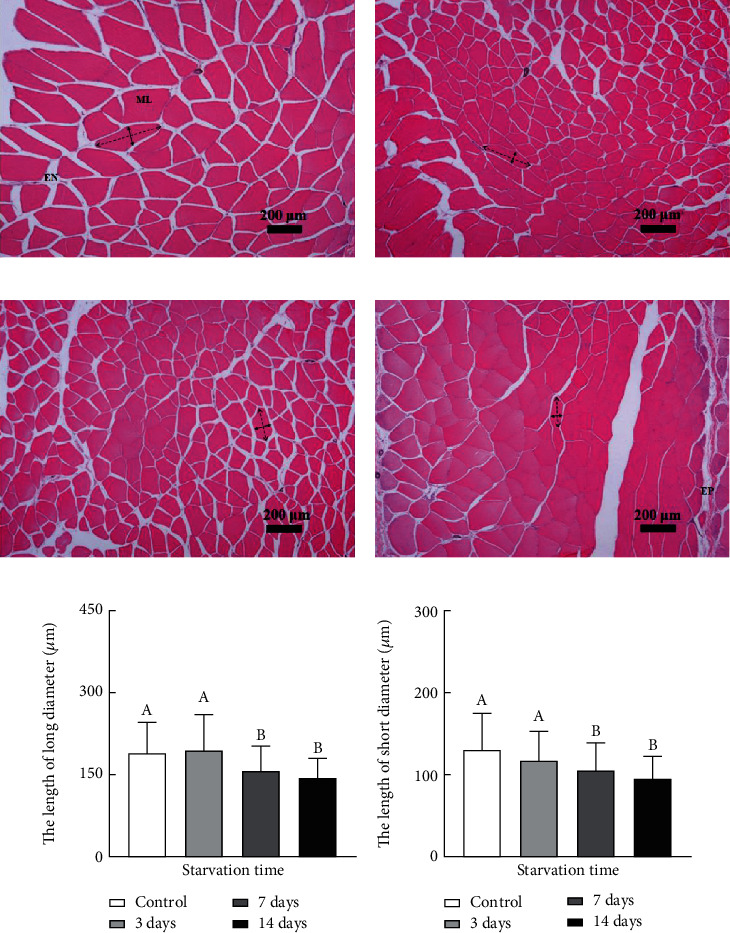
Effects of starvation on the muscle histological structure of *Synechogobius hasta.* Representative cross sections of muscle tissues of *S. hasta* fed as normal (a) and subjected to 3 (b), 7 (c), and 14 (d) days of starvation. The sections were stained with hematoxylin and eosin. MF: muscle fiber; EN: endomysium; EP: epimysium; dotted double arrow: long diameter (maximum length of the skeletal muscle parallel to the long axis of the skeletal muscle); solid double arrow: short diameter (maximum width of the skeletal muscle perpendicular to the long diameter). Scale bar: 200 *μ*m. Length of long (e) and short (f) diameter in the muscle of *S. hasta* subjected to different starvation periods. All values were represented as means ± SD (*n* = 150) and analyzed using the Kruskal-Wallis ANOVA with Dunn's test. Different uppercase letters denote significant differences (*P* < 0.05).

Under the optical microscope, the muscle fibers in the control fish appeared neatly arranged and evenly distributed, exhibiting a normal morphology (Figure 3(a)). The myofibers in the 3-day fasted group remained largely intact and normal shaped compared with the control group, with some showing enlarged intercellular gaps (Figure 3(b)). As the fasting period increased to 7 days, the muscle cells of *S. hasta* appeared to shrink and exhibited enlarged intercellular spaces (Figure 3(c)). The muscular cells from the 14-day starved *S. hasta* exhibited markedly irregular morphology, displaying blurred boundaries, more contracted cells, detached myofibers in some areas, and even inward migration of muscle nuclei (Figure 3(d)).

The mean diameter of short muscle fibers and long muscle fibers did not significantly change after 0-3 days of starvation (*P* > 0.05; Figure 3(f)) but decreased markedly from 7th days postfasting (*P* < 0.05; Figure 3(e)).

### 3.3. Lipid Metabolism-Related Gene Expression in the Muscle of Starved *Synechogobius hasta*

**Figure 4:**
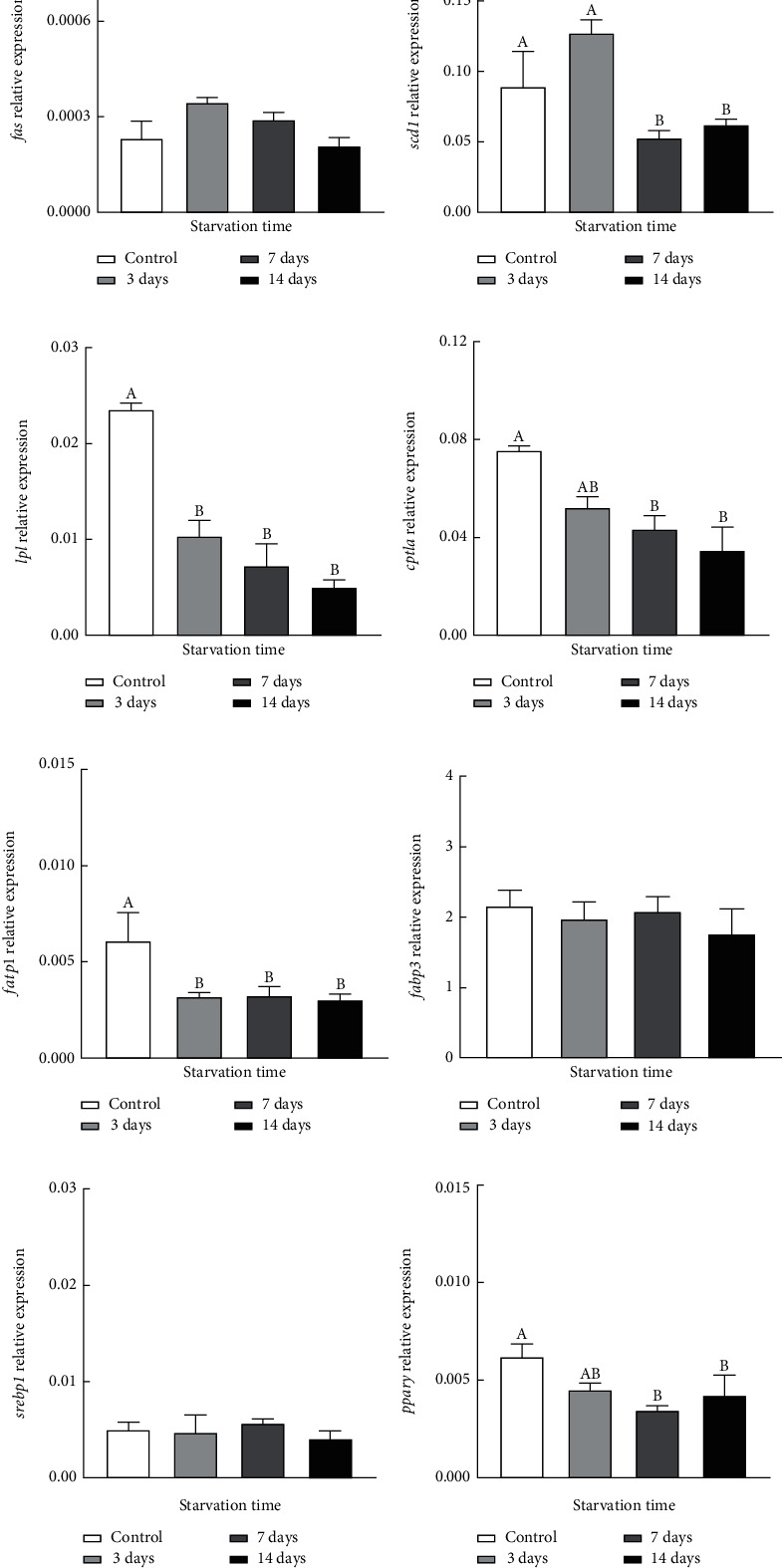
Effects of starvation on lipid metabolism-related gene expression in the muscle of *Synechogobius hasta*. Relative expression abundances of *fas* (a), *scd1* (b), *lpl* (c), *cpt1a* (d), *fatp1* (e), *fabp3* (f), *srebp1* (g), and *ppar γ* (h) in the muscle of starved *S. hasta*. The transcript level was measured by quantitative real-time PCR (qRT-PCR) and normalized to the abundance of a housekeeping gene (*β-*actin). All data were represented as means ± SD (*n* = 4 − 5), and differences between groups were determined via one-way ANOVA. Columns with different letters were deemed significantly different (*P* < 0.05).

No statistical differences in the expression levels of fatty acid synthase (*fas*), fatty acid binding protein 3 (*fabp3*), or sterol regulatory element binding protein 1 (*srebp1*) were observed in the muscle of *S. hasta* in any of the examined groups (*P* >0.05). The mRNA expression of stearoyl-CoA desaturase 1 (*scd1*) remained stable at the early stage of fasting but was obviously downregulated from the 7^th^ to 14^th^ day of starvation (*P* < 0.05; Figure 4). The transcript levels of lipoprotein lipase (*lpl*), carnitine palmitoyltransferase 1a (*cpt1a*), and fatty acid transport protein 1 (*fatp1*) exhibited a progressive decline with starvation and reached the lowest points by the end of the trial (*P* < 0.05; Figure 4). Furthermore, fasting caused a gradual decrease in peroxisome proliferator-activated receptor *γ* (*ppar γ*) expression in the muscle of *S. hasta* (*P* < 0.05; Figure 4).

### 3.4. Characteristics of RNA-Seq Data from Starved *Synechogobius hasta* Muscle

An average of 43,622,635, 43,602,655, and 44,420,961 clean reads were obtained from the muscle samples from the three groups (control, 3-day, and 14-day starved group, named as MC, MS, and ML, respectively) after technical filtration and assessment, respectively (Table 2, Table S1). These high-quality data had more than 96% Q20, more than 91% Q30, and a 0.03% error rate in each library (Table 2). All clean reads were assembled *de novo*, resulting in 79,255 unigenes (average length: 1,274 bp; N50: 2,333 bp) and 196,071 transcripts (average length: 1,852 bp; N50: 3,348 bp) (Table 3).

**Figure 5:**
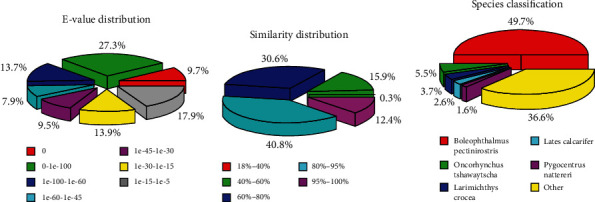
Similarity analysis of unigenes in *Synechogobius hasta* based on Nr database. Classification analysis of unigenes against the Nr database for *E* value distribution (a), similarity distribution (b), and species distribution (c). All unigenes from *S. hasta* were analyzed using the online BLASTx program with an *E* value ≤ 10^−5^.

Based on the information in Table 4, a total of 47,109 unigenes from *S. hasta* muscle were matched to at least one database, accounting for 59.43% of all unigenes (Figure S1). When the sequences were aligned to those in the Nr database using the BLASTx algorithm, nearly 37% of the sequences showed strong homology, with an *E* value below 1*E* − 100 (Figure 5(a)), whereas 53.2% of the unigenes shared over 80% similarity among the similarity distribution with the best hits (Figure 5(b)). Furthermore, 49.7% of the matched unigenes exhibited the highest similarity with *Boleophthalmus pectinirostris*, followed by *Oncorhynchus tshawytscha* (5.5%), *Larimichthys crocea* (3.7%), *Lates calcarifer* (2.8%), and *Pygocentrus nattereri* (1.6%) (Figure 5(c)).

### 3.5. DEGs Identification and Enrichment Analyses

**Figure 6:**
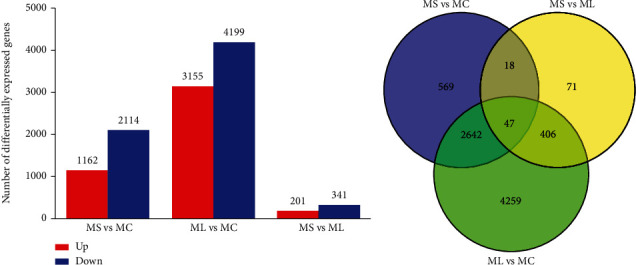
Differentially expressed genes (DEGs) profile of the muscle samples of *Synechogobius hasta* subjected to starvation. Bar graph (a) and Venn diagram (b) of DEGs between the different pairwise comparisons. MC: muscle in the control group (continuously fed for 14 days); MS: muscle in the starved group (starved for 3 days); ML: muscle in the starved group (starved for 14 days). Three replicates were used for each group in the transcriptome analysis. A subset of genes with *P* < 0.05 and |log2(fold change)| ≥ 1 is shown as the overlapped area in the Venn diagram.

At a threshold of |log2 (fold change)| ≥ 1 and *P* adjusted < 0.05, a total of 3,276 genes (1,162 upregulated and 2,114 downregulated) in the muscle were identified as DEGs between MS and MC (Figure 6(a), Table S2). By comparing ML versus MC, 3,155 genes were found to be upregulated, whereas 4,199 genes were downregulated (Figure 6(a), Table S2). Moreover, 542 DEGs (201 upregulated and 341 downregulated) were observed in MS compared with ML (Figure 6(a), Table S2). Additionally, 47 DEGs were coexpressed across the pairwise comparisons of all three groups (Figure 6(b)).

**Figure 7:**
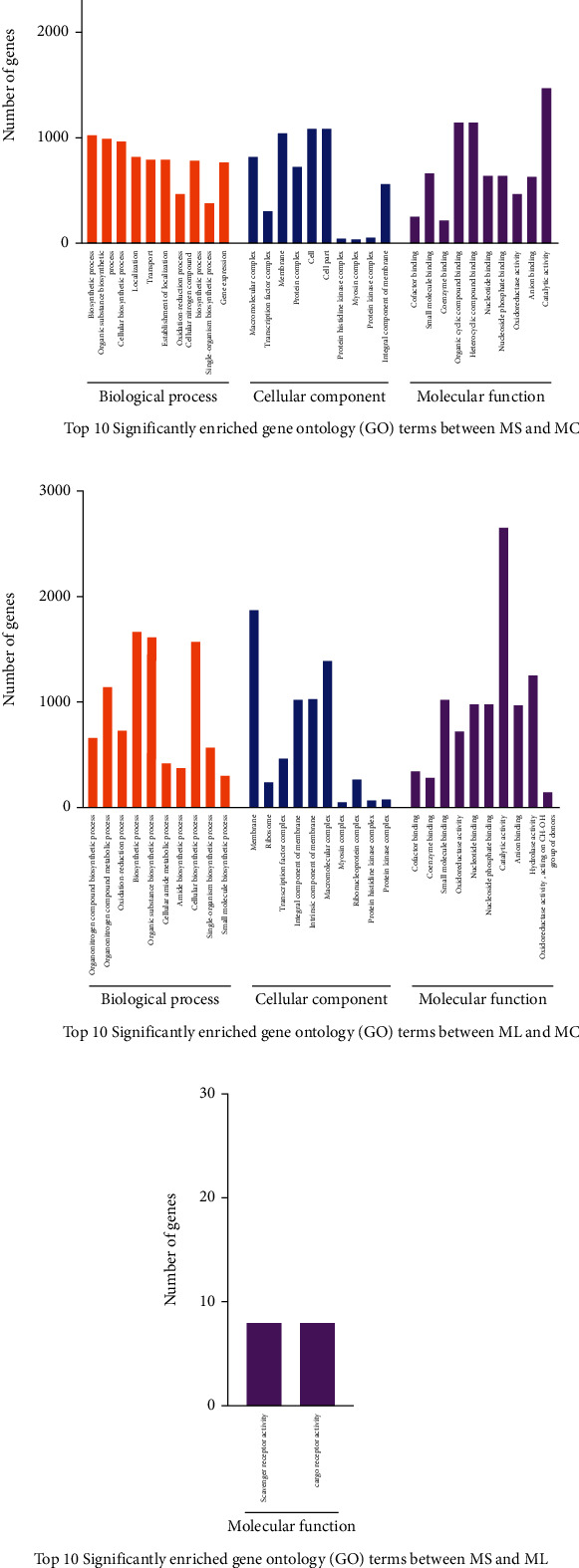
Significantly enriched gene ontology (GO) terms of the DEGs in *Synechogobius hasta* muscle. Top significantly enriched GO terms for DEGs in the comparison of MS *vs*. MC (a), ML *vs*. MC (b), and MS *vs*. ML (c). The horizontal axis denotes the number of DEGs with a significantly enriched GO term. The vertical axis represents the significantly enriched GO term. The GO terms are grouped into three categories: biological process (BP), cellular component (CC), and molecular function (MF). The size and color of the columns represent the number of DEGs with a significantly enriched GO term and the functional classification, respectively. When more than 10 enriched GO terms (corrected *P* ≤ 0.05) for DEGs were identified, only the top 10 significantly enriched GO terms were listed. MC: muscle in the control group (continuously fed for 14 days); MS: muscle in the starved group (starved for 3 days); ML: muscle in the starved group (starved for 14 days).

In the GO enrichment analysis of the screened sequences, 2,494 DEGs between MS and MC were significantly grouped into three ontologies, containing 406, 45, and 201 GO terms in the biological process (BP), cellular component (CC), and molecular function (MF) categories (Figure 7(a), Figure S2, Table S3). Similarly, 415 significantly enriched GO terms were observed when comparing ML *vs.* MC, with 62.41% belonging to BP, 5.3% to CC, and 32.29% to MF (Figure 7(b), Figure S2, Table S3). However, only two MF entries were significantly enriched between the two starved groups, namely, scavenger receptor activity (GO: 0005044) and cargo receptor activity (GO: 0038024) (Figure 7(c), Figure S2, Table S3).

**Figure 8:**
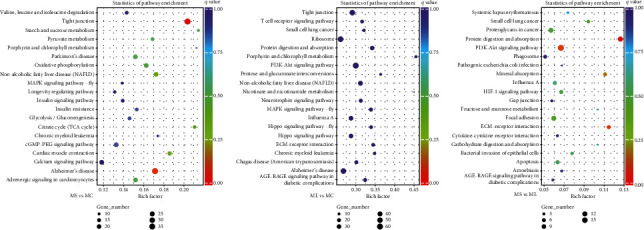
Kyoto encyclopedia of genes and genomes (KEGG) pathway enrichment analysis of DEGs in *Synechogobius hasta* muscle (Top 20). Representative top 20 pathways of DEGs identified in the comparison of MS *vs*. MC (a), ML *vs*. MC (b), and MS *vs*. ML (c). The horizontal axis denotes the ratio of DEGs with a specific pathway term relative to all DEGs. The vertical axis represents the annotated KEGG pathway term. The size and color of the bubbles represent the number of DEGs with a specific KEGG pathway and the enriched significance, respectively. MC: muscle in the control group (continuously fed for 14 days); MS: muscle in the starved group (starved for 3 days); ML: muscle in the starved group (starved for 14 days).

KEGG enrichment analysis elucidated 248 pathways that were enriched with the target DEGs between MS and MC, among which the “Tight junction” pathway was significantly enriched with 35 DEGs (adjusted *P* < 0.05) (Figure 8(a), Table S4). When contrasting ML and MC, there were 282 KEGG pathways identified in the muscular DEGs, but no significantly enriched pathway was detected (Figure 8(b)). However, downregulated DEGs for MS *vs.* MC and ML *vs.* MC were significantly enriched in 8 and 13 known pathways, many of which were metabolism associated (e.g., “Ribosome,” “Pyruvate metabolism,” and “TCA cycle”) (Figure S3, Table S4). Additionally, 14 upregulated and 73 downregulated DEGs were linked to 178 KEGG pathways in the comparison of MS *vs.* ML. Nevertheless, only “Protein digestion and absorption” was significantly enriched (adjusted *P* < 0.05) (Figure 8(c), Table S4).

### 3.6. Validation of DEGs by qRT-PCR

**Figure 9:**
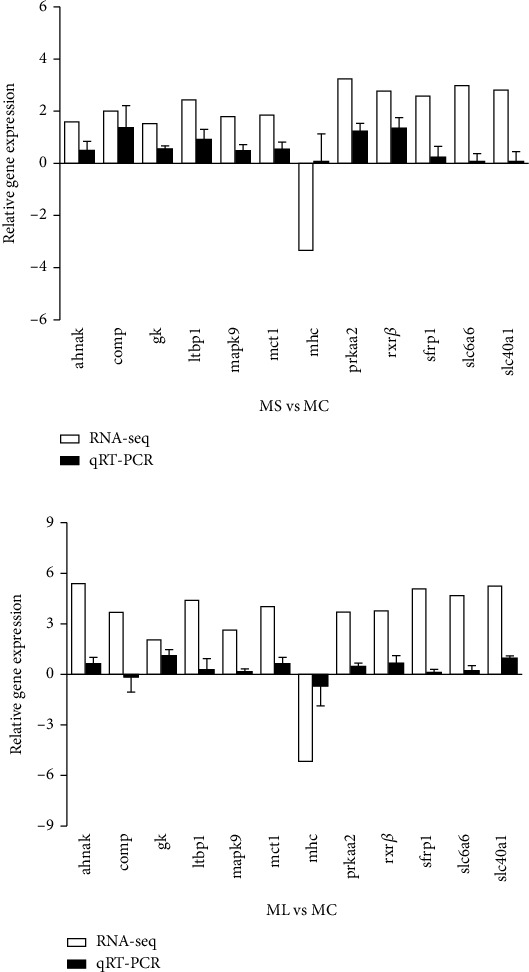
Validation of the expression of DEGs by qRT-PCR. Relative expression abundances of 12 DEGs validated by qRT-PCR in MS *vs*. MC (a) and ML *vs*. MC (b). The transcript level was measured by quantitative real-time PCR (qRT-PCR) and normalized to the abundance of a housekeeping gene (*β-*actin). The data are represented as means ± SD (*n* = 3). MC: muscle in the control group (continuously fed for 14 days); MS: muscle in the starved group (starved for 3 days); ML: muscle in the starved group (starved for 14 days).

The expression levels of twelve randomly chosen DEGs were evaluated via qRT-PCR to validate the transcriptomic data of *S. hasta* muscle. As illustrated in Figure 9, the relative expression patterns determined by qRT-PCR were largely consistent with those obtained from RNA-seq (Figure S4, Table S5).

## 4. Discussion

### 4.1. Effects of Starvation on Biochemical Parameters in the Muscle of *Synechogobius hasta*

Fish can survive in situations of hunger through adaptive alterations in metabolically active tissues [1–3, 30], including multiple biochemical and physiological properties associated with energetic homeostasis as well as behavioral patterns. Our study identified a decline in the muscle glycogen content of starved *S. hasta*, which was more pronounced on the 7^th^ and 14^th^ day of fasting (Figure 1), indicating that glycogen was mobilized by the muscle tissues of *S. hasta* from the middle stage of short-term fasting. These results were consistent with previous studies on several fish species subjected to starvation, in which the rapid utilization and significant decrease of glycogen were detected in the muscle tissues during food deprivation, especially during short-term food restriction [2, 6, 16, 31, 32]. The fasting-induced reduction in muscle TG was detected in 14-day starved *S. hasta*, thus confirming that triglycerides and other lipid metabolites are readily used as energy sources by many teleost species [2, 16, 32, 33], including *S. hasta* in the present study. However, no significant changes in muscle TP were observed throughout the entire fasting trial. Moreover, the muscle glycogen in *S. hasta* without feeding appeared to decrease faster and to a greater degree than that of muscular TG (Figure 1), suggesting that *S. hasta* starved for 14 days preferentially used glucose as an energy source, followed by fat.

### 4.2. Effects of Starvation on Antioxidant Capacity in the Muscle of *Synechogobius hasta*

Previous studies have suggested that food deprivation has a prooxidant effect on aquatic organisms [6, 34], as fasting induces the generation of reactive oxygen species (ROS) in some fish species [5, 11, 13, 35]. To date, research on starvation-induced oxidative stress in fish has mainly focused on the liver and gastrointestinal tract rather than muscle tissue despite the fact that muscle accounts for the overwhelming majority of the fish mass [8]. Moreover, muscle tissues play a crucial role in the metabolic homeostasis of fish [29, 36]. The significant elevation of muscle SOD in the 7-day fasted group appeared to neutralize starvation-induced intramuscular ROS to some extent. However, the SOD activity of the 14-day fasted group decreased to similar levels to those of the control and the 3-day starved *S. hasta* (Figure 2). In contrast, the mean activity of CAT was not notably altered (Figure 2). Our results appeared to differ from those of previous studies that reported a dramatic upregulation of the intestinal and hepatic SOD and CAT activities in other fish species under starvation [37–39]. In addition to species-specific variations, these discrepancies might be attributed to variations in the sensitivities of internal organs in response to starvation and other short-term nutritional challenges. Similar findings were reported in the muscle of European sea bass (*Dicentrarchus labrax*) subjected to long-term food deprivation [37]. Specifically, the muscle SOD activity decreased significantly after three weeks of fasting, whereas no alterations were identified in the muscle CAT activity under the same conditions [37].

GSH-Px and its substrate GSH were both markedly increased in *S. hasta* muscle upon starvation, and their levels remained high from day 3 to 7 postfasting. Other studies have also reported that the resistance of teleost fish to oxidative stress increases in response to acute food scarcity [2, 38–40]. Given the lack of exogenous supplementation of selenium or sulfur amino acids for GSH production under fasting conditions [2, 38, 41], the limited muscle GSH was insufficient to ensure a normal level of GSH-Px activity for H_2_O_2_ scavenging. In turn, this resulted in decreased H_2_O_2_ neutralization and excessive ROS accumulation. In our study, we also observed a substantial decline in GSH-Px and GSH contents in *S. hasta* muscle at the end of the fasting trial.

Hunger-induced oxidative stress/ROS generation can impair antioxidant capacity and other physiological processes in fish and other organisms via lipid peroxidation, which is manifested as an increase in MDA level in the metabolically active tissues of some starved teleost [12, 13, 38–40]. Unexpectedly, no obvious changes in muscle MDA levels were found in our study. In fact, the MDA levels in muscle appeared to decrease slightly albeit not significantly by the end of the starvation period. This discrepancy might be due to the physiological properties of muscular tissue. White muscle, which accounts for the majority of the fish body, needs less oxygen than aerobiotic tissues such as the liver and red muscle, which may contribute to its stronger resistance to short-term starvation-induced ROS, thereby requiring less antioxidant activity [38]. Similar findings were reported in Adriatic sturgeon (*Acipenser naccarii*) and rainbow trout (*Oncorhynchus mykiss*), in which unchanged lipid peroxidation and enhanced activities of antioxidant enzymes occurred in the white muscle within 70 days of fasting [2, 12]. In our study, T-AOC levels were also measured as an indicator of overall antioxidant status in *S. hasta* muscle, and our results were largely consistent with the alterations in enzymatic antioxidants and nonenzymatic antioxidants measured above. Concretely, T-AOC levels remained stable or tended to increase slightly in the early and middle stages of food deprivation, which was followed by a sharp decrease at the end of the fasting trial. This suggested that prolonged fasting (beyond 14 days) causes a sustained depletion of nutritional reserves, including various antioxidants, and eventually, hinders the overall metabolic function of *S. hasta*, as did the antioxidant capacity of *S. hasta* and other starved fish species in previous studies [38]. Therefore, it may be inferred that the white muscle in *S. hasta* could counteract ROS accumulation and oxidative stress resulting from short-term starvation by utilizing multiple antioxidant systems, although this resistance is likely to attenuate with prolonged fasting.

### 4.3. Effects of Starvation on the Muscular Histology of *Synechogobius hasta*

Similar to the muscle morphological alterations reported in several fish species under food deprivation [40, 42, 43], the histological appearance of muscle was evidently affected after 7 and 14 days of starvation (Figure 4), with muscle tissues exhibiting larger interlobular intervals and atrophy of some muscle trabeculae. Moreover, these findings were consistent with the dynamic decline in the myofiber dimension of starved *S. hasta* with extended fasting time (Figure 4). Given that decreases in energy anabolism and increases in catabolism have been confirmed to weaken muscle function and myofiber activity, resulting in a reduction in muscle size [42, 44], the abnormal muscle structure in *S. hasta* subjected to more than 7 days of fasting was suggestive of a reduced biosynthesis of endogenous reserves, impaired contractility, and growth retardation in *S. hasta* muscle. This assumption was partly supported by the transcriptional expression of lipid metabolism-related genes observed in our study. Muscle overgrowth induced by short-term fasting/refeeding appears to trigger compensatory growth in fish [43]. However, it is worth noting that the structural impairments induced by severe nutrient deficiency would be irreversible and can further exacerbate muscular dysfunction in fish.

### 4.4. Effects of Starvation on the Expression of Lipid Metabolism-Related Genes in the Muscle of *Synechogobius hasta*

Previous studies have reported that starvation induces downregulation of *scd1* in several fish species at the posttranscriptional and transcriptional level [45, 46]. Similarly, the levels of muscular *scd1* decreased significantly in food-deprived *S. hasta*. However, significant fluctuations in muscular *fas* levels were not observed. The variation patterns of these two genes mirrored our previous findings of *scd1* and *fas* expression in the intestine of starved *S. hasta* [5], which further confirmed the species- and organ-dependent differences in the expression of genes associated with *de novo* lipogenesis in food-deprived teleosts [5, 47, 48]. Considering the vital role of *fas* and *scd1* in lipid biosynthesis, the changing trends observed herein indicated that two weeks of food deprivation decreased intramuscular lipid accumulation by transcriptionally impeding fatty acid *de novo* synthesis in the muscle tissues of *S. hasta*, especially monounsaturated fatty acids (MUFAs).

Contrary to the starvation-induced enhancement of lipolysis in the nonmuscular tissues of some teleosts [5, 49–51], including our previous data in the gut of starved *S. hasta* [5], the transcript levels of *lpl* and *cpt1a* were consistently downregulated and decreased to their lowest abundance after 14 days of fasting (Figure 5). These responses of *lpl* and *cpt1a* to starvation suggested that the biological processes involved in lipid hydrolysis and fatty acid *β*-oxidation were suppressed in *S. hasta* muscle. Unlike *S. hasta* liver, which can store large amounts of fat under natural conditions [18], white muscle may preferentially consume glycogen deposits to meet energy requirements in situations of nutrient unavailability [6, 36], resulting in an increase in anaerobic glycolysis until muscle glucose is fully depleted due to prolonged starvation. The anaerobic environment of glycolysis might repress the oxidative metabolism of the limited lipid storage in white muscle fibers [52]. However, our findings confirmed the mobilization of stored lipids in the muscle tissues of *S. hasta*. It is presumed that skeletal muscle in *S. hasta*, particularly the white muscle, primarily relies on glycolysis-sourced energy rather than from lipolytic reactions during short-term fasting, which would explain the reduction in lipolysis and fatty acid catabolism observed in our work. This is also further supported by the greater and more rapid decline in muscle glycogen compared to muscle TG observed in our study.

Here, we observed a continuous downregulation of *fatp1* in *S. hasta* muscle, which was consistent with the lowering *lpl* trend described above, thus confirming less mobilization of fatty acids to muscle tissues within short-term starvation. Moreover, these findings solidified the notion that the expression of fatty acid transporter genes cannot only serve as indicators of fatty acid mobilization but also utilization [53]. The insufficient uptake of fatty acids by muscle tissues could barely support the muscular metabolism under normal physiological conditions through highly active fatty acid oxidation and could inevitably cripple the *cpt*-dominated fatty acid oxidative metabolism. The decreased consumption of fatty acids might neutralize the negative effects of nutrient deprivation on the muscle of starved *S. hasta*. In turn, this may contribute to variations in *fabp3* expression due to the biological role of *fabp3* in transferring fatty acids into the mitochondria where they are utilized for *β*-oxidation [54, 55].

Widely acknowledged transcription factors including PPAR*γ* and SREBP1 act on a series of downstream target molecules in the signaling network of lipid synthesis, uptake, and degradation [5, 20, 56], thereby modulating the final fat accumulation. No obvious changes in *srebp1* were observed among any of the fed or unfed groups (Figure 5). These findings were not only consistent with those found in the gut of *S. hasta* from days 0 to 14 of food deprivation [5] but also shared the same integral trends of *fas*, the downstream target gene of *srebp1* in animals [57]. Similarly, a previous study reported that the levels of *srebp1* were also unaffected in the muscle of silver pomfret (*Pampus argenteus*) at both the mRNA and protein levels over the entire duration of a fasting trial [58]. The regulatory role of the PPAR *γ* pathway on lipid metabolism is different from that in SREBP1 signaling, possessing a more predominant influence on lipid synthesis, transport, and utilization in the muscle and other metabolically active organs [5, 59]. Thus, *ppar γ* in *S. hasta* muscle appeared to be more sensitive to nutritional status and its mRNA level decreased with prolonged starvation. Previous studies have also characterized the similar expression dynamics of *ppar γ* in the intestine of *S. hasta* [5]. Moreover, the downregulation of *ppar γ* was congruent with the decrease in *scd1*, *lpl*, *cpt1a*, and *fatp1* expression in *S. hasta* muscle, which partially confirmed the comprehensive roles of PPAR *γ* in muscle lipid homeostasis in teleosts and mammals [60–62]. Based on the aforementioned observations, we speculated that fasting for less than 14 days decreased lipid homeostasis in *S. hasta* skeletal muscle not only by repressing lipid anabolism and uptake but also by attenuating lipolysis and fat oxidation, which were more apparent than the depression of lipogenic processes.

### 4.5. Effects of Starvation on Transcriptomic Characteristics in the Muscle of *Synechogobius hasta*

Due to advances in sequencing platforms and sequencing depth, 79,255 unigenes were identified in *S. hasta* subjected to different starvation periods, higher than those reported in *S. hasta* exposed to waterborne copper (60,217 unigenes) [63]. As shown in our BLASTx hit analyses (Figure 6), the top similarity was with *Boleophthalmus pectinirostris* (49.7%). Interestingly, a previous study reported that the matched sequences in *S. hasta* shared the highest degree of homology with *Oreochromis niloticus* (49.5%) [63]. *B. pectinirostris* and *S. hasta* belong to the Gobioidei suborder and the Gobiidae family, which would explain their genetic, morphological, and behavioral traits. In contrast, *O. niloticus* belongs to the Cichlidae family and, therefore, is only distantly related to *S. hasta*.

The starved *S. hasta* exhibited more significantly enriched GO terms of DEGs in the BP category (Figure 7; Table S3), which was consistent with previously published data in food-deprived *Larimichthys crocea* [64] and *Nibea albiflora* [65]. Among the overwhelmingly enriched BP category, “cellular process,” “metabolic process,” and “single-organism process” were the top three subclasses with the largest amount of DEGs, which agreed with previous studies on starved carnivorous fish [65, 66]. Furthermore, the enriched GO terms contained much more downregulated unigenes compared to MS *vs*. MC and ML *vs*. MC (Table S3), indicating the suppression of anabolic pathways of energetic substrates or nutrients in *S. hasta* muscle under fasting. These data may suggest the dominance of metabolism-related signaling among all DEGs, which solidifies the long-held notion that fasting affects the expression of genes associated with metabolic processes [67].

Only the “Tight junction” pathway was significantly enriched in all DEGs of MS *vs*. MC (Figure 8, Table S4). Intriguingly, the downregulated DEGs were found to be highly enriched in up to eight KEGG pathways (Figure S3, Table S4). Similar KEGG results were also observed in the downregulated DEGs of ML *vs*. MC, in which 13 significantly enriched pathways related to metabolism and diseases were identified (Figure S3, Table S4). Moreover, five pathways were common to the downregulated genes between the control and starved groups, including “Ribosome,” “Pyruvate metabolism,” “TCA cycle,” “Porphyrin and chlorophyll metabolism,”, and “Glycolysis/Gluconeogenesis” (Table S4). In fact, we expected to observe a decline in metabolism-associated pathways during nutrient deprivation because the synthesis of the three major macronutrients (carbohydrates, lipids, and proteins) and related substrates is an extremely energy-consuming biological process. These findings were supported by the KEGG enrichment data in other food-deprived Perciformes species [64, 68].

Among the five common pathways, the TCA cycle is central to the final metabolic pathway of the three major energy sources [69]. These substrates generated from energy stores may participate in the TCA cycle, an aerobic process for producing ATP by oxidating acetate derived from energy reserves [64, 70], thereby satisfying the energy demands for fish. In our study, eight unigenes that belonged to the TCA pathway between MS and MC were downregulated, including *aco*, *dlst*, *idh*3, and *sdha* (Table S4). As an NADP-linked dehydrogenase, Idh and its isoform can transform isocitrate into *α*-ketoglutarate and CO_2_ in the cytosol and mitochondria [71, 72], which participates in the resistance against oxidative damage, ROS degradation, and lipid synthesis [72, 73]. The declined *idh3* expression in *S. hasta* muscle suggested the reduced NADPH supply under food-deprived conditions, which further suppressed anabolic processes for major energy reserves in muscle tissue, especially lipid synthesis. In fact, the diminished NADPH production trigged by decreased *idh* expression has been reported to lower lipid accumulation *in vivo* and *in vitro* [71]. Similar results regarding starvation-induced *idh* downregulation were found in other fish and nonfish organisms [73–75].

*Sdha,* as a marker of muscular aerobic metabolism in animals, encodes the key multimeric enzyme located in the inner mitochondrial membrane for the oxidation of succinate to fumarate [76–78]. The lower level of *sdha* in MS indicated the attenuation of aerobic capacity in the muscle tissues of *S. hasta* at three days of fasting, which was more evident at 14 days of starvation. The succinate accumulation triggered by the downregulation of *sdha* would disturb the mitochondrial respiratory chain and other TCA cycle-related signals [76–79], thereby impairing muscle function in starved *S. hasta*. The TCA cycle and other metabolic pathways in ML *vs*. MC tended to be similarly affected, showing more downregulated genes (Table S4). Similar transcriptional modifications of DEGs associated with the TCA cycle and other energy anabolic pathways were also reported in other fish species in response to starvation [7, 64, 68, 80]. Therefore, these transcriptional decreases in metabolic pathways might indicate a decreased reliance on ATP for the production of energy-related metabolites during starvation, supporting previous findings in fish and other organisms under fasting conditions [64, 81, 82].

## 5. Conclusion

Our findings demonstrated that the contents of muscle glycogen and TG were gradually decreased in starved *S. hasta*, which was accompanied by a transient compensatory elevation in muscle SOD, GSH-Px, and GSH. After seven days of fasting, the myofibers exhibited enlarged spaces, smaller fiber diameters, and other irregular structures, which were indicative of physiological and metabolic impairments in *S. hasta* muscle. Long-term starvation attenuated multiple processes related to muscular lipid metabolism, particularly by transcriptionally lowering fatty acid transport and fat catabolism in *S. hasta*. A total of 79,255 unigenes were generated from *de novo* transcriptome assembly of *S. hasta* muscle, with more DEGs being downregulated. Under fasting conditions, downregulated genes were mapped to signaling pathways mainly associated with energy metabolism. Overall, these findings indicated that *S. hasta* starved for 3-7 days could maintain a relatively stable muscle structure and function by modulating energy metabolism-related pathways. These findings may serve as a basis for the development of effective fasting/refeeding strategies in *S. hasta* farms and provide important insights into the molecular mechanisms underlying the muscle phenotypic adaptations of fish in response to starvation.

## Figures and Tables

**Table 1:** qRT-PCR primers.

Primer	Forward primer (5′-3′)	Reverse primer (5′-3′)
*fas*	CATCATCACTGGAGGTCTTGGA	TACGAATGCCTGATCTGGAAGT
*scd1*	GACAACCAGCCCAAATCC	GAGCCCCATCAGAAAGAC
*lpl*	AGTCCGATCAACACGAAGC	GGTGCCGTTCCCATTTAG
*cpt1a*	CGCTCCTGCTCCAATGAGA	GAGACCACATAGAGGCAGAAGA
*fatp1*	CCACTGGGCTCAGAATCAAG	CAAGTTCAGCTCCAAAGACAATA
*fabp3*	TGGCTGAAGCATTTGTTGGTGG	GTCATCGGCAGTGGTCTCGT
*srebp1*	TGCTATGCGGAGGTTATTCATC	GTTGCTCTGCGTCGTAGTG
*ppar γ*	TTCTTCCACAGTTGCCAGTC	GTTCATCAGAGGAGCCATCA
*ahnak*	ATCGCCACTGAGGAGAAT	AGGAGACAGCAGACTTACT
*comp*	CCACTTGTAGGAGGTCTTG	GAACCAGGCATCCAACTC
*gk*	TCTGAGGACTGAACAATGC	GCTGAAGAACACTGGAACT
*ltbp1*	GCCTCGCACTGCCAAGAT	GGCACGAGCACCTGAAACT
*mapk9*	TTACCACATTCACCACAGT	CTCCTCCTCTTCTTCCATAC
*mct1*	CTTCGCACTCCTCTTGGG	CAGCGTCATCCTCTTCATCT
*mhc*	TTGGTTTGAGGAGGATGA	GCTTAGGAGGCTGAGGAA
*prkaa2*	ATGTAGAAGACGCCTCCT	GCTCCAGAAGTCATCTCC
*rxrβ*	TCCAATGCCGTCTCAGAA	CAGGTGTAGGTCAGGTCTT
*sfrp1*	CGCACCTTCAACAGACTT	GAACTCCTACGCTCTAATCC
*slc6a6*	AGTGGACTTGACGCCTTTC	CCGCAAGAACAAGACCCT
*slc40a1*	TGAACTACCTGCTGGACC	GAGACGGAGATGATGACG
*β-Actin*	GTGCGTGACATCAAGGAGAAG	CGAGGAAGGATGGCTGGAA
*gapdh*	GCCTCCTGCACCACAAACT	GGACCATCCACGGTCTTCT
*18* s	TTCGATGGTACTTTCTGTGC	CTGCCTTCCTTGGATGTG

Note: the primers sequences for the validation of DEGs in this study were designed using the Primer Premier 5. The primer sequences of the other genes in this study were described in previous studies [5, 17, 23] or designed by Primer Premier 5 software. The abbreviations in Table 1 were as follows: *fas*: fatty acid synthase; *scd1*: stearoyl-CoA desaturase 1; *lpl*: lipoprotein lipase; *cpt1a*: carnitine palmitoyltransferase 1 alpha; *fatp1*: fatty acid transport protein 1; *fabp3*: fatty acid binding protein 3; *srebp1*: sterol regulatory element binding protein 1; *ppar γ*: peroxisome proliferator-activated receptor *γ*; *ahnak*: neuroblast differentiation-associated protein (AHNAK); *comp*: cartilage oligomeric matrix protein; *gk*: glycerol kinase-like isoform X1; *ltbp1*: latent-transforming growth factor beta-binding protein 1-like; *mapk9*: mitogen-activated protein kinase 9-like isoform X2; *mct1*: monocarboxylate transporter 1-like; *mhc*: myosin heavy chain, fast skeletal muscle-like; *prkaa2*: 5′-AMP-activated protein kinase catalytic subunit alpha-2; *rxrβ*: retinoic acid receptor RXR-beta-A isoform X1; *sfrp1*: secreted frizzled-related protein 1; *slc6a6*: sodium- and chloride-dependent taurine transporter-like isoform X2; *slc40a1*: solute carrier family 40 member 1; *β-actin*: beta-actin; *gapdh*: glyceraldehyde 3-phosphate dehydrogenase.

**Table 2:** Summary of the muscular transcriptome data in this study.

Group	Raw reads	Clean reads	Clean bases	Error (%)	Q20 (%)	Q30 (%)	GC (%)	Total reads	Total mapped	Mapping rate
MC	45,097,573	43,622,635	6.54 G	0.03	96.79	91.87	49.79	43,622,635	36,626,787	83.96%
MS	44,501,531	43,602,655	6.54 G	0.03	96.93	92.06	51.11	43,602,655	36,688,381	84.14%
ML	45,661,920	44,420,961	6.67 G	0.03	97.27	92.81	51.03	44,420,961	37,258,603	83.88%

Note: muscle samples in the control group were named as MC (continuously fed for 14 days). Muscle samples in the starvation groups were named as MS (starved for 3 days) and ML (starved for 14 days), respectively. Three replicates were used for each group in the transcriptome analysis. All data are presented as the mean of three replicate tissues from the same group.

**Table 3:** Summary of *de novo* assembly results of the muscular transcriptome.

Term	Total number	Mean length	Min length	Max length	N50	N90	Total nucleotides
Unigene	79,255	1,274	301	60,817	2,333	477	100,953,031
Transcripts	196,071	1,852	301	60,817	3,348	744	363,183,232

**Table 4:** Summary of annotation results of the muscular transcriptome.

Description	Number of unigenes	Percentage (%)
Annotated in Nr	30,676	38.70
Annotated in Nt	34,826	43.94
Annotated in Pfam	31,158	39.31
Annotated in KOG	12,220	15.41
Annotated in the Swiss-Prot	28,513	35.97
Annotated in GO	31,158	39.31
Annotated in KEGG	7,814	9.85
Annotated in all databases	3,732	4.70
Annotated in at least one database	47,109	59.43
All unigenes	79,255	100

Note: percentage represents the ratio of the mapped unigenes among all assembled unigenes.

## Data Availability

All data that support the findings of this study are available from the corresponding author upon reasonable request.

## Supplementary Materials

Supplementary materials Table S1: statistics of *S. hasta* transcriptome data in this study. Table S2: statistics of DEGs in this study. Table S3: GO analysis of DEGs in this study. Table S4: KEGG pathway analysis of DEGs in this study. Table S5: information on 12 representative DEGs determined by RNA-seq. Figure S1: Venn diagram of annotation results in *Synechogobius hasta* against five common databases. Figure S2: GO enrichment analysis of DEGs in the muscle tissues of *Synechogobius hasta*. Figure S3: KEGG pathway enrichment analysis of upregulated DEGs and downregulated DEGs in the muscle tissues of *Synechogobius hasta*. Figure S4: effect of starvation on the expression of selected DEGs in the muscle tissues of *Synechogobius hasta*. Supplementary method. Method S1: cDNA library construction and sequencing.
