# Supplementary material for: Starvation Affects the Muscular Morphology, Antioxidant Enzyme Activity, Expression of Lipid Metabolism-Related Genes, and Transcriptomic Profile of Javelin Goby (Synechogobius hasta)
Source: Aquac Nutr. 2022 Dec 30;2022:7057571. doi: 10.1155/2022/7057571 (PMC9973160; doi:10.1155/2022/7057571)
Supplement: Supplementary materials — Table S1: statistics of S. hasta transcriptome data in this study. Table S2: statistics of DEGs in this study. Table S3: GO analysis of DEGs in this study. Table S4: KEGG pathway analysis of DEGs in this study. Table S5: information on 12 representative DEGs determined by RNA-seq. Figure S1: Venn diagram of annotation results in Synechogobius hasta against five common databases. Figure S2: GO enrichment analysis of DEGs in the muscle tissues of Synechogobius hasta. Figure S3: KEGG pathway enrichment analysis of upregulated DEGs and downregulated DEGs in the muscle tissues of Synechogobius hasta. Figure S4: effect of starvation on the expression of selected DEGs in the muscle tissues of Synechogobius hasta. Supplementary method. Method S1: cDNA library construction and sequencing. [file 7057571.f1.zip › 7057571.f1/Figure S1-4 for AN Proof.docx]

**Fig.S1.**


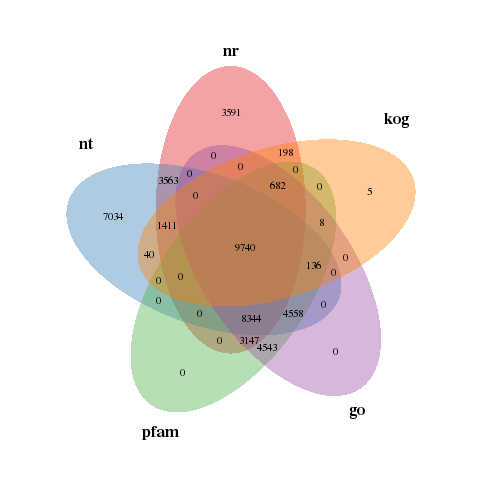


**Fig.S1. Venn diagram of annotation results in *Synechogobius hasta* against five common databases.**

Annotation results of all unigenes in *Synechogobius hasta* against the NT, NR, KOG, GO, and PFAM databases.

Note:

NT, NCBI nucleotide sequences; NR, NCBI non-redundant; KOG, euClusters of Orthologous Groups; GO, Gene Ontology; PFAM, Protein family.

**Fig.S2.**


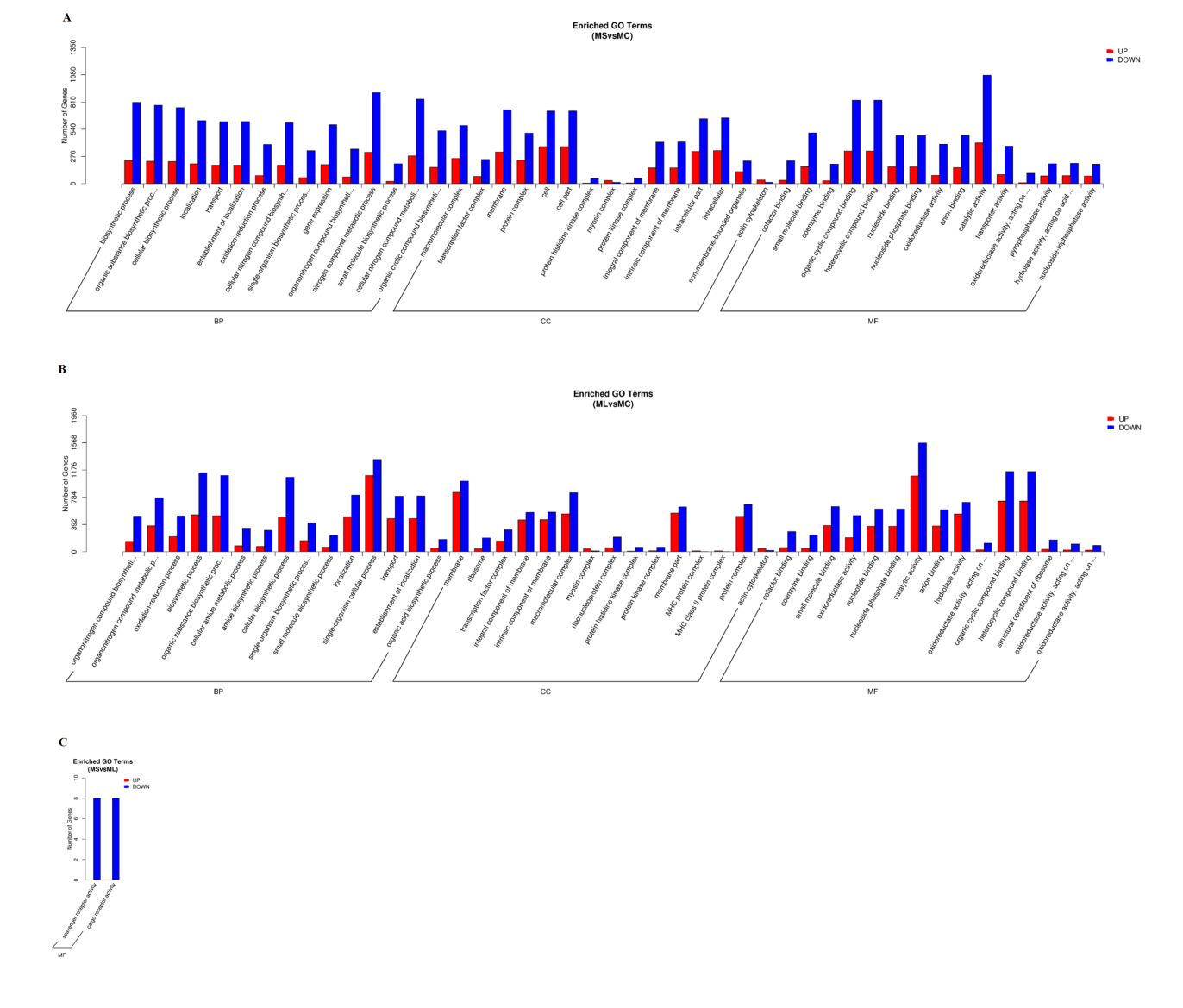


**Fig.S2. GO enrichment analysis of the differentially expressed genes (DEGs) in the muscle tissues of *Synechogobius hasta*.**

The GO classification of DEGs in the comparison of MS *vs.* MC (A), ML *vs.* MC (B), and ML *vs.* MS (C).

The horizontal axis denotes the number of DEGs with a significantly enriched GO term (corrected *P* ≤ 0.05). The vertical axis represents the significantly enriched GO term. The GO terms are grouped into three categories: biological process (BP), cellular component (CC), and molecular function (MF). Red bar represents upregulated genes, and blue bar represents downregulated genes.

MC: Muscle in the control group (continuously fed for 14 days); MS: Muscle in the starved group (starved for 3 days); ML: Muscle in the starved group (starved for 14 days).

**Fig.S3.**


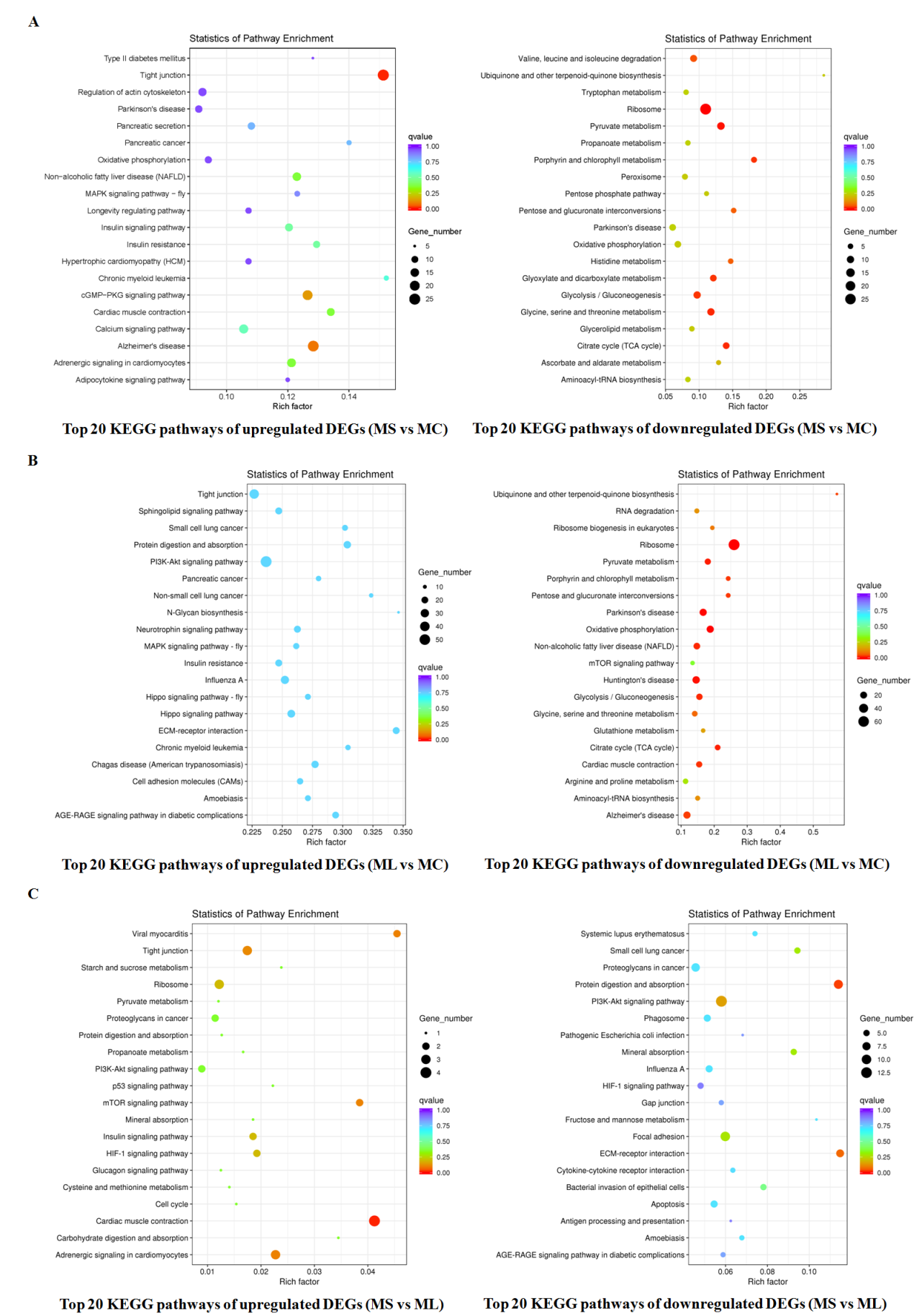


**Fig.S3. KEGG pathway enrichment analysis of upregulated DEGs and downregulated DEGs in the muscle tissues of *Synechogobius hasta*.**

The top 20 of KEGG enrichment of upregulated DEGs and downregulated DEGs in the comparison of MS *vs.* MC (A), ML *vs.* MC (B), and ML *vs.* MS (C).

The horizontal axis denotes Rich factor, the ratio of upregulated DEGs and downregulated DEGs with a specific pathway term relative to all DEGs. The vertical axis denotes the annotated KEGG pathway. The color and size of the bubbles represent the number of corrected *P*-value and DEGs with a specific KEGG pathway, respectively.

MC: Muscle in the control group (continuously fed for 14 days); MS: Muscle in the starved group (starved for 3 days); ML: Muscle in the starved group (starved for 14 days).

**Fig.S4.**


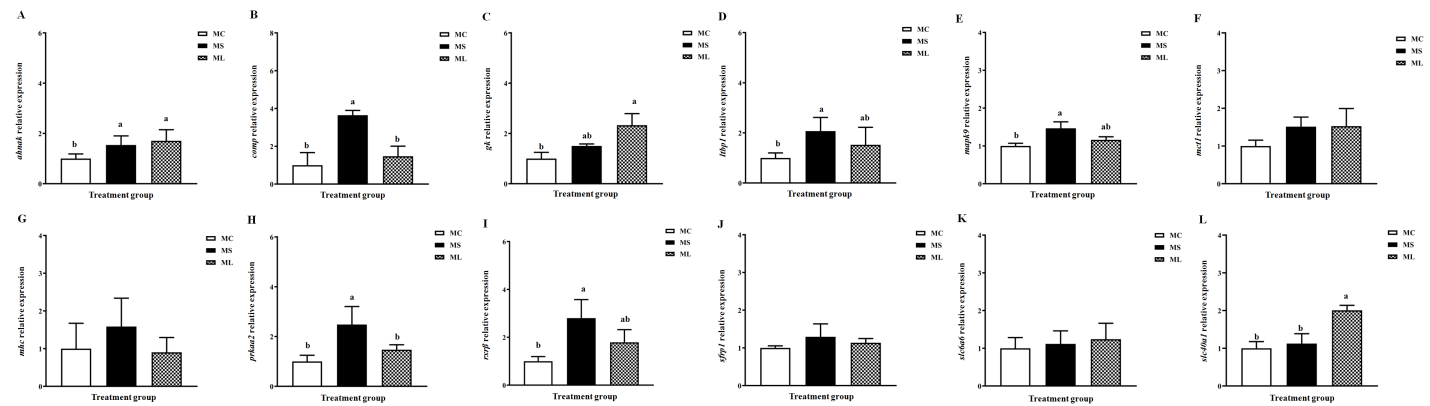


**Fig.S4. Effect of starvation on the expression of selected DEGs in the muscle tissues of *Synechogobius hasta.***

Relative mRNA expression levels of *ahnak* (A), *comp* (B), *gk* (C), *ltbp1* (D), *mapk9* (E), *mct1* (F), *mhc* (G), *prkaa2* (H), *rxrβ* (I), *sfrp1* (J), *slc6a6* (K), and *slc40a1* (L) in the muscle of *S. hasta*. The relative gene expression was determined via quantitative real-time PCR (qRT-PCR) and normalized to the expression of a house-keeping gene (*β-actin*). All data were expressed as mean ± SD (n = 3) and differences between groups were determined via one-way ANOVA. Columns with different letters were significantly different (*P* < 0.05; *P* < 0.01).

MC: Muscle in the control group (continuously fed for 14 days); MS: Muscle in the starved group (starved for 3 days); ML: Muscle in the starved group (starved for 14 days).

The abbreviations in Fig.S4 were used as follows:

*ahnak*: neuroblast differentiation-associated protein AHNAK; *comp*: cartilage oligomeric matrix protein; *gk*: glycerol kinase-like isoform X1; *ltbp1*: latent-transforming growth factor beta-binding protein 1-like; *mapk9*: mitogen-activated protein kinase 9-like isoform X2; *mct1*: monocarboxylate transporter 1-like; *mhc*: myosin heavy chain, fast skeletal muscle-like; *prkaa2*: 5'-AMP-activated protein kinase catalytic subunit alpha-2; *rxrβ*: retinoic acid receptor RXR-beta-A isoform X1; *sfrp1*: secreted frizzled-related protein 1; *slc6a6*: sodium- and chloride-dependent taurine transporter-like isoform X2; *slc40a1*: solute carrier family 40 member 1.
