# Supplementary material for: Starvation Affects the Muscular Morphology, Antioxidant Enzyme Activity, Expression of Lipid Metabolism-Related Genes, and Transcriptomic Profile of Javelin Goby (Synechogobius hasta)
Source: Aquac Nutr. 2022 Dec 30;2022:7057571. doi: 10.1155/2022/7057571 (PMC9973160; doi:10.1155/2022/7057571)
Supplement: Supplementary materials — Table S1: statistics of S. hasta transcriptome data in this study. Table S2: statistics of DEGs in this study. Table S3: GO analysis of DEGs in this study. Table S4: KEGG pathway analysis of DEGs in this study. Table S5: information on 12 representative DEGs determined by RNA-seq. Figure S1: Venn diagram of annotation results in Synechogobius hasta against five common databases. Figure S2: GO enrichment analysis of DEGs in the muscle tissues of Synechogobius hasta. Figure S3: KEGG pathway enrichment analysis of upregulated DEGs and downregulated DEGs in the muscle tissues of Synechogobius hasta. Figure S4: effect of starvation on the expression of selected DEGs in the muscle tissues of Synechogobius hasta. Supplementary method. Method S1: cDNA library construction and sequencing. [file 7057571.f1.zip › 7057571.f1/Supplementary Method For cDNA library construction & sequencing.docx]

**Supplementary Method**

**The procedures of cDNA library construction and sequencing**

High-quality RNA samples (OD260/OD280 = 1.98–2.10, RIN = 7.8–9.9) were pooled for cDNA library construction and then sequenced by Novogene Co., Ltd (Beijing, China) on an Illumina Hiseq sequencer.

The brief steps are as follows.

The total RNA (≥ 1.5 μg) of *S.hasta* muscle was utilized as input material for library construction using the NEBNext® Ultra^TM^ RNA Library Prep Kit for Illumina® (NEB, Ipswich, MA, USA). The mRNA from total RNA sample was recruited using the magnetic beads with poly-T oligo, followed by fragmentation via divalent cations at elevated temperature. The fragmented mRNA and random hexamer primers were used for cDNA synthesis in the reverse-transcription system containing DNA Polymerase I, RNase H, and dNTPs. The double-stranded cDNA was purified with AMPure XP beads (Beckman Coulter, Beverly, MA, USA), and then end repaired by exonuclease/polymerase, followed by the ligation of poly-A tailing and sequencing adapters with hairpin loop structure. The ligation products of preferentially 250-300 bp in size were selected and purified using the AMPure XP system. Subsequently, PCR amplification was employed to obtain the cDNA fragments for the sequencing library.

The eligible sample library was preliminarily adjusted to a 1.5 ng/μL concentration and analyzed via by qRT-PCR, and the effective final concentration of cDNA library was higher than 2 nM. After clustering on a cBot Cluster Generation System, all sample libraries were eventually sequenced on an Illumina Hiseq 2500 sequencer to generate 150 bp paired-end reads.
