# Supplementary material for: Starvation Affects the Muscular Morphology, Antioxidant Enzyme Activity, Expression of Lipid Metabolism-Related Genes, and Transcriptomic Profile of Javelin Goby (Synechogobius hasta)
Source: Aquac Nutr. 2022 Dec 30;2022:7057571. doi: 10.1155/2022/7057571 (PMC9973160; doi:10.1155/2022/7057571)
Supplement: Supplementary materials — Table S1: statistics of S. hasta transcriptome data in this study. Table S2: statistics of DEGs in this study. Table S3: GO analysis of DEGs in this study. Table S4: KEGG pathway analysis of DEGs in this study. Table S5: information on 12 representative DEGs determined by RNA-seq. Figure S1: Venn diagram of annotation results in Synechogobius hasta against five common databases. Figure S2: GO enrichment analysis of DEGs in the muscle tissues of Synechogobius hasta. Figure S3: KEGG pathway enrichment analysis of upregulated DEGs and downregulated DEGs in the muscle tissues of Synechogobius hasta. Figure S4: effect of starvation on the expression of selected DEGs in the muscle tissues of Synechogobius hasta. Supplementary method. Method S1: cDNA library construction and sequencing. [file 7057571.f1.zip › 7057571.f1/Table S1, S2, S5 for AN Proof.docx]

**TABLE S1 Statistics of *S. hasta* transcriptome data in this study.**

| Sample | Raw reads | Clean reads | Clean bases | Error(%) | Q20(%) | Q30(%) | GC(%) | Total reads | Total mapped | Mapping rate |
| --- | --- | --- | --- | --- | --- | --- | --- | --- | --- | --- |
| MC_1 | 47,027,002 | 45,600,902 | 6.84G | 0.03 | 96.66 | 91.59 | 50.10 | 45,600,902 | 38,811,014 | 85.11% |
| MC_2 | 44,708,570 | 42,730,050 | 6.41G | 0.03 | 96.85 | 92.04 | 49.42 | 42,730,050 | 35,643,156 | 83.41% |
| MC_3 | 43,557,146 | 42,536,952 | 6.38G | 0.03 | 96.85 | 91.98 | 49.84 | 42,536,952 | 35,426,190 | 83.28% |
| MS_1 | 42,542,432 | 41,447,976 | 6.22G | 0.03 | 96.75 | 91.75 | 50.94 | 41,447,976 | 34,964,240 | 84.36% |
| MS_2 | 42,951,582 | 42,193,664 | 6.33G | 0.03 | 96.90 | 92.03 | 51.32 | 42,193,664 | 35,511,424 | 84.16% |
| MS_3 | 48,010,578 | 47,166,326 | 7.07G | 0.03 | 97.13 | 92.39 | 51.08 | 47,166,326 | 39,589,480 | 83.94% |
| ML_1 | 39,025,592 | 38,050,876 | 5.71G | 0.03 | 97.50 | 93.34 | 51.63 | 38,050,876 | 32,172,760 | 84.55% |
| ML_2 | 53,369,530 | 51,511,026 | 7.73G | 0.03 | 97.31 | 92.84 | 51.07 | 51,511,026 | 43,541,556 | 84.53% |
| ML_3 | 44,590,638 | 43,700,982 | 6.56G | 0.03 | 97.01 | 92.26 | 50.40 | 43,700,982 | 36,061,494 | 82.52% |

Note:

Muscle samples in the control group were named as MC (continuously fed for 14 days). Muscle samples in the starvation groups were named as MS (starved for 3 days) and ML (starved for 14 days), respectively. Three replicates were used for each group in the transcriptome analysis.

**TABLE S2 Statistics of differentially expressed genes (DEGs) in this study.**

| Group | DEGs | | |
| --- | --- | --- | --- |
|  | Up-regulated | Down-regulated | Total |
| MS vs MC | 1162 | 2114 | 3276 |
| ML vs MC | 3155 | 4199 | 7354 |
| MS vs ML | 201 | 341 | 542 |

Note:

Muscle samples in the control group were named as MC (continuously fed for 14 days). Muscle samples in the starvation groups were named as MS (starved for 3 days) and ML (starved for 14 days), respectively. DEGs between two groups were screened at |log2(fold change)| ≥1 and *P* < 0.05.

**TABLE S5 Information on 12 representative DEGs determined by RNA-seq.**

| Gene | Description | Log2 (FC) | | Corrected *P* |
| --- | --- | --- | --- | --- |
|  |  | MS vs MC | ML vs MC |  |
| *ahnak* | neuroblast differentiation-associated protein AHNAK | 1.62 |  | 0.04 |
|  |  |  | 5.47 | <0.01 |
| *comp* | cartilage oligomeric matrix protein | 2.05 |  | <0.01 |
|  |  |  | 3.75 | <0.01 |
| *gk* | glycerol kinase-like isoform X1 | 1.58 |  | 0.02 |
|  |  |  | 2.10 | <0.01 |
| *ltbp1* | latent-transforming growth factor beta-binding protein 1-like | 2.47 |  | <0.01 |
|  |  |  | 4.44 | <0.01 |
| *mapk9* | mitogen-activated protein kinase 9-like isoform X2 | 1.84 |  | 0.02 |
|  |  |  | 2.70 | <0.01 |
| *mct1* | monocarboxylate transporter 1-like | 1.91 |  | 0.03 |
|  |  |  | 4.12 | <0.01 |
| *mhc* | myosin heavy chain, fast skeletal muscle-like | -3.37 |  | <0.01 |
|  |  |  | -5.22 | <0.01 |
| *prkaa2* | 5'-AMP-activated protein kinase catalytic subunit alpha-2 | 3.27 |  | <0.01 |
|  |  |  | 3.77 | <0.01 |
| *rxrβ* | retinoic acid receptor RXR-beta-A isoform X1 | 2.83 |  | <0.01 |
|  |  |  | 3.83 | <0.01 |
| *sfrp1* | secreted frizzled-related protein 1 | 2.61 |  | <0.01 |
|  |  |  | 5.15 | <0.01 |
| *slc6a6* | sodium- and chloride-dependent taurine transporter-like isoform X2 | 3.02 |  | <0.01 |
|  |  |  | 4.74 | <0.01 |
| *slc40a1* | solute carrier family 40 member 1 | 2.84 |  | 0.04 |
|  |  |  | 5.28 | <0.01 |

Note:

Muscle samples in the control group were named as MC (continuously fed for 14 days). Muscle samples in the starvation groups were named as MS (starved for 3 days) and ML (starved for 14 days), respectively. Gene names (|log2(FC)| ≥ 1 and *P* < 0.05) and fold changes in the transcript abundance of these genes was measured by RNA-seq.

The abbreviations in Table S5 were as follows:

*ahnak*: neuroblast differentiation-associated protein AHNAK; *comp*: cartilage oligomeric matrix protein; *gk*: glycerol kinase-like isoform X1; *ltbp1*: latent-transforming growth factor beta-binding protein 1-like; *mapk9*: mitogen-activated protein kinase 9-like isoform X2; *mct1*: monocarboxylate transporter 1-like; *mhc*: myosin heavy chain, fast skeletal muscle-like; *prkaa2*: 5'-AMP-activated protein kinase catalytic subunit alpha-2; *rxrβ*: retinoic acid receptor RXR-beta-A isoform X1; *sfrp1*: secreted frizzled-related protein 1; *slc6a6*: sodium- and chloride-dependent taurine transporter-like isoform X2; *slc40a1*: solute carrier family 40 member 1.
