# Supplementary material for: Starvation Affects the Muscular Morphology, Antioxidant Enzyme Activity, Expression of Lipid Metabolism-Related Genes, and Transcriptomic Profile of Javelin Goby (Synechogobius hasta)
Source: Aquac Nutr. 2022 Dec 30;2022:7057571. doi: 10.1155/2022/7057571 (PMC9973160; doi:10.1155/2022/7057571)
Supplement: Supplementary materials — Table S1: statistics of S. hasta transcriptome data in this study. Table S2: statistics of DEGs in this study. Table S3: GO analysis of DEGs in this study. Table S4: KEGG pathway analysis of DEGs in this study. Table S5: information on 12 representative DEGs determined by RNA-seq. Figure S1: Venn diagram of annotation results in Synechogobius hasta against five common databases. Figure S2: GO enrichment analysis of DEGs in the muscle tissues of Synechogobius hasta. Figure S3: KEGG pathway enrichment analysis of upregulated DEGs and downregulated DEGs in the muscle tissues of Synechogobius hasta. Figure S4: effect of starvation on the expression of selected DEGs in the muscle tissues of Synechogobius hasta. Supplementary method. Method S1: cDNA library construction and sequencing. [file 7057571.f1.zip › 7057571.f1/TABLE S3 GO analysis of DEGs in this study.doc]

| **TABLE S3 GO analysis of DEGs in this study.** | | | | | |  | |  | | |  | | |  |  |
| --- | --- | --- | --- | --- | --- | --- | --- | --- | --- | --- | --- | --- | --- | --- | --- |
|  |  |  | | | |  | |  | | |  | | |  |  |
| **Table S3A. GO analysis of DEGs in the muscle between MS vs MC (TOP20).** | | | | | | | | | | |  | | |  |  |
| Category | GO ID | Description | pvalue | padj | Up Gene Count | | Down Gene Count | | |  | | |  | | |
| BP | GO:0009058 | biosynthetic process | 2.2244E-47 | 1.111E-43 | 228 | | 806 | | |  | | |  | | |
| BP | GO:1901576 | organic substance biosynthetic process | 7.9511E-45 | 1.3403E-41 | 222 | | 777 | | |  | | |  | | |
| BP | GO:0044249 | cellular biosynthetic process | 9.8531E-43 | 8.3045E-40 | 219 | | 754 | | |  | | |  | | |
| BP | GO:0051179 | localization | 3.0996E-37 | 1.425E-34 | 196 | | 625 | | |  | | |  | | |
| BP | GO:0006810 | transport | 2.5216E-36 | 8.8286E-34 | 183 | | 614 | | |  | | |  | | |
| BP | GO:0051234 | establishment of localization | 2.6187E-36 | 8.8286E-34 | 183 | | 616 | | |  | | |  | | |
| BP | GO:0055114 | oxidation-reduction process | 1.27E-33 | 4.0141E-31 | 80 | | 389 | | |  | | |  | | |
| BP | GO:0044271 | cellular nitrogen compound biosynthetic process | 1.5152E-33 | 4.5073E-31 | 183 | | 604 | | |  | | |  | | |
| BP | GO:0044711 | single-organism biosynthetic process | 2.9871E-33 | 8.392E-31 | 59 | | 327 | | |  | | |  | | |
| BP | GO:0010467 | gene expression | 7.927E-32 | 2.1098E-29 | 189 | | 584 | | |  | | |  | | |
| BP | GO:1901566 | organonitrogen compound biosynthetic process | 1.2266E-31 | 2.9538E-29 | 66 | | 343 | | |  | | |  | | |
| BP | GO:0006807 | nitrogen compound metabolic process | 4.7599E-31 | 1.0941E-28 | 311 | | 902 | | |  | | |  | | |
| BP | GO:0044283 | small molecule biosynthetic process | 1.0724E-30 | 2.3579E-28 | 23 | | 197 | | |  | | |  | | |
| BP | GO:0034641 | cellular nitrogen compound metabolic process | 5.7269E-30 | 1.2067E-27 | 275 | | 838 | | |  | | |  | | |
| BP | GO:1901362 | organic cyclic compound biosynthetic process | 2.8348E-29 | 5.7343E-27 | 162 | | 524 | | |  | | |  | | |
| BP | GO:1901564 | organonitrogen compound metabolic process | 1.5154E-28 | 2.9475E-26 | 149 | | 527 | | |  | | |  | | |
| BP | GO:0009059 | macromolecule biosynthetic process | 7.9408E-28 | 1.4873E-25 | 182 | | 577 | | |  | | |  | | |
| BP | GO:0018130 | heterocycle biosynthetic process | 3.2564E-27 | 5.6785E-25 | 158 | | 510 | | |  | | |  | | |
| BP | GO:0044710 | single-organism metabolic process | 4.226E-27 | 7.1236E-25 | 208 | | 722 | | |  | | |  | | |
| BP | GO:0034645 | cellular macromolecule biosynthetic process | 5.4978E-27 | 8.9685E-25 | 181 | | 568 | | |  | | |  | | |
| CC | GO:0032991 | macromolecular complex | 7.2563E-21 | 5.4603E-19 | 250 | | 576 | | |  | | |  | | |
| CC | GO:0005667 | transcription factor complex | 1.036E-20 | 7.5931E-19 | 71 | | 240 | | |  | | |  | | |
| CC | GO:0016020 | membrane | 6.2562E-20 | 4.2183E-18 | 314 | | 731 | | |  | | |  | | |
| CC | GO:0043234 | protein complex | 1.3281E-18 | 7.0695E-17 | 232 | | 501 | | |  | | |  | | |
| CC | GO:0005623 | cell | 2.4405E-14 | 9.3496E-13 | 366 | | 721 | | |  | | |  | | |
| CC | GO:0044464 | cell part | 2.4405E-14 | 9.3496E-13 | 366 | | 721 | | |  | | |  | | |
| CC | GO:0009365 | protein histidine kinase complex | 6.6063E-13 | 2.0005E-11 | 4 | | 53 | | |  | | |  | | |
| CC | GO:0016459 | myosin complex | 1.1189E-12 | 3.348E-11 | 32 | | 12 | | |  | | |  | | |
| CC | GO:1902911 | protein kinase complex | 1.0742E-11 | 3.0122E-10 | 6 | | 55 | | |  | | |  | | |
| CC | GO:0016021 | integral component of membrane | 4.6738E-11 | 1.2374E-09 | 156 | | 412 | | |  | | |  | | |
| CC | GO:0031224 | intrinsic component of membrane | 8.5181E-11 | 2.1978E-09 | 157 | | 414 | | |  | | |  | | |
| CC | GO:0044424 | intracellular part | 1.0443E-10 | 2.6808E-09 | 319 | | 643 | | |  | | |  | | |
| CC | GO:0005622 | intracellular | 2.1737E-10 | 5.3621E-09 | 328 | | 653 | | |  | | |  | | |
| CC | GO:0043228 | non-membrane-bounded organelle | 1.8362E-09 | 4.3391E-08 | 119 | | 226 | | |  | | |  | | |
| CC | GO:0015629 | actin cytoskeleton | 3.7634E-09 | 8.7301E-08 | 37 | | 13 | | |  | | |  | | |
| CC | GO:1902494 | catalytic complex | 4.3733E-09 | 1.0098E-07 | 82 | | 173 | | |  | | |  | | |
| CC | GO:0043232 | intracellular non-membrane-bounded organelle | 5.1589E-09 | 1.1805E-07 | 118 | | 217 | | |  | | |  | | |
| CC | GO:0044425 | membrane part | 1.9521E-08 | 4.0518E-07 | 196 | | 461 | | |  | | |  | | |
| CC | GO:0019867 | outer membrane | 3.7692E-08 | 7.5112E-07 | 21 | | 59 | | |  | | |  | | |
| CC | GO:0005840 | ribosome | 3.506E-07 | 6.2871E-06 | 22 | | 98 | | |  | | |  | | |
| MF | GO:0048037 | cofactor binding | 4.3939E-47 | 1.111E-43 | 33 | | 227 | | |  | | |  | | |
| MF | GO:0036094 | small molecule binding | 6.1947E-44 | 7.8316E-41 | 169 | | 503 | | |  | | |  | | |
| MF | GO:0050662 | coenzyme binding | 8.739E-44 | 8.8387E-41 | 28 | | 194 | | |  | | |  | | |
| MF | GO:0097159 | organic cyclic compound binding | 4.4476E-41 | 2.8114E-38 | 323 | | 827 | | |  | | |  | | |
| MF | GO:1901363 | heterocyclic compound binding | 4.4476E-41 | 2.8114E-38 | 323 | | 827 | | |  | | |  | | |
| MF | GO:0000166 | nucleotide binding | 3.686E-40 | 1.864E-37 | 166 | | 477 | | |  | | |  | | |
| MF | GO:1901265 | nucleoside phosphate binding | 3.686E-40 | 1.864E-37 | 166 | | 477 | | |  | | |  | | |
| MF | GO:0016491 | oxidoreductase activity | 3.5417E-37 | 1.4925E-34 | 82 | | 391 | | |  | | |  | | |
| MF | GO:0043168 | anion binding | 8.9197E-37 | 3.4698E-34 | 159 | | 480 | | |  | | |  | | |
| MF | GO:0003824 | catalytic activity | 1.0358E-31 | 2.6191E-29 | 404 | | 1075 | | |  | | |  | | |
| MF | GO:0005215 | transporter activity | 2.9095E-27 | 5.2548E-25 | 89 | | 371 | | |  | | |  | | |
| MF | GO:0016614 | oxidoreductase activity, acting on CH-OH group of donors | 4.6817E-22 | 4.8317E-20 | 9 | | 102 | | |  | | |  | | |
| MF | GO:0016462 | pyrophosphatase activity | 2.0073E-21 | 1.6113E-19 | 76 | | 197 | | |  | | |  | | |
| MF | GO:0016818 | hydrolase activity, acting on acid anhydrides, in phosphorus-containing anhydrides | 3.9135E-21 | 2.9986E-19 | 79 | | 201 | | |  | | |  | | |
| MF | GO:0017111 | nucleoside-triphosphatase activity | 1.1443E-20 | 8.2666E-19 | 75 | | 193 | | |  | | |  | | |
| MF | GO:0032553 | ribonucleotide binding | 4.2524E-20 | 2.906E-18 | 132 | | 352 | | |  | | |  | | |
| MF | GO:0050660 | flavin adenine dinucleotide binding | 1.0103E-19 | 6.6352E-18 | 7 | | 80 | | |  | | |  | | |
| MF | GO:0016817 | hydrolase activity, acting on acid anhydrides | 1.0736E-19 | 6.9608E-18 | 80 | | 215 | | |  | | |  | | |
| MF | GO:0005524 | ATP binding | 1.5762E-19 | 9.8405E-18 | 115 | | 312 | | |  | | |  | | |
| MF | GO:0032559 | adenyl ribonucleotide binding | 2.1746E-19 | 1.3411E-17 | 115 | | 312 | | |  | | |  | | |
|  |  |  | | | |  | |  | | |  | | |  |  |
|  |  |  | | | |  | |  | | |  | | |  |  |
|  |  |  | | | |  | |  | | |  | | |  |  |
| **Table S3B. GO analysis of DEGs in the muscle between ML vs MC (TOP20).** | | | | | | | | | | |  | | |  |  |
| Category | GOID | Description | pvalue | padj | Up Gene Count | | Down Gene Count | | |  | | |  | | |
| BP | GO:1901566 | organonitrogen compound biosynthetic process | 1.76E-23 | 2.97E-20 | 150 | | 512 | | |  | | |  | | |
| BP | GO:1901564 | organonitrogen compound metabolic process | 1.03E-19 | 1.04E-16 | 373 | | 776 | | |  | | |  | | |
| BP | GO:0055114 | oxidation-reduction process | 1.09E-18 | 7.85E-16 | 217 | | 515 | | |  | | |  | | |
| BP | GO:0009058 | biosynthetic process | 4.31E-18 | 2.18E-15 | 531 | | 1137 | | |  | | |  | | |
| BP | GO:1901576 | organic substance biosynthetic process | 1.14E-17 | 5.23E-15 | 518 | | 1097 | | |  | | |  | | |
| BP | GO:0043603 | cellular amide metabolic process | 1.34E-17 | 5.63E-15 | 86 | | 338 | | |  | | |  | | |
| BP | GO:0043604 | amide biosynthetic process | 5.49E-17 | 1.98E-14 | 75 | | 309 | | |  | | |  | | |
| BP | GO:0044249 | cellular biosynthetic process | 6.50E-17 | 2.17E-14 | 502 | | 1072 | | |  | | |  | | |
| BP | GO:0044711 | single-organism biosynthetic process | 6.87E-17 | 2.17E-14 | 159 | | 415 | | |  | | |  | | |
| BP | GO:0044283 | small molecule biosynthetic process | 1.39E-16 | 4.15E-14 | 64 | | 240 | | |  | | |  | | |
| BP | GO:0051179 | localization | 4.42E-15 | 1.18E-12 | 504 | | 815 | | |  | | |  | | |
| BP | GO:0044763 | single-organism cellular process | 1.26E-14 | 3.04E-12 | 1098 | | 1328 | | |  | | |  | | |
| BP | GO:0006810 | transport | 1.36E-14 | 3.12E-12 | 479 | | 799 | | |  | | |  | | |
| BP | GO:0051234 | establishment of localization | 1.71E-14 | 3.75E-12 | 479 | | 802 | | |  | | |  | | |
| BP | GO:0016053 | organic acid biosynthetic process | 3.37E-14 | 6.82E-12 | 53 | | 177 | | |  | | |  | | |
| BP | GO:0046394 | carboxylic acid biosynthetic process | 3.37E-14 | 6.82E-12 | 53 | | 177 | | |  | | |  | | |
| BP | GO:0044710 | single-organism metabolic process | 3.68E-14 | 7.15E-12 | 597 | | 1001 | | |  | | |  | | |
| BP | GO:0044699 | single-organism process | 4.42E-14 | 8.27E-12 | 1306 | | 1594 | | |  | | |  | | |
| BP | GO:0044271 | cellular nitrogen compound biosynthetic process | 4.76E-14 | 8.60E-12 | 389 | | 880 | | |  | | |  | | |
| BP | GO:0022613 | ribonucleoprotein complex biogenesis | 2.45E-13 | 3.68E-11 | 51 | | 209 | | |  | | |  | | |
| CC | GO:0016020 | membrane | 1.03E-12 | 1.33E-10 | 855 | | 1017 | | |  | | |  | | |
| CC | GO:0005840 | ribosome | 2.69E-12 | 3.16E-10 | 42 | | 198 | | |  | | |  | | |
| CC | GO:0005667 | transcription factor complex | 2.15E-10 | 1.73E-08 | 154 | | 316 | | |  | | |  | | |
| CC | GO:0016021 | integral component of membrane | 2.03E-09 | 1.46E-07 | 459 | | 566 | | |  | | |  | | |
| CC | GO:0031224 | intrinsic component of membrane | 3.67E-09 | 2.48E-07 | 462 | | 570 | | |  | | |  | | |
| CC | GO:0032991 | macromolecular complex | 9.43E-09 | 5.48E-07 | 544 | | 850 | | |  | | |  | | |
| CC | GO:0016459 | myosin complex | 6.81E-08 | 3.02E-06 | 43 | | 12 | | |  | | |  | | |
| CC | GO:0030529 | ribonucleoprotein complex | 1.84E-07 | 6.76E-06 | 58 | | 211 | | |  | | |  | | |
| CC | GO:0009365 | protein histidine kinase complex | 3.28E-07 | 1.13E-05 | 8 | | 64 | | |  | | |  | | |
| CC | GO:1902911 | protein kinase complex | 8.51E-05 | 3.08E-06 | 13 | | 66 | | |  | | |  | | |
| CC | GO:0044425 | membrane part | 8.05E-06 | 0.00020387 | 556 | | 645 | | |  | | |  | | |
| CC | GO:0042611 | MHC protein complex | 3.49E-05 | 0.00073909 | 12 | | 1 | | |  | | |  | | |
| CC | GO:0042613 | MHC class II protein complex | 3.49E-05 | 0.00073909 | 12 | | 1 | | |  | | |  | | |
| CC | GO:0043234 | protein complex | 8.89E-05 | 0.0016462 | 510 | | 684 | | |  | | |  | | |
| CC | GO:0015629 | actin cytoskeleton | 0.00036101 | 0.0061389 | 45 | | 18 | | |  | | |  | | |
| CC | GO:0043228 | non-membrane-bounded organelle | 0.00079305 | 0.01234 | 216 | | 360 | | |  | | |  | | |
| CC | GO:0043232 | intracellular non-membrane-bounded organelle | 0.00090666 | 0.013727 | 213 | | 349 | | |  | | |  | | |
| CC | GO:0019867 | outer membrane | 0.0012682 | 0.018299 | 35 | | 78 | | |  | | |  | | |
| CC | GO:0005623 | cell | 0.0019883 | 0.02667 | 811 | | 1085 | | |  | | |  | | |
| CC | GO:0044464 | cell part | 0.0019883 | 0.02667 | 811 | | 1085 | | |  | | |  | | |
| MF | GO:0048037 | cofactor binding | 6.92E-27 | 3.50E-23 | 59 | | 290 | | |  | | |  | | |
| MF | GO:0050662 | coenzyme binding | 5.12E-24 | 1.29E-20 | 47 | | 243 | | |  | | |  | | |
| MF | GO:0036094 | small molecule binding | 1.79E-20 | 2.26E-17 | 376 | | 650 | | |  | | |  | | |
| MF | GO:0016491 | oxidoreductase activity | 3.23E-19 | 2.73E-16 | 202 | | 521 | | |  | | |  | | |
| MF | GO:0000166 | nucleotide binding | 3.40E-18 | 1.91E-15 | 367 | | 614 | | |  | | |  | | |
| MF | GO:1901265 | nucleoside phosphate binding | 3.40E-18 | 1.91E-15 | 367 | | 614 | | |  | | |  | | |
| MF | GO:0003824 | catalytic activity | 3.02E-17 | 1.17E-14 | 1091 | | 1566 | | |  | | |  | | |
| MF | GO:0043168 | anion binding | 1.51E-15 | 4.24E-13 | 371 | | 604 | | |  | | |  | | |
| MF | GO:0016787 | hydrolase activity | 6.99E-15 | 1.77E-12 | 543 | | 712 | | |  | | |  | | |
| MF | GO:0016614 | oxidoreductase activity, acting on CH-OH group of donors | 6.05E-14 | 1.06E-11 | 26 | | 123 | | |  | | |  | | |
| MF | GO:0097159 | organic cyclic compound binding | 6.64E-14 | 1.08E-11 | 729 | | 1154 | | |  | | |  | | |
| MF | GO:1901363 | heterocyclic compound binding | 6.64E-14 | 1.08E-11 | 729 | | 1154 | | |  | | |  | | |
| MF | GO:0003735 | structural constituent of ribosome | 2.16E-13 | 3.41E-11 | 35 | | 170 | | |  | | |  | | |
| MF | GO:0016616 | oxidoreductase activity, acting on the CH-OH group of donors, NAD or NADP as acceptor | 1.00E-12 | 1.33E-10 | 24 | | 111 | | |  | | |  | | |
| MF | GO:0016627 | oxidoreductase activity, acting on the CH-CH group of donors | 7.63E-12 | 7.42E-10 | 22 | | 90 | | |  | | |  | | |
| MF | GO:0050660 | flavin adenine dinucleotide binding | 2.02E-11 | 1.90E-09 | 13 | | 98 | | |  | | |  | | |
| MF | GO:0005215 | transporter activity | 9.43E-11 | 8.08E-09 | 238 | | 462 | | |  | | |  | | |
| MF | GO:0001071 | nucleic acid binding transcription factor activity | 3.20E-09 | 2.18E-07 | 118 | | 263 | | |  | | |  | | |
| MF | GO:0003700 | transcription factor activity, sequence-specific DNA binding | 3.20E-09 | 2.18E-07 | 118 | | 263 | | |  | | |  | | |
| MF | GO:0051287 | NAD binding | 6.0237E-09 | 3.8077E-07 | 13 | | 90 | | |  | | |  | | |
|  |  |  | | | |  | |  | | |  | | |  |  |
|  |  |  | | | |  | |  | | |  | | |  |  |
|  |  |  | | | |  | |  | | |  | | |  |  |
| **Table S3C. GO analysis of DEGs in the muscle between MS vs ML (TOP20).** | | | | | | | | | | |  | | |  |  |
| Category | GOID | Description | pvalue | padj | Up Gene Count | | Down Gene Count | |  | | |  | | | |
| MF | GO:0005044 | scavenger receptor activity | 8.2436E-07 | 0.0020844 | 0 | | 8 | |  | | |  | | | |
| MF | GO:0038024 | cargo receptor activity | 8.2436E-07 | 0.0020844 | 0 | | 8 | |  | | |  | | | |
|  |  |  | | | |  | |  | | |  | | |  |  |
