# Supplementary material for: Starvation Affects the Muscular Morphology, Antioxidant Enzyme Activity, Expression of Lipid Metabolism-Related Genes, and Transcriptomic Profile of Javelin Goby (Synechogobius hasta)
Source: Aquac Nutr. 2022 Dec 30;2022:7057571. doi: 10.1155/2022/7057571 (PMC9973160; doi:10.1155/2022/7057571)
Supplement: Supplementary materials — Table S1: statistics of S. hasta transcriptome data in this study. Table S2: statistics of DEGs in this study. Table S3: GO analysis of DEGs in this study. Table S4: KEGG pathway analysis of DEGs in this study. Table S5: information on 12 representative DEGs determined by RNA-seq. Figure S1: Venn diagram of annotation results in Synechogobius hasta against five common databases. Figure S2: GO enrichment analysis of DEGs in the muscle tissues of Synechogobius hasta. Figure S3: KEGG pathway enrichment analysis of upregulated DEGs and downregulated DEGs in the muscle tissues of Synechogobius hasta. Figure S4: effect of starvation on the expression of selected DEGs in the muscle tissues of Synechogobius hasta. Supplementary method. Method S1: cDNA library construction and sequencing. [file 7057571.f1.zip › 7057571.f1/TABLE S4 KEGG pathway analysis of DEGs in this study.doc]

| **TABLE S4 KEGG pathway analysis of DEGs in this study.** | | | | |  | |  | |
| --- | --- | --- | --- | --- | --- | --- | --- | --- |
|  |  | |  | |  | |  | |
| **Table S4A. KEGG pathway analysis of DEGs in the muscle between MS vs MC.** | | | | | | | | |
|  |  | |  | |  | |  | |
| Pathway ID | Description | P-Value | | Corrected P | | Unigene Count | |  |
| ko04530 | Tight junction | 1.72E-05 | | 0.004261583 | | 35 | |  |
| ko05010 | Alzheimer's disease | 0.000610454 | | 0.075696306 | | 32 | |  |
| ko04260 | Cardiac muscle contraction | 0.004271983 | | 0.287177691 | | 18 | |  |
| ko04932 | Non-alcoholic fatty liver disease (NAFLD) | 0.004631898 | | 0.287177691 | | 21 | |  |
| ko00020 | Citrate cycle (TCA cycle) | 0.0078555 | | 0.38963278 | | 12 | |  |
| ko00190 | Oxidative phosphorylation | 0.011537516 | | 0.476884007 | | 19 | |  |
| ko04261 | Adrenergic signaling in cardiomyocytes | 0.018053095 | | 0.502345956 | | 20 | |  |
| ko05012 | Parkinson's disease | 0.018053095 | | 0.502345956 | | 20 | |  |
| ko00500 | Starch and sucrose metabolism | 0.018230297 | | 0.502345956 | | 9 | |  |
| ko00620 | Pyruvate metabolism | 0.021327725 | | 0.528927574 | | 14 | |  |
| ko00860 | Porphyrin and chlorophyll metabolism | 0.036772698 | | 0.829057199 | | 7 | |  |
| ko04022 | cGMP-PKG signaling pathway | 0.044418213 | | 0.849382903 | | 22 | |  |
| ko04931 | Insulin resistance | 0.047113238 | | 0.849382903 | | 13 | |  |
| ko00010 | Glycolysis / Gluconeogenesis | 0.047949035 | | 0.849382903 | | 15 | |  |
| ko00280 | Valine, leucine and isoleucine degradation | 0.061352639 | | 0.941648498 | | 14 | |  |
| ko05220 | Chronic myeloid leukemia | 0.062756218 | | 0.941648498 | | 8 | |  |
| ko04910 | Insulin signaling pathway | 0.064548486 | | 0.941648498 | | 15 | |  |
| ko04020 | Calcium signaling pathway | 0.126825481 | | 0.994295341 | | 19 | |  |
| ko04013 | MAPK signaling pathway - fly | 0.132949025 | | 0.994295341 | | 9 | |  |
| ko04211 | Longevity regulating pathway | 0.134234899 | | 0.994295341 | | 11 | |  |
| ko04141 | Protein processing in endoplasmic reticulum | 0.139158829 | | 0.994295341 | | 13 | |  |
| ko03010 | Ribosome | 0.143072633 | | 0.994295341 | | 27 | |  |
| ko05016 | Huntington's disease | 0.14706099 | | 0.994295341 | | 19 | |  |
| ko03060 | Protein export | 0.148882629 | | 0.994295341 | | 3 | |  |
| ko00130 | Ubiquinone and other terpenoid-quinone biosynthesis | 0.157255355 | | 0.994295341 | | 2 | |  |
| ko04914 | Progesterone-mediated oocyte maturation | 0.162332823 | | 0.994295341 | | 8 | |  |
| ko05212 | Pancreatic cancer | 0.165942145 | | 0.994295341 | | 7 | |  |
| ko00040 | Pentose and glucuronate interconversions | 0.18214832 | | 0.994295341 | | 5 | |  |
| ko04972 | Pancreatic secretion | 0.188208806 | | 0.994295341 | | 13 | |  |
| ko04810 | Regulation of actin cytoskeleton | 0.190250262 | | 0.994295341 | | 17 | |  |
| ko00340 | Histidine metabolism | 0.196238959 | | 0.994295341 | | 5 | |  |
| ko05410 | Hypertrophic cardiomyopathy (HCM) | 0.214587274 | | 0.994295341 | | 10 | |  |
| ko05014 | Amyotrophic lateral sclerosis (ALS) | 0.218259835 | | 0.994295341 | | 6 | |  |
| ko00260 | Glycine, serine and threonine metabolism | 0.224040495 | | 0.994295341 | | 10 | |  |
| ko04110 | Cell cycle | 0.224076902 | | 0.994295341 | | 8 | |  |
| ko00030 | Pentose phosphate pathway | 0.225470083 | | 0.994295341 | | 5 | |  |
| ko00561 | Glycerolipid metabolism | 0.234244597 | | 0.994295341 | | 7 | |  |
| ko04114 | Oocyte meiosis | 0.234788632 | | 0.994295341 | | 9 | |  |
| ko00630 | Glyoxylate and dicarboxylate metabolism | 0.235122706 | | 0.994295341 | | 8 | |  |
| ko00520 | Amino sugar and nucleotide sugar metabolism | 0.244895439 | | 0.994295341 | | 6 | |  |
| ko05340 | Primary immunodeficiency | 0.253787891 | | 0.994295341 | | 3 | |  |
| ko04930 | Type II diabetes mellitus | 0.271440544 | | 0.994295341 | | 5 | |  |
| ko03015 | mRNA surveillance pathway | 0.272406894 | | 0.994295341 | | 6 | |  |
| ko00760 | Nicotinate and nicotinamide metabolism | 0.276002314 | | 0.994295341 | | 3 | |  |
| ko04120 | Ubiquitin mediated proteolysis | 0.277401708 | | 0.994295341 | | 9 | |  |
| ko04722 | Neurotrophin signaling pathway | 0.277401708 | | 0.994295341 | | 9 | |  |
| ko00534 | Glycosaminoglycan biosynthesis - HS/Hep | 0.277762065 | | 0.994295341 | | 2 | |  |
| ko04212 | Longevity regulating pathway - worm | 0.281009835 | | 0.994295341 | | 8 | |  |
| ko00640 | Propanoate metabolism | 0.284141119 | | 0.994295341 | | 7 | |  |
| ko04920 | Adipocytokine signaling pathway | 0.286433019 | | 0.994295341 | | 6 | |  |
| ko04350 | TGF-beta signaling pathway | 0.286433019 | | 0.994295341 | | 6 | |  |
| ko04940 | Type I diabetes mellitus | 0.298395382 | | 0.994295341 | | 3 | |  |
| ko00053 | Ascorbate and aldarate metabolism | 0.303362708 | | 0.994295341 | | 4 | |  |
| ko04621 | NOD-like receptor signaling pathway | 0.303362708 | | 0.994295341 | | 4 | |  |
| ko04150 | mTOR signaling pathway | 0.314910007 | | 0.994295341 | | 6 | |  |
| ko04728 | Dopaminergic synapse | 0.321982701 | | 0.994295341 | | 9 | |  |
| ko05215 | Prostate cancer | 0.323086392 | | 0.994295341 | | 7 | |  |
| ko05414 | Dilated cardiomyopathy (DCM) | 0.3259122 | | 0.994295341 | | 10 | |  |
| ko05210 | Colorectal cancer | 0.329314965 | | 0.994295341 | | 6 | |  |
| ko03430 | Mismatch repair | 0.338458898 | | 0.994295341 | | 2 | |  |
| ko04146 | Peroxisome | 0.35348737 | | 0.994295341 | | 8 | |  |
| ko00220 | Arginine biosynthesis | 0.359111602 | | 0.994295341 | | 4 | |  |
| ko05223 | Non-small cell lung cancer | 0.359111602 | | 0.994295341 | | 4 | |  |
| ko00770 | Pantothenate and CoA biosynthesis | 0.368252931 | | 0.994295341 | | 2 | |  |
| ko00052 | Galactose metabolism | 0.388091936 | | 0.994295341 | | 3 | |  |
| ko04024 | cAMP signaling pathway | 0.388788198 | | 0.994295341 | | 14 | |  |
| ko05034 | Alcoholism | 0.389525158 | | 0.994295341 | | 7 | |  |
| ko00310 | Lysine degradation | 0.389525158 | | 0.994295341 | | 7 | |  |
| ko05169 | Epstein-Barr virus infection | 0.390751218 | | 0.994295341 | | 11 | |  |
| ko00524 | Butirosin and neomycin biosynthesis | 0.392413767 | | 0.994295341 | | 1 | |  |
| ko05200 | Pathways in cancer | 0.393239405 | | 0.994295341 | | 23 | |  |
| ko04151 | PI3K-Akt signaling pathway | 0.396253397 | | 0.994295341 | | 21 | |  |
| ko03008 | Ribosome biogenesis in eukaryotes | 0.396285068 | | 0.994295341 | | 4 | |  |
| ko04664 | Fc epsilon RI signaling pathway | 0.396285068 | | 0.994295341 | | 4 | |  |
| ko00910 | Nitrogen metabolism | 0.397502305 | | 0.994295341 | | 2 | |  |
| ko04666 | Fc gamma R-mediated phagocytosis | 0.402119669 | | 0.994295341 | | 6 | |  |
| ko05221 | Acute myeloid leukemia | 0.414751553 | | 0.994295341 | | 4 | |  |
| ko05146 | Amoebiasis | 0.416689949 | | 0.994295341 | | 6 | |  |
| ko03410 | Base excision repair | 0.426104674 | | 0.994295341 | | 2 | |  |
| ko00120 | Primary bile acid biosynthesis | 0.426104674 | | 0.994295341 | | 2 | |  |
| ko00531 | Glycosaminoglycan degradation | 0.426104674 | | 0.994295341 | | 2 | |  |
| ko05231 | Choline metabolism in cancer | 0.431217456 | | 0.994295341 | | 6 | |  |
| ko00970 | Aminoacyl-tRNA biosynthesis | 0.431217456 | | 0.994295341 | | 6 | |  |
| ko05142 | Chagas disease (American trypanosomiasis) | 0.440145686 | | 0.994295341 | | 8 | |  |
| ko00780 | Biotin metabolism | 0.440840936 | | 0.994295341 | | 1 | |  |
| ko03018 | RNA degradation | 0.445683981 | | 0.994295341 | | 6 | |  |
| ko04012 | ErbB signaling pathway | 0.448516987 | | 0.994295341 | | 5 | |  |
| ko05230 | Central carbon metabolism in cancer | 0.448516987 | | 0.994295341 | | 5 | |  |
| ko04152 | AMPK signaling pathway | 0.44907335 | | 0.994295341 | | 9 | |  |
| ko04725 | Cholinergic synapse | 0.44907335 | | 0.994295341 | | 9 | |  |
| ko05203 | Viral carcinogenesis | 0.45626855 | | 0.994295341 | | 13 | |  |
| ko04620 | Toll-like receptor signaling pathway | 0.464407572 | | 0.994295341 | | 5 | |  |
| ko04917 | Prolactin signaling pathway | 0.464407572 | | 0.994295341 | | 5 | |  |
| ko04071 | Sphingolipid signaling pathway | 0.464756317 | | 0.994295341 | | 8 | |  |
| ko04670 | Leukocyte transendothelial migration | 0.472122913 | | 0.994295341 | | 9 | |  |
| ko04068 | FoxO signaling pathway | 0.472122913 | | 0.994295341 | | 9 | |  |
| ko00330 | Arginine and proline metabolism | 0.472122913 | | 0.994295341 | | 9 | |  |
| ko00071 | Fatty acid degradation | 0.474365246 | | 0.994295341 | | 6 | |  |
| ko04973 | Carbohydrate digestion and absorption | 0.474758128 | | 0.994295341 | | 3 | |  |
| ko04330 | Notch signaling pathway | 0.474758128 | | 0.994295341 | | 3 | |  |
| ko04921 | Oxytocin signaling pathway | 0.479585586 | | 0.994295341 | | 12 | |  |
| ko03030 | DNA replication | 0.481055245 | | 0.994295341 | | 2 | |  |
| ko05222 | Small cell lung cancer | 0.495746876 | | 0.994295341 | | 5 | |  |
| ko04668 | TNF signaling pathway | 0.502604389 | | 0.994295341 | | 6 | |  |
| ko00480 | Glutathione metabolism | 0.504522005 | | 0.994295341 | | 4 | |  |
| ko04370 | VEGF signaling pathway | 0.504522005 | | 0.994295341 | | 4 | |  |
| ko04960 | Aldosterone-regulated sodium reabsorption | 0.507287777 | | 0.994295341 | | 2 | |  |
| ko04710 | Circadian rhythm | 0.515940912 | | 0.994295341 | | 3 | |  |
| ko05202 | Transcriptional misregulation in cancer | 0.521083828 | | 0.994295341 | | 7 | |  |
| ko04750 | Inflammatory mediator regulation of TRP channels | 0.521083828 | | 0.994295341 | | 7 | |  |
| ko05031 | Amphetamine addiction | 0.521765325 | | 0.994295341 | | 4 | |  |
| ko04662 | B cell receptor signaling pathway | 0.521765325 | | 0.994295341 | | 4 | |  |
| ko05211 | Renal cell carcinoma | 0.521765325 | | 0.994295341 | | 4 | |  |
| ko05161 | Hepatitis B | 0.525092385 | | 0.994295341 | | 8 | |  |
| ko04971 | Gastric acid secretion | 0.526371459 | | 0.994295341 | | 5 | |  |
| ko04140 | Regulation of autophagy | 0.526430116 | | 0.994295341 | | 1 | |  |
| ko04711 | Circadian rhythm - fly | 0.526430116 | | 0.994295341 | | 1 | |  |
| ko04213 | Longevity regulating pathway - multiple species | 0.530285712 | | 0.994295341 | | 6 | |  |
| ko05030 | Cocaine addiction | 0.535870118 | | 0.994295341 | | 3 | |  |
| ko03460 | Fanconi anemia pathway | 0.535870118 | | 0.994295341 | | 3 | |  |
| ko02010 | ABC transporters | 0.538716994 | | 0.994295341 | | 4 | |  |
| ko04933 | AGE-RAGE signaling pathway in diabetic complications | 0.543884835 | | 0.994295341 | | 6 | |  |
| ko00564 | Glycerophospholipid metabolism | 0.543884835 | | 0.994295341 | | 6 | |  |
| ko04115 | p53 signaling pathway | 0.555358228 | | 0.994295341 | | 4 | |  |
| ko00250 | Alanine, aspartate and glutamate metabolism | 0.555358228 | | 0.994295341 | | 4 | |  |
| ko04660 | T cell receptor signaling pathway | 0.556149778 | | 0.994295341 | | 5 | |  |
| ko03420 | Nucleotide excision repair | 0.557079237 | | 0.994295341 | | 2 | |  |
| ko04723 | Retrograde endocannabinoid signaling | 0.557307415 | | 0.994295341 | | 6 | |  |
| ko05332 | Graft-versus-host disease | 0.564181824 | | 0.994295341 | | 1 | |  |
| ko05145 | Toxoplasmosis | 0.570884835 | | 0.994295341 | | 7 | |  |
| ko04062 | Chemokine signaling pathway | 0.582812473 | | 0.994295341 | | 8 | |  |
| ko04520 | Adherens junction | 0.583581564 | | 0.994295341 | | 6 | |  |
| ko04391 | Hippo signaling pathway - fly | 0.584971658 | | 0.994295341 | | 5 | |  |
| ko04510 | Focal adhesion | 0.586297883 | | 0.994295341 | | 14 | |  |
| ko05213 | Endometrial cancer | 0.592722634 | | 0.994295341 | | 3 | |  |
| ko04612 | Antigen processing and presentation | 0.603263866 | | 0.994295341 | | 4 | |  |
| ko04380 | Osteoclast differentiation | 0.609033809 | | 0.994295341 | | 6 | |  |
| ko04215 | Apoptosis - multiple species | 0.624830665 | | 0.994295341 | | 2 | |  |
| ko00983 | Drug metabolism - other enzymes | 0.624830665 | | 0.994295341 | | 2 | |  |
| ko00380 | Tryptophan metabolism | 0.626220259 | | 0.994295341 | | 5 | |  |
| ko05330 | Allograft rejection | 0.630902011 | | 0.994295341 | | 1 | |  |
| ko04014 | Ras signaling pathway | 0.641536588 | | 0.994295341 | | 10 | |  |
| ko04390 | Hippo signaling pathway | 0.647357637 | | 0.994295341 | | 8 | |  |
| ko00410 | beta-Alanine metabolism | 0.647899476 | | 0.994295341 | | 4 | |  |
| ko05100 | Bacterial invasion of epithelial cells | 0.652300477 | | 0.994295341 | | 5 | |  |
| ko00061 | Fatty acid biosynthesis | 0.66033034 | | 0.994295341 | | 1 | |  |
| ko00072 | Synthesis and degradation of ketone bodies | 0.66033034 | | 0.994295341 | | 1 | |  |
| ko03320 | PPAR signaling pathway | 0.664896849 | | 0.994295341 | | 5 | |  |
| ko05131 | Shigellosis | 0.675739276 | | 0.994295341 | | 4 | |  |
| ko04630 | Jak-STAT signaling pathway | 0.675739276 | | 0.994295341 | | 4 | |  |
| ko05160 | Hepatitis C | 0.679908399 | | 0.994295341 | | 6 | |  |
| ko04672 | Intestinal immune network for IgA production | 0.687413821 | | 0.994295341 | | 1 | |  |
| ko05033 | Nicotine addiction | 0.687413821 | | 0.994295341 | | 1 | |  |
| ko00532 | Glycosaminoglycan biosynthesis - CS/DS | 0.687413821 | | 0.994295341 | | 1 | |  |
| ko05320 | Autoimmune thyroid disease | 0.687413821 | | 0.994295341 | | 1 | |  |
| ko04923 | Regulation of lipolysis in adipocytes | 0.689072046 | | 0.994295341 | | 4 | |  |
| ko04922 | Glucagon signaling pathway | 0.690870624 | | 0.994295341 | | 6 | |  |
| ko05168 | Herpes simplex infection | 0.690952216 | | 0.994295341 | | 9 | |  |
| ko00562 | Inositol phosphate metabolism | 0.702011305 | | 0.994295341 | | 4 | |  |
| ko04070 | Phosphatidylinositol signaling system | 0.712241023 | | 0.994295341 | | 5 | |  |
| ko00900 | Terpenoid backbone biosynthesis | 0.712339172 | | 0.994295341 | | 1 | |  |
| ko04320 | Dorso-ventral axis formation | 0.712339172 | | 0.994295341 | | 1 | |  |
| ko04915 | Estrogen signaling pathway | 0.713072356 | | 0.994295341 | | 7 | |  |
| ko00670 | One carbon pool by folate | 0.719428058 | | 0.994295341 | | 2 | |  |
| ko05162 | Measles | 0.72330771 | | 0.994295341 | | 5 | |  |
| ko05416 | Viral myocarditis | 0.734408368 | | 0.994295341 | | 3 | |  |
| ko05120 | Epithelial cell signaling in Helicobacter pylori infection | 0.734408368 | | 0.994295341 | | 3 | |  |
| ko03440 | Homologous recombination | 0.735278255 | | 0.994295341 | | 1 | |  |
| ko04010 | MAPK signaling pathway | 0.744887537 | | 0.994295341 | | 11 | |  |
| ko04730 | Long-term depression | 0.74745446 | | 0.994295341 | | 3 | |  |
| ko03020 | RNA polymerase | 0.756389252 | | 0.994295341 | | 1 | |  |
| ko00830 | Retinol metabolism | 0.76594067 | | 0.994295341 | | 2 | |  |
| ko05218 | Melanoma | 0.779884339 | | 0.994295341 | | 2 | |  |
| ko00600 | Sphingolipid metabolism | 0.779884339 | | 0.994295341 | | 2 | |  |
| ko04066 | HIF-1 signaling pathway | 0.790755536 | | 0.994295341 | | 7 | |  |
| ko05132 | Salmonella infection | 0.791563231 | | 0.994295341 | | 4 | |  |
| ko04214 | Apoptosis - fly | 0.793093816 | | 0.994295341 | | 2 | |  |
| ko04720 | Long-term potentiation | 0.794560955 | | 0.994295341 | | 3 | |  |
| ko04072 | Phospholipase D signaling pathway | 0.794754505 | | 0.994295341 | | 6 | |  |
| ko04713 | Circadian entrainment | 0.800873077 | | 0.994295341 | | 5 | |  |
| ko00450 | Selenocompound metabolism | 0.810152644 | | 0.994295341 | | 1 | |  |
| ko05214 | Glioma | 0.817423862 | | 0.994295341 | | 2 | |  |
| ko00230 | Purine metabolism | 0.81750657 | | 0.994295341 | | 10 | |  |
| ko04144 | Endocytosis | 0.821827057 | | 0.994295341 | | 11 | |  |
| ko05133 | Pertussis | 0.835641162 | | 0.994295341 | | 4 | |  |
| ko00565 | Ether lipid metabolism | 0.839231989 | | 0.994295341 | | 1 | |  |
| ko05216 | Thyroid cancer | 0.839231989 | | 0.994295341 | | 1 | |  |
| ko03050 | Proteasome | 0.839231989 | | 0.994295341 | | 1 | |  |
| ko04724 | Glutamatergic synapse | 0.84693197 | | 0.994295341 | | 5 | |  |
| ko04650 | Natural killer cell mediated cytotoxicity | 0.849122608 | | 0.994295341 | | 2 | |  |
| ko04064 | NF-kappa B signaling pathway | 0.851285081 | | 0.994295341 | | 3 | |  |
| ko04341 | Hedgehog signaling pathway - fly | 0.852057114 | | 0.994295341 | | 1 | |  |
| ko00270 | Cysteine and methionine metabolism | 0.858143371 | | 0.994295341 | | 4 | |  |
| ko04925 | Aldosterone synthesis and secretion | 0.858143371 | | 0.994295341 | | 4 | |  |
| ko04912 | GnRH signaling pathway | 0.858143371 | | 0.994295341 | | 4 | |  |
| ko05164 | Influenza A | 0.862807687 | | 0.994295341 | | 7 | |  |
| ko04611 | Platelet activation | 0.862924974 | | 0.994295341 | | 8 | |  |
| ko00140 | Steroid hormone biosynthesis | 0.863859773 | | 0.994295341 | | 1 | |  |
| ko04623 | Cytosolic DNA-sensing pathway | 0.863859773 | | 0.994295341 | | 1 | |  |
| ko04727 | GABAergic synapse | 0.86689899 | | 0.994295341 | | 3 | |  |
| ko05032 | Morphine addiction | 0.86689899 | | 0.994295341 | | 3 | |  |
| ko04622 | RIG-I-like receptor signaling pathway | 0.867381673 | | 0.994295341 | | 2 | |  |
| ko05321 | Inflammatory bowel disease (IBD) | 0.87472143 | | 0.994295341 | | 1 | |  |
| ko04962 | Vasopressin-regulated water reabsorption | 0.875729371 | | 0.994295341 | | 2 | |  |
| ko04080 | Neuroactive ligand-receptor interaction | 0.87849712 | | 0.994295341 | | 7 | |  |
| ko04970 | Salivary secretion | 0.88104897 | | 0.994295341 | | 3 | |  |
| ko05323 | Rheumatoid arthritis | 0.883589899 | | 0.994295341 | | 2 | |  |
| ko00590 | Arachidonic acid metabolism | 0.88471706 | | 0.994295341 | | 1 | |  |
| ko04924 | Renin secretion | 0.887609 | | 0.994295341 | | 3 | |  |
| ko04270 | Vascular smooth muscle contraction | 0.889146654 | | 0.994295341 | | 5 | |  |
| ko04512 | ECM-receptor interaction | 0.893843534 | | 0.994295341 | | 3 | |  |
| ko00510 | N-Glycan biosynthesis | 0.893915672 | | 0.994295341 | | 1 | |  |
| ko00350 | Tyrosine metabolism | 0.893915672 | | 0.994295341 | | 1 | |  |
| ko00240 | Pyrimidine metabolism | 0.90062531 | | 0.994295341 | | 4 | |  |
| ko05110 | Vibrio cholerae infection | 0.904490068 | | 0.994295341 | | 2 | |  |
| ko04742 | Taste transduction | 0.910170825 | | 0.994295341 | | 1 | |  |
| ko00051 | Fructose and mannose metabolism | 0.917339619 | | 0.994295341 | | 1 | |  |
| ko04918 | Thyroid hormone synthesis | 0.920582577 | | 0.994295341 | | 3 | |  |
| ko05152 | Tuberculosis | 0.92378404 | | 0.994295341 | | 7 | |  |
| ko03040 | Spliceosome | 0.925129429 | | 0.994295341 | | 3 | |  |
| ko00650 | Butanoate metabolism | 0.930007551 | | 0.994295341 | | 1 | |  |
| ko05205 | Proteoglycans in cancer | 0.930577477 | | 0.994295341 | | 10 | |  |
| ko04919 | Thyroid hormone signaling pathway | 0.931459409 | | 0.994295341 | | 4 | |  |
| ko04976 | Bile secretion | 0.931707938 | | 0.994295341 | | 2 | |  |
| ko03013 | RNA transport | 0.931889758 | | 0.994295341 | | 5 | |  |
| ko04916 | Melanogenesis | 0.937374188 | | 0.994295341 | | 3 | |  |
| ko05144 | Malaria | 0.940735215 | | 0.994295341 | | 1 | |  |
| ko04740 | Olfactory transduction | 0.945466123 | | 0.994295341 | | 1 | |  |
| ko04913 | Ovarian steroidogenesis | 0.945466123 | | 0.994295341 | | 1 | |  |
| ko04360 | Axon guidance | 0.946737242 | | 0.994295341 | | 7 | |  |
| ko05412 | Arrhythmogenic right ventricular cardiomyopathy (ARVC) | 0.948013511 | | 0.994295341 | | 2 | |  |
| ko05219 | Bladder cancer | 0.949819618 | | 0.994295341 | | 1 | |  |
| ko05206 | MicroRNAs in cancer | 0.954761138 | | 0.994295341 | | 5 | |  |
| ko04015 | Rap1 signaling pathway | 0.957062493 | | 0.994295341 | | 8 | |  |
| ko04210 | Apoptosis | 0.957064485 | | 0.994295341 | | 5 | |  |
| ko05134 | Legionellosis | 0.962787742 | | 0.994295341 | | 4 | |  |
| ko04726 | Serotonergic synapse | 0.968010716 | | 0.994295341 | | 2 | |  |
| ko04060 | Cytokine-cytokine receptor interaction | 0.970177226 | | 0.994295341 | | 2 | |  |
| ko04145 | Phagosome | 0.970408571 | | 0.994295341 | | 5 | |  |
| ko04550 | Signaling pathways regulating pluripotency of stem cells | 0.975207925 | | 0.994295341 | | 3 | |  |
| ko04911 | Insulin secretion | 0.975860455 | | 0.994295341 | | 2 | |  |
| ko04514 | Cell adhesion molecules (CAMs) | 0.979051162 | | 0.994295341 | | 2 | |  |
| ko04540 | Gap junction | 0.98048927 | | 0.994295341 | | 2 | |  |
| ko04978 | Mineral absorption | 0.989681296 | | 0.994295341 | | 1 | |  |
| ko05322 | Systemic lupus erythematosus | 0.989681296 | | 0.994295341 | | 1 | |  |
| ko04974 | Protein digestion and absorption | 0.990493428 | | 0.994295341 | | 2 | |  |
| ko05140 | Leishmaniasis | 0.992605352 | | 0.994295341 | | 1 | |  |
| ko04142 | Lysosome | 0.994295341 | | 0.994295341 | | 2 | |  |
|  |  | |  | |  | |  | |
|  |  | |  | |  | |  | |
|  |  | |  | |  | |  | |
| **Table S4B. KEGG pathway analysis of DEGs in the muscle between ML vs MC.** | | | | | | | | |
|  |  | |  | |  | |  | |
| Pathway ID | Description | P-Value | | Corrected P | | Unigene Count | |  |
| ko04151 | PI3K-Akt signaling pathway | 0.01237764 | | 0.998067863 | | 67 | |  |
| ko00860 | Porphyrin and chlorophyll metabolism | 0.016041753 | | 0.998067863 | | 15 | |  |
| ko04974 | Protein digestion and absorption | 0.028575472 | | 0.998067863 | | 27 | |  |
| ko04932 | Non-alcoholic fatty liver disease (NAFLD) | 0.032143315 | | 0.998067863 | | 38 | |  |
| ko04530 | Tight junction | 0.039456189 | | 0.998067863 | | 50 | |  |
| ko04512 | ECM-receptor interaction | 0.046636277 | | 0.998067863 | | 21 | |  |
| ko04013 | MAPK signaling pathway - fly | 0.048169265 | | 0.998067863 | | 22 | |  |
| ko04391 | Hippo signaling pathway - fly | 0.056974936 | | 0.998067863 | | 20 | |  |
| ko00760 | Nicotinate and nicotinamide metabolism | 0.057966128 | | 0.998067863 | | 9 | |  |
| ko04933 | AGE-RAGE signaling pathway in diabetic complications | 0.066619282 | | 0.998067863 | | 22 | |  |
| ko04722 | Neurotrophin signaling pathway | 0.069520466 | | 0.998067863 | | 25 | |  |
| ko05220 | Chronic myeloid leukemia | 0.071236403 | | 0.998067863 | | 16 | |  |
| ko00040 | Pentose and glucuronate interconversions | 0.088737128 | | 0.998067863 | | 12 | |  |
| ko05164 | Influenza A | 0.090585567 | | 0.998067863 | | 33 | |  |
| ko05142 | Chagas disease (American trypanosomiasis) | 0.091022129 | | 0.998067863 | | 25 | |  |
| ko03010 | Ribosome | 0.101668945 | | 0.998067863 | | 64 | |  |
| ko05010 | Alzheimer's disease | 0.101854441 | | 0.998067863 | | 50 | |  |
| ko05222 | Small cell lung cancer | 0.102780873 | | 0.998067863 | | 17 | |  |
| ko04660 | T cell receptor signaling pathway | 0.104192941 | | 0.998067863 | | 18 | |  |
| ko04390 | Hippo signaling pathway | 0.106533751 | | 0.998067863 | | 29 | |  |
| ko04260 | Cardiac muscle contraction | 0.10691183 | | 0.998067863 | | 28 | |  |
| ko04931 | Insulin resistance | 0.107556594 | | 0.998067863 | | 25 | |  |
| ko00500 | Starch and sucrose metabolism | 0.1082102 | | 0.998067863 | | 14 | |  |
| ko00130 | Ubiquinone and other terpenoid-quinone biosynthesis | 0.114498187 | | 0.998067863 | | 4 | |  |
| ko04914 | Progesterone-mediated oocyte maturation | 0.126378801 | | 0.998067863 | | 18 | |  |
| ko05203 | Viral carcinogenesis | 0.129364719 | | 0.998067863 | | 38 | |  |
| ko04612 | Antigen processing and presentation | 0.136453851 | | 0.998067863 | | 15 | |  |
| ko00190 | Oxidative phosphorylation | 0.137416022 | | 0.998067863 | | 32 | |  |
| ko00020 | Citrate cycle (TCA cycle) | 0.151223609 | | 0.998067863 | | 17 | |  |
| ko00510 | N-Glycan biosynthesis | 0.154128112 | | 0.998067863 | | 9 | |  |
| ko05145 | Toxoplasmosis | 0.159332726 | | 0.998067863 | | 23 | |  |
| ko05012 | Parkinson's disease | 0.159350922 | | 0.998067863 | | 35 | |  |
| ko05223 | Non-small cell lung cancer | 0.161204208 | | 0.998067863 | | 11 | |  |
| ko04920 | Adipocytokine signaling pathway | 0.165437108 | | 0.998067863 | | 15 | |  |
| ko05212 | Pancreatic cancer | 0.165437108 | | 0.998067863 | | 15 | |  |
| ko05169 | Epstein-Barr virus infection | 0.169242931 | | 0.998067863 | | 30 | |  |
| ko04141 | Protein processing in endoplasmic reticulum | 0.173286107 | | 0.998067863 | | 28 | |  |
| ko05146 | Amoebiasis | 0.179428524 | | 0.998067863 | | 17 | |  |
| ko04917 | Prolactin signaling pathway | 0.181030693 | | 0.998067863 | | 15 | |  |
| ko05332 | Graft-versus-host disease | 0.186217697 | | 0.998067863 | | 4 | |  |
| ko05321 | Inflammatory bowel disease (IBD) | 0.194301455 | | 0.998067863 | | 8 | |  |
| ko04150 | mTOR signaling pathway | 0.197317349 | | 0.998067863 | | 15 | |  |
| ko05200 | Pathways in cancer | 0.212213928 | | 0.998067863 | | 60 | |  |
| ko04730 | Long-term depression | 0.217823007 | | 0.998067863 | | 13 | |  |
| ko05160 | Hepatitis C | 0.229279278 | | 0.998067863 | | 21 | |  |
| ko04370 | VEGF signaling pathway | 0.239489416 | | 0.998067863 | | 12 | |  |
| ko04728 | Dopaminergic synapse | 0.240354654 | | 0.998067863 | | 22 | |  |
| ko04392 | Hippo signaling pathway - multiple species | 0.244836272 | | 0.998067863 | | 7 | |  |
| ko04261 | Adrenergic signaling in cardiomyocytes | 0.249056309 | | 0.998067863 | | 33 | |  |
| ko04071 | Sphingolipid signaling pathway | 0.255096811 | | 0.998067863 | | 22 | |  |
| ko04514 | Cell adhesion molecules (CAMs) | 0.255949272 | | 0.998067863 | | 18 | |  |
| ko04380 | Osteoclast differentiation | 0.268238634 | | 0.998067863 | | 19 | |  |
| ko05330 | Allograft rejection | 0.268380338 | | 0.998067863 | | 4 | |  |
| ko04664 | Fc epsilon RI signaling pathway | 0.291167559 | | 0.998067863 | | 10 | |  |
| ko04630 | Jak-STAT signaling pathway | 0.292949343 | | 0.998067863 | | 14 | |  |
| ko05217 | Basal cell carcinoma | 0.295752166 | | 0.998067863 | | 9 | |  |
| ko05030 | Cocaine addiction | 0.295752166 | | 0.998067863 | | 9 | |  |
| ko04115 | p53 signaling pathway | 0.304024349 | | 0.998067863 | | 12 | |  |
| ko05410 | Hypertrophic cardiomyopathy (HCM) | 0.307083972 | | 0.998067863 | | 21 | |  |
| ko04711 | Circadian rhythm - fly | 0.307481559 | | 0.998067863 | | 3 | |  |
| ko00531 | Glycosaminoglycan degradation | 0.310929478 | | 0.998067863 | | 5 | |  |
| ko04978 | Mineral absorption | 0.313350383 | | 0.998067863 | | 14 | |  |
| ko05322 | Systemic lupus erythematosus | 0.313350383 | | 0.998067863 | | 14 | |  |
| ko05144 | Malaria | 0.322161302 | | 0.998067863 | | 9 | |  |
| ko00010 | Glycolysis / Gluconeogenesis | 0.32984923 | | 0.998067863 | | 25 | |  |
| ko04910 | Insulin signaling pathway | 0.338538655 | | 0.998067863 | | 26 | |  |
| ko04620 | Toll-like receptor signaling pathway | 0.341511651 | | 0.998067863 | | 13 | |  |
| ko04940 | Type I diabetes mellitus | 0.342117474 | | 0.998067863 | | 6 | |  |
| ko05216 | Thyroid cancer | 0.342117474 | | 0.998067863 | | 6 | |  |
| ko04915 | Estrogen signaling pathway | 0.342982797 | | 0.998067863 | | 23 | |  |
| ko04062 | Chemokine signaling pathway | 0.342982797 | | 0.998067863 | | 23 | |  |
| ko05202 | Transcriptional misregulation in cancer | 0.354731481 | | 0.998067863 | | 19 | |  |
| ko04672 | Intestinal immune network for IgA production | 0.355380963 | | 0.998067863 | | 4 | |  |
| ko05320 | Autoimmune thyroid disease | 0.355380963 | | 0.998067863 | | 4 | |  |
| ko04662 | B cell receptor signaling pathway | 0.357668933 | | 0.998067863 | | 11 | |  |
| ko04110 | Cell cycle | 0.359352468 | | 0.998067863 | | 16 | |  |
| ko03320 | PPAR signaling pathway | 0.359352468 | | 0.998067863 | | 16 | |  |
| ko04668 | TNF signaling pathway | 0.359352468 | | 0.998067863 | | 16 | |  |
| ko00524 | Butirosin and neomycin biosynthesis | 0.360000613 | | 0.998067863 | | 2 | |  |
| ko04930 | Type II diabetes mellitus | 0.366564495 | | 0.998067863 | | 10 | |  |
| ko03018 | RNA degradation | 0.36763663 | | 0.998067863 | | 15 | |  |
| ko05162 | Measles | 0.37059276 | | 0.998067863 | | 17 | |  |
| ko05120 | Epithelial cell signaling in Helicobacter pylori infection | 0.381978599 | | 0.998067863 | | 11 | |  |
| ko04211 | Longevity regulating pathway | 0.382400451 | | 0.998067863 | | 20 | |  |
| ko00053 | Ascorbate and aldarate metabolism | 0.386534038 | | 0.998067863 | | 8 | |  |
| ko04621 | NOD-like receptor signaling pathway | 0.386534038 | | 0.998067863 | | 8 | |  |
| ko04114 | Oocyte meiosis | 0.399809235 | | 0.998067863 | | 18 | |  |
| ko03008 | Ribosome biogenesis in eukaryotes | 0.403425676 | | 0.998067863 | | 9 | |  |
| ko00030 | Pentose phosphate pathway | 0.403425676 | | 0.998067863 | | 9 | |  |
| ko05014 | Amyotrophic lateral sclerosis (ALS) | 0.406425359 | | 0.998067863 | | 11 | |  |
| ko04510 | Focal adhesion | 0.40836831 | | 0.998067863 | | 38 | |  |
| ko05161 | Hepatitis B | 0.408833387 | | 0.998067863 | | 21 | |  |
| ko04152 | AMPK signaling pathway | 0.417080616 | | 0.998067863 | | 22 | |  |
| ko05414 | Dilated cardiomyopathy (DCM) | 0.417080616 | | 0.998067863 | | 22 | |  |
| ko04012 | ErbB signaling pathway | 0.419059986 | | 0.998067863 | | 12 | |  |
| ko05340 | Primary immunodeficiency | 0.425208043 | | 0.998067863 | | 5 | |  |
| ko00603 | Glycosphingolipid biosynthesis - globo series | 0.428233271 | | 0.998067863 | | 2 | |  |
| ko04360 | Axon guidance | 0.433719876 | | 0.998067863 | | 31 | |  |
| ko04723 | Retrograde endocannabinoid signaling | 0.439189853 | | 0.998067863 | | 16 | |  |
| ko04540 | Gap junction | 0.439189853 | | 0.998067863 | | 16 | |  |
| ko00983 | Drug metabolism - other enzymes | 0.444805177 | | 0.998067863 | | 6 | |  |
| ko00620 | Pyruvate metabolism | 0.445799107 | | 0.998067863 | | 19 | |  |
| ko04611 | Platelet activation | 0.4503304 | | 0.998067863 | | 29 | |  |
| ko04670 | Leukocyte transendothelial migration | 0.451057692 | | 0.998067863 | | 22 | |  |
| ko05205 | Proteoglycans in cancer | 0.452060892 | | 0.998067863 | | 39 | |  |
| ko00561 | Glycerolipid metabolism | 0.452830219 | | 0.998067863 | | 13 | |  |
| ko04916 | Melanogenesis | 0.459233428 | | 0.998067863 | | 16 | |  |
| ko04973 | Carbohydrate digestion and absorption | 0.460499417 | | 0.998067863 | | 7 | |  |
| ko00982 | Drug metabolism - cytochrome P450 | 0.462787803 | | 0.998067863 | | 5 | |  |
| ko00512 | Mucin type O-Glycan biosynthesis | 0.463480448 | | 0.998067863 | | 3 | |  |
| ko00052 | Galactose metabolism | 0.478487806 | | 0.998067863 | | 6 | |  |
| ko04919 | Thyroid hormone signaling pathway | 0.482387832 | | 0.998067863 | | 19 | |  |
| ko04650 | Natural killer cell mediated cytotoxicity | 0.484894806 | | 0.998067863 | | 9 | |  |
| ko05210 | Colorectal cancer | 0.488843778 | | 0.998067863 | | 12 | |  |
| ko05131 | Shigellosis | 0.488843778 | | 0.998067863 | | 12 | |  |
| ko05416 | Viral myocarditis | 0.494822301 | | 0.998067863 | | 10 | |  |
| ko05140 | Leishmaniasis | 0.497231702 | | 0.998067863 | | 13 | |  |
| ko04142 | Lysosome | 0.500562846 | | 0.998067863 | | 19 | |  |
| ko05213 | Endometrial cancer | 0.502092169 | | 0.998067863 | | 8 | |  |
| ko05219 | Bladder cancer | 0.502092169 | | 0.998067863 | | 8 | |  |
| ko05215 | Prostate cancer | 0.504945879 | | 0.998067863 | | 14 | |  |
| ko05034 | Alcoholism | 0.512095161 | | 0.998067863 | | 15 | |  |
| ko04210 | Apoptosis | 0.513289728 | | 0.998067863 | | 24 | |  |
| ko04750 | Inflammatory mediator regulation of TRP channels | 0.525019627 | | 0.998067863 | | 17 | |  |
| ko04972 | Pancreatic secretion | 0.529248941 | | 0.998067863 | | 24 | |  |
| ko04064 | NF-kappa B signaling pathway | 0.534378732 | | 0.998067863 | | 12 | |  |
| ko04341 | Hedgehog signaling pathway - fly | 0.535387058 | | 0.998067863 | | 5 | |  |
| ko04622 | RIG-I-like receptor signaling pathway | 0.537698464 | | 0.998067863 | | 9 | |  |
| ko04024 | cAMP signaling pathway | 0.546859769 | | 0.998067863 | | 31 | |  |
| ko00830 | Retinol metabolism | 0.551002069 | | 0.998067863 | | 7 | |  |
| ko04614 | Renin-angiotensin system | 0.551778176 | | 0.998067863 | | 2 | |  |
| ko05221 | Acute myeloid leukemia | 0.557467046 | | 0.998067863 | | 8 | |  |
| ko03430 | Mismatch repair | 0.558558997 | | 0.998067863 | | 3 | |  |
| ko00532 | Glycosaminoglycan biosynthesis - CS/DS | 0.558558997 | | 0.998067863 | | 3 | |  |
| ko05016 | Huntington's disease | 0.562816991 | | 0.998067863 | | 35 | |  |
| ko00480 | Glutathione metabolism | 0.56333992 | | 0.998067863 | | 9 | |  |
| ko00260 | Glycine, serine and threonine metabolism | 0.56716198 | | 0.998067863 | | 18 | |  |
| ko04066 | HIF-1 signaling pathway | 0.567958465 | | 0.998067863 | | 22 | |  |
| ko00520 | Amino sugar and nucleotide sugar metabolism | 0.568748544 | | 0.998067863 | | 10 | |  |
| ko05032 | Morphine addiction | 0.578502775 | | 0.998067863 | | 12 | |  |
| ko05218 | Melanoma | 0.57970509 | | 0.998067863 | | 7 | |  |
| ko00600 | Sphingolipid metabolism | 0.57970509 | | 0.998067863 | | 7 | |  |
| ko04015 | Rap1 signaling pathway | 0.581565864 | | 0.998067863 | | 33 | |  |
| ko05323 | Rheumatoid arthritis | 0.588360831 | | 0.998067863 | | 9 | |  |
| ko05211 | Renal cell carcinoma | 0.588360831 | | 0.998067863 | | 9 | |  |
| ko04666 | Fc gamma R-mediated phagocytosis | 0.599894583 | | 0.998067863 | | 12 | |  |
| ko04960 | Aldosterone-regulated sodium reabsorption | 0.60185993 | | 0.998067863 | | 4 | |  |
| ko00900 | Terpenoid backbone biosynthesis | 0.602097147 | | 0.998067863 | | 3 | |  |
| ko03060 | Protein export | 0.602097147 | | 0.998067863 | | 3 | |  |
| ko04060 | Cytokine-cytokine receptor interaction | 0.603438358 | | 0.998067863 | | 13 | |  |
| ko05310 | Asthma | 0.606066313 | | 0.998067863 | | 2 | |  |
| ko00564 | Glycerophospholipid metabolism | 0.606856848 | | 0.998067863 | | 14 | |  |
| ko05133 | Pertussis | 0.606856848 | | 0.998067863 | | 14 | |  |
| ko04068 | FoxO signaling pathway | 0.609122469 | | 0.998067863 | | 20 | |  |
| ko04923 | Regulation of lipolysis in adipocytes | 0.618083157 | | 0.998067863 | | 11 | |  |
| ko05100 | Bacterial invasion of epithelial cells | 0.623444643 | | 0.998067863 | | 13 | |  |
| ko04640 | Hematopoietic cell lineage | 0.634800915 | | 0.998067863 | | 5 | |  |
| ko05412 | Arrhythmogenic right ventricular cardiomyopathy (ARVC) | 0.639348436 | | 0.998067863 | | 11 | |  |
| ko04924 | Renin secretion | 0.641103154 | | 0.998067863 | | 12 | |  |
| ko04977 | Vitamin digestion and absorption | 0.655197663 | | 0.998067863 | | 2 | |  |
| ko05214 | Glioma | 0.659962384 | | 0.998067863 | | 7 | |  |
| ko04520 | Adherens junction | 0.663094918 | | 0.998067863 | | 14 | |  |
| ko04912 | GnRH signaling pathway | 0.663094918 | | 0.998067863 | | 14 | |  |
| ko00350 | Tyrosine metabolism | 0.664861128 | | 0.998067863 | | 5 | |  |
| ko00980 | Metabolism of xenobiotics by cytochrome P450 | 0.670409832 | | 0.998067863 | | 4 | |  |
| ko03050 | Proteasome | 0.670409832 | | 0.998067863 | | 4 | |  |
| ko03420 | Nucleotide excision repair | 0.670409832 | | 0.998067863 | | 4 | |  |
| ko04010 | MAPK signaling pathway | 0.675642445 | | 0.998067863 | | 30 | |  |
| ko03020 | RNA polymerase | 0.680268107 | | 0.998067863 | | 3 | |  |
| ko00120 | Primary bile acid biosynthesis | 0.680268107 | | 0.998067863 | | 3 | |  |
| ko04145 | Phagosome | 0.690321168 | | 0.998067863 | | 23 | |  |
| ko04022 | cGMP-PKG signaling pathway | 0.692540059 | | 0.998067863 | | 33 | |  |
| ko04921 | Oxytocin signaling pathway | 0.694277294 | | 0.998067863 | | 26 | |  |
| ko00790 | Folate biosynthesis | 0.699294123 | | 0.998067863 | | 2 | |  |
| ko00534 | Glycosaminoglycan biosynthesis - HS/Hep | 0.699294123 | | 0.998067863 | | 2 | |  |
| ko04975 | Fat digestion and absorption | 0.70146682 | | 0.998067863 | | 4 | |  |
| ko04080 | Neuroactive ligand-receptor interaction | 0.703742599 | | 0.998067863 | | 23 | |  |
| ko04070 | Phosphatidylinositol signaling system | 0.715275224 | | 0.998067863 | | 13 | |  |
| ko04970 | Salivary secretion | 0.71764142 | | 0.998067863 | | 11 | |  |
| ko05152 | Tuberculosis | 0.718328397 | | 0.998067863 | | 25 | |  |
| ko05150 | Staphylococcus aureus infection | 0.719547481 | | 0.998067863 | | 10 | |  |
| ko04725 | Cholinergic synapse | 0.728628464 | | 0.998067863 | | 18 | |  |
| ko00140 | Steroid hormone biosynthesis | 0.730350257 | | 0.998067863 | | 4 | |  |
| ko04623 | Cytosolic DNA-sensing pathway | 0.730350257 | | 0.998067863 | | 4 | |  |
| ko04212 | Longevity regulating pathway - worm | 0.731839335 | | 0.998067863 | | 13 | |  |
| ko05231 | Choline metabolism in cancer | 0.735400233 | | 0.998067863 | | 11 | |  |
| ko00340 | Histidine metabolism | 0.736384211 | | 0.998067863 | | 6 | |  |
| ko04971 | Gastric acid secretion | 0.73799554 | | 0.998067863 | | 10 | |  |
| ko04350 | TGF-beta signaling pathway | 0.741329667 | | 0.998067863 | | 9 | |  |
| ko00780 | Biotin metabolism | 0.744893119 | | 0.998067863 | | 1 | |  |
| ko00051 | Fructose and mannose metabolism | 0.744946725 | | 0.998067863 | | 5 | |  |
| ko04330 | Notch signaling pathway | 0.744946725 | | 0.998067863 | | 5 | |  |
| ko03030 | DNA replication | 0.746401146 | | 0.998067863 | | 3 | |  |
| ko04810 | Regulation of actin cytoskeleton | 0.75495747 | | 0.998067863 | | 29 | |  |
| ko05204 | Chemical carcinogenesis | 0.757082834 | | 0.998067863 | | 4 | |  |
| ko00230 | Purine metabolism | 0.765273849 | | 0.998067863 | | 28 | |  |
| ko04726 | Serotonergic synapse | 0.768649483 | | 0.998067863 | | 11 | |  |
| ko04966 | Collecting duct acid secretion | 0.773418083 | | 0.998067863 | | 2 | |  |
| ko04713 | Circadian entrainment | 0.775245964 | | 0.998067863 | | 14 | |  |
| ko05020 | Prion diseases | 0.781714494 | | 0.998067863 | | 4 | |  |
| ko05132 | Salmonella infection | 0.784134358 | | 0.998067863 | | 11 | |  |
| ko04710 | Circadian rhythm | 0.789894598 | | 0.998067863 | | 5 | |  |
| ko04550 | Signaling pathways regulating pluripotency of stem cells | 0.799313098 | | 0.998067863 | | 15 | |  |
| ko04922 | Glucagon signaling pathway | 0.801973415 | | 0.998067863 | | 14 | |  |
| ko04120 | Ubiquitin mediated proteolysis | 0.801973415 | | 0.998067863 | | 14 | |  |
| ko04144 | Endocytosis | 0.804001886 | | 0.998067863 | | 30 | |  |
| ko04320 | Dorso-ventral axis formation | 0.804112239 | | 0.998067863 | | 2 | |  |
| ko00770 | Pantothenate and CoA biosynthesis | 0.804112239 | | 0.998067863 | | 2 | |  |
| ko04340 | Hedgehog signaling pathway | 0.809926735 | | 0.998067863 | | 5 | |  |
| ko00970 | Aminoacyl-tRNA biosynthesis | 0.817756667 | | 0.998067863 | | 10 | |  |
| ko02010 | ABC transporters | 0.82345092 | | 0.998067863 | | 7 | |  |
| ko00565 | Ether lipid metabolism | 0.824525288 | | 0.998067863 | | 3 | |  |
| ko04140 | Regulation of autophagy | 0.82734746 | | 0.998067863 | | 1 | |  |
| ko00920 | Sulfur metabolism | 0.82734746 | | 0.998067863 | | 1 | |  |
| ko00430 | Taurine and hypotaurine metabolism | 0.82734746 | | 0.998067863 | | 1 | |  |
| ko00750 | Vitamin B6 metabolism | 0.82734746 | | 0.998067863 | | 1 | |  |
| ko05230 | Central carbon metabolism in cancer | 0.830431425 | | 0.998067863 | | 8 | |  |
| ko00910 | Nitrogen metabolism | 0.831047584 | | 0.998067863 | | 2 | |  |
| ko04014 | Ras signaling pathway | 0.838450851 | | 0.998067863 | | 22 | |  |
| ko00240 | Pyrimidine metabolism | 0.841367344 | | 0.998067863 | | 13 | |  |
| ko04976 | Bile secretion | 0.844408171 | | 0.998067863 | | 8 | |  |
| ko04724 | Glutamatergic synapse | 0.848382742 | | 0.998067863 | | 14 | |  |
| ko04727 | GABAergic synapse | 0.850198314 | | 0.998067863 | | 9 | |  |
| ko05110 | Vibrio cholerae infection | 0.852840124 | | 0.998067863 | | 7 | |  |
| ko00601 | Glycosphingolipid biosynthesis - lacto and neolacto series | 0.854591844 | | 0.998067863 | | 2 | |  |
| ko03410 | Base excision repair | 0.854591844 | | 0.998067863 | | 2 | |  |
| ko00533 | Glycosaminoglycan biosynthesis - KS | 0.857966269 | | 0.998067863 | | 1 | |  |
| ko00670 | One carbon pool by folate | 0.860875371 | | 0.998067863 | | 4 | |  |
| ko04745 | Phototransduction - fly | 0.86433332 | | 0.998067863 | | 3 | |  |
| ko04072 | Phospholipase D signaling pathway | 0.8645811 | | 0.998067863 | | 15 | |  |
| ko05168 | Herpes simplex infection | 0.867994319 | | 0.998067863 | | 20 | |  |
| ko04130 | SNARE interactions in vesicular transport | 0.875100519 | | 0.998067863 | | 2 | |  |
| ko04215 | Apoptosis - multiple species | 0.881081203 | | 0.998067863 | | 3 | |  |
| ko04964 | Proximal tubule bicarbonate reclamation | 0.881081203 | | 0.998067863 | | 3 | |  |
| ko00604 | Glycosphingolipid biosynthesis - ganglio series | 0.883156265 | | 0.998067863 | | 1 | |  |
| ko00640 | Propanoate metabolism | 0.884064605 | | 0.998067863 | | 9 | |  |
| ko05031 | Amphetamine addiction | 0.888138262 | | 0.998067863 | | 6 | |  |
| ko03015 | mRNA surveillance pathway | 0.889357709 | | 0.998067863 | | 7 | |  |
| ko00562 | Inositol phosphate metabolism | 0.891358056 | | 0.998067863 | | 8 | |  |
| ko03022 | Basal transcription factors | 0.892909665 | | 0.998067863 | | 2 | |  |
| ko05130 | Pathogenic Escherichia coli infection | 0.899186577 | | 0.998067863 | | 6 | |  |
| ko00514 | Other types of O-glycan biosynthesis | 0.903879774 | | 0.998067863 | | 1 | |  |
| ko00450 | Selenocompound metabolism | 0.908331614 | | 0.998067863 | | 2 | |  |
| ko00330 | Arginine and proline metabolism | 0.911960088 | | 0.998067863 | | 15 | |  |
| ko00061 | Fatty acid biosynthesis | 0.920928578 | | 0.998067863 | | 1 | |  |
| ko00072 | Synthesis and degradation of ketone bodies | 0.920928578 | | 0.998067863 | | 1 | |  |
| ko04214 | Apoptosis - fly | 0.924167671 | | 0.998067863 | | 4 | |  |
| ko05033 | Nicotine addiction | 0.93495413 | | 0.998067863 | | 1 | |  |
| ko00360 | Phenylalanine metabolism | 0.93495413 | | 0.998067863 | | 1 | |  |
| ko00100 | Steroid biosynthesis | 0.93495413 | | 0.998067863 | | 1 | |  |
| ko04962 | Vasopressin-regulated water reabsorption | 0.936967583 | | 0.998067863 | | 5 | |  |
| ko03040 | Spliceosome | 0.939103152 | | 0.998067863 | | 9 | |  |
| ko04213 | Longevity regulating pathway - multiple species | 0.939103152 | | 0.998067863 | | 9 | |  |
| ko04720 | Long-term potentiation | 0.941388413 | | 0.998067863 | | 6 | |  |
| ko00310 | Lysine degradation | 0.944723281 | | 0.998067863 | | 9 | |  |
| ko00280 | Valine, leucine and isoleucine degradation | 0.947875537 | | 0.998067863 | | 14 | |  |
| ko00410 | beta-Alanine metabolism | 0.953271289 | | 0.998067863 | | 6 | |  |
| ko03440 | Homologous recombination | 0.955984431 | | 0.998067863 | | 1 | |  |
| ko00250 | Alanine, aspartate and glutamate metabolism | 0.956091647 | | 0.998067863 | | 5 | |  |
| ko04925 | Aldosterone synthesis and secretion | 0.958936314 | | 0.998067863 | | 9 | |  |
| ko00630 | Glyoxylate and dicarboxylate metabolism | 0.963754086 | | 0.998067863 | | 8 | |  |
| ko00590 | Arachidonic acid metabolism | 0.964957414 | | 0.998067863 | | 2 | |  |
| ko04270 | Vascular smooth muscle contraction | 0.965580707 | | 0.998067863 | | 12 | |  |
| ko05206 | MicroRNAs in cancer | 0.966361584 | | 0.998067863 | | 15 | |  |
| ko04020 | Calcium signaling pathway | 0.967947625 | | 0.998067863 | | 24 | |  |
| ko04961 | Endocrine and other factor-regulated calcium reabsorption | 0.97026483 | | 0.998067863 | | 2 | |  |
| ko04913 | Ovarian steroidogenesis | 0.970897489 | | 0.998067863 | | 3 | |  |
| ko00220 | Arginine biosynthesis | 0.970897489 | | 0.998067863 | | 3 | |  |
| ko04146 | Peroxisome | 0.97550173 | | 0.998067863 | | 9 | |  |
| ko03013 | RNA transport | 0.9762234 | | 0.998067863 | | 13 | |  |
| ko05134 | Legionellosis | 0.978565586 | | 0.998067863 | | 12 | |  |
| ko00270 | Cysteine and methionine metabolism | 0.978977093 | | 0.998067863 | | 8 | |  |
| ko04918 | Thyroid hormone synthesis | 0.982484572 | | 0.998067863 | | 7 | |  |
| ko05143 | African trypanosomiasis | 0.983423513 | | 0.998067863 | | 1 | |  |
| ko00380 | Tryptophan metabolism | 0.987631703 | | 0.998067863 | | 6 | |  |
| ko03460 | Fanconi anemia pathway | 0.989100017 | | 0.998067863 | | 2 | |  |
| ko00071 | Fatty acid degradation | 0.989106565 | | 0.998067863 | | 6 | |  |
| ko04740 | Olfactory transduction | 0.992244755 | | 0.998067863 | | 2 | |  |
| ko04742 | Taste transduction | 0.996527265 | | 0.998067863 | | 1 | |  |
| ko04911 | Insulin secretion | 0.997341048 | | 0.998067863 | | 5 | |  |
| ko04721 | Synaptic vesicle cycle | 0.997686041 | | 0.998067863 | | 2 | |  |
| ko00650 | Butanoate metabolism | 0.998067863 | | 0.998067863 | | 1 | |  |
|  |  | |  | |  | |  | |
|  |  | |  | |  | |  | |
|  |  | |  | |  | |  | |
| **Table S4C. KEGG pathway analysis of DEGs in the muscle between MS vs ML.** | | | | | | | | |
|  |  | |  | |  | |  | |
| Pathway ID | Description | P-Value | | Corrected P | | Unigene Count | |  |
| ko04974 | Protein digestion and absorption | 7.95E-05 | | 0.014142437 | | 10 | |  |
| ko04151 | PI3K-Akt signaling pathway | 0.001112065 | | 0.093546413 | | 15 | |  |
| ko04512 | ECM-receptor interaction | 0.001576625 | | 0.093546413 | | 7 | |  |
| ko04978 | Mineral absorption | 0.003907095 | | 0.173865735 | | 6 | |  |
| ko04510 | Focal adhesion | 0.014345335 | | 0.450288899 | | 10 | |  |
| ko05222 | Small cell lung cancer | 0.015178277 | | 0.450288899 | | 5 | |  |
| ko05205 | Proteoglycans in cancer | 0.018960512 | | 0.482138731 | | 10 | |  |
| ko04066 | HIF-1 signaling pathway | 0.022147497 | | 0.492781813 | | 7 | |  |
| ko04210 | Apoptosis | 0.028510809 | | 0.530755827 | | 7 | |  |
| ko05100 | Bacterial invasion of epithelial cells | 0.029817743 | | 0.530755827 | | 5 | |  |
| ko05164 | Influenza A | 0.034678441 | | 0.561160227 | | 7 | |  |
| ko04973 | Carbohydrate digestion and absorption | 0.045844156 | | 0.627712285 | | 3 | |  |
| ko00051 | Fructose and mannose metabolism | 0.045844156 | | 0.627712285 | | 3 | |  |
| ko05322 | Systemic lupus erythematosus | 0.058049293 | | 0.738055296 | | 4 | |  |
| ko05146 | Amoebiasis | 0.074069049 | | 0.878952712 | | 4 | |  |
| ko04060 | Cytokine-cytokine receptor interaction | 0.088325212 | | 0.929660465 | | 4 | |  |
| ko04145 | Phagosome | 0.090602164 | | 0.929660465 | | 6 | |  |
| ko04933 | AGE-RAGE signaling pathway in diabetic complications | 0.107846837 | | 0.929660465 | | 4 | |  |
| ko04540 | Gap junction | 0.111966723 | | 0.929660465 | | 4 | |  |
| ko05130 | Pathogenic Escherichia coli infection | 0.114378141 | | 0.929660465 | | 3 | |  |
| ko05416 | Viral myocarditis | 0.114378141 | | 0.929660465 | | 3 | |  |
| ko04260 | Cardiac muscle contraction | 0.114901855 | | 0.929660465 | | 5 | |  |
| ko04745 | Phototransduction - fly | 0.129937005 | | 0.932970962 | | 2 | |  |
| ko00010 | Glycolysis / Gluconeogenesis | 0.136811716 | | 0.932970962 | | 5 | |  |
| ko04612 | Antigen processing and presentation | 0.136825543 | | 0.932970962 | | 3 | |  |
| ko00524 | Butirosin and neomycin biosynthesis | 0.141870512 | | 0.932970962 | | 1 | |  |
| ko04640 | Hematopoietic cell lineage | 0.147242617 | | 0.932970962 | | 2 | |  |
| ko00510 | N-Glycan biosynthesis | 0.156063172 | | 0.932970962 | | 2 | |  |
| ko04910 | Insulin signaling pathway | 0.156368552 | | 0.932970962 | | 5 | |  |
| ko04150 | mTOR signaling pathway | 0.160626414 | | 0.932970962 | | 3 | |  |
| ko00603 | Glycosphingolipid biosynthesis - globo series | 0.163480596 | | 0.932970962 | | 1 | |  |
| ko04972 | Pancreatic secretion | 0.168624338 | | 0.932970962 | | 5 | |  |
| ko05150 | Staphylococcus aureus infection | 0.172966527 | | 0.932970962 | | 3 | |  |
| ko04142 | Lysosome | 0.191398002 | | 0.941625314 | | 4 | |  |
| ko04080 | Neuroactive ligand-receptor interaction | 0.198589213 | | 0.941625314 | | 5 | |  |
| ko04970 | Salivary secretion | 0.204885083 | | 0.941625314 | | 3 | |  |
| ko00920 | Sulfur metabolism | 0.205085439 | | 0.941625314 | | 1 | |  |
| ko00430 | Taurine and hypotaurine metabolism | 0.205085439 | | 0.941625314 | | 1 | |  |
| ko05218 | Melanoma | 0.21997249 | | 0.941625314 | | 2 | |  |
| ko05144 | Malaria | 0.21997249 | | 0.941625314 | | 2 | |  |
| ko05223 | Non-small cell lung cancer | 0.229302174 | | 0.941625314 | | 2 | |  |
| ko04062 | Chemokine signaling pathway | 0.239028113 | | 0.941625314 | | 4 | |  |
| ko05134 | Legionellosis | 0.244487546 | | 0.941625314 | | 4 | |  |
| ko00030 | Pentose phosphate pathway | 0.248031151 | | 0.941625314 | | 2 | |  |
| ko05214 | Glioma | 0.248031151 | | 0.941625314 | | 2 | |  |
| ko04261 | Adrenergic signaling in cardiomyocytes | 0.263158852 | | 0.941625314 | | 5 | |  |
| ko00514 | Other types of O-glycan biosynthesis | 0.263653515 | | 0.941625314 | | 1 | |  |
| ko04930 | Type II diabetes mellitus | 0.276195982 | | 0.941625314 | | 2 | |  |
| ko05162 | Measles | 0.278810621 | | 0.941625314 | | 3 | |  |
| ko00270 | Cysteine and methionine metabolism | 0.28568337 | | 0.941625314 | | 3 | |  |
| ko04360 | Axon guidance | 0.287319801 | | 0.941625314 | | 5 | |  |
| ko00360 | Phenylalanine metabolism | 0.300286686 | | 0.941625314 | | 1 | |  |
| ko00500 | Starch and sucrose metabolism | 0.304299573 | | 0.941625314 | | 2 | |  |
| ko04115 | p53 signaling pathway | 0.332198494 | | 0.941625314 | | 2 | |  |
| ko05202 | Transcriptional misregulation in cancer | 0.334034006 | | 0.941625314 | | 3 | |  |
| ko05220 | Chronic myeloid leukemia | 0.341430985 | | 0.941625314 | | 2 | |  |
| ko04922 | Glucagon signaling pathway | 0.347855418 | | 0.941625314 | | 3 | |  |
| ko00120 | Primary bile acid biosynthesis | 0.351855066 | | 0.941625314 | | 1 | |  |
| ko05145 | Toxoplasmosis | 0.361647877 | | 0.941625314 | | 3 | |  |
| ko05410 | Hypertrophic cardiomyopathy (HCM) | 0.375395379 | | 0.941625314 | | 3 | |  |
| ko05230 | Central carbon metabolism in cancer | 0.377919002 | | 0.941625314 | | 2 | |  |
| ko05212 | Pancreatic cancer | 0.377919002 | | 0.941625314 | | 2 | |  |
| ko04015 | Rap1 signaling pathway | 0.386668692 | | 0.941625314 | | 5 | |  |
| ko04960 | Aldosterone-regulated sodium reabsorption | 0.399631002 | | 0.941625314 | | 1 | |  |
| ko00450 | Selenocompound metabolism | 0.399631002 | | 0.941625314 | | 1 | |  |
| ko05131 | Shigellosis | 0.404726024 | | 0.941625314 | | 2 | |  |
| ko04630 | Jak-STAT signaling pathway | 0.404726024 | | 0.941625314 | | 2 | |  |
| ko00982 | Drug metabolism - cytochrome P450 | 0.414762253 | | 0.941625314 | | 1 | |  |
| ko04660 | T cell receptor signaling pathway | 0.439578719 | | 0.941625314 | | 2 | |  |
| ko04975 | Fat digestion and absorption | 0.443892802 | | 0.941625314 | | 1 | |  |
| ko04666 | Fc gamma R-mediated phagocytosis | 0.448117608 | | 0.941625314 | | 2 | |  |
| ko04915 | Estrogen signaling pathway | 0.449571923 | | 0.941625314 | | 3 | |  |
| ko05414 | Dilated cardiomyopathy (DCM) | 0.449571923 | | 0.941625314 | | 3 | |  |
| ko05200 | Pathways in cancer | 0.453906359 | | 0.941625314 | | 7 | |  |
| ko04530 | Tight junction | 0.461021337 | | 0.941625314 | | 5 | |  |
| ko04068 | FoxO signaling pathway | 0.462697722 | | 0.941625314 | | 3 | |  |
| ko04924 | Renin secretion | 0.464972797 | | 0.941625314 | | 2 | |  |
| ko04964 | Proximal tubule bicarbonate reclamation | 0.471576535 | | 0.941625314 | | 1 | |  |
| ko04726 | Serotonergic synapse | 0.481520461 | | 0.941625314 | | 2 | |  |
| ko00052 | Galactose metabolism | 0.484898393 | | 0.941625314 | | 1 | |  |
| ko05020 | Prion diseases | 0.484898393 | | 0.941625314 | | 1 | |  |
| ko03013 | RNA transport | 0.488528057 | | 0.941625314 | | 3 | |  |
| ko05132 | Salmonella infection | 0.48967542 | | 0.941625314 | | 2 | |  |
| ko05203 | Viral carcinogenesis | 0.497720532 | | 0.941625314 | | 4 | |  |
| ko00350 | Tyrosine metabolism | 0.497885154 | | 0.941625314 | | 1 | |  |
| ko04110 | Cell cycle | 0.505741525 | | 0.941625314 | | 2 | |  |
| ko03320 | PPAR signaling pathway | 0.505741525 | | 0.941625314 | | 2 | |  |
| ko04213 | Longevity regulating pathway - multiple species | 0.521475728 | | 0.941625314 | | 2 | |  |
| ko05034 | Alcoholism | 0.529216193 | | 0.941625314 | | 2 | |  |
| ko05133 | Pertussis | 0.529216193 | | 0.941625314 | | 2 | |  |
| ko04070 | Phosphatidylinositol signaling system | 0.536871265 | | 0.941625314 | | 2 | |  |
| ko04810 | Regulation of actin cytoskeleton | 0.55534752 | | 0.941625314 | | 4 | |  |
| ko04380 | Osteoclast differentiation | 0.566626581 | | 0.941625314 | | 2 | |  |
| ko00830 | Retinol metabolism | 0.569225187 | | 0.941625314 | | 1 | |  |
| ko05030 | Cocaine addiction | 0.569225187 | | 0.941625314 | | 1 | |  |
| ko00340 | Histidine metabolism | 0.590682016 | | 0.941625314 | | 1 | |  |
| ko05213 | Endometrial cancer | 0.601007154 | | 0.941625314 | | 1 | |  |
| ko05219 | Bladder cancer | 0.601007154 | | 0.941625314 | | 1 | |  |
| ko04750 | Inflammatory mediator regulation of TRP channels | 0.601849108 | | 0.941625314 | | 2 | |  |
| ko04664 | Fc epsilon RI signaling pathway | 0.611072424 | | 0.941625314 | | 1 | |  |
| ko05221 | Acute myeloid leukemia | 0.62088435 | | 0.941625314 | | 1 | |  |
| ko04014 | Ras signaling pathway | 0.628885721 | | 0.941625314 | | 3 | |  |
| ko00620 | Pyruvate metabolism | 0.634870467 | | 0.941625314 | | 2 | |  |
| ko05142 | Chagas disease (American trypanosomiasis) | 0.634870467 | | 0.941625314 | | 2 | |  |
| ko04650 | Natural killer cell mediated cytotoxicity | 0.639773463 | | 0.941625314 | | 1 | |  |
| ko04728 | Dopaminergic synapse | 0.64121195 | | 0.941625314 | | 2 | |  |
| ko04919 | Thyroid hormone signaling pathway | 0.647466457 | | 0.941625314 | | 2 | |  |
| ko00260 | Glycine, serine and threonine metabolism | 0.647466457 | | 0.941625314 | | 2 | |  |
| ko04931 | Insulin resistance | 0.647466457 | | 0.941625314 | | 2 | |  |
| ko04611 | Platelet activation | 0.649526529 | | 0.941625314 | | 3 | |  |
| ko05152 | Tuberculosis | 0.649526529 | | 0.941625314 | | 3 | |  |
| ko04921 | Oxytocin signaling pathway | 0.664463464 | | 0.941625314 | | 3 | |  |
| ko04370 | VEGF signaling pathway | 0.666361019 | | 0.941625314 | | 1 | |  |
| ko05031 | Amphetamine addiction | 0.674781052 | | 0.941625314 | | 1 | |  |
| ko05211 | Renal cell carcinoma | 0.674781052 | | 0.941625314 | | 1 | |  |
| ko04662 | B cell receptor signaling pathway | 0.674781052 | | 0.941625314 | | 1 | |  |
| ko05161 | Hepatitis B | 0.677447876 | | 0.941625314 | | 2 | |  |
| ko02010 | ABC transporters | 0.682989067 | | 0.941625314 | | 1 | |  |
| ko04270 | Vascular smooth muscle contraction | 0.683189115 | | 0.941625314 | | 2 | |  |
| ko04072 | Phospholipase D signaling pathway | 0.683189115 | | 0.941625314 | | 2 | |  |
| ko04730 | Long-term depression | 0.69099039 | | 0.941625314 | | 1 | |  |
| ko05110 | Vibrio cholerae infection | 0.698790214 | | 0.941625314 | | 1 | |  |
| ko04152 | AMPK signaling pathway | 0.70532178 | | 0.941625314 | | 2 | |  |
| ko04725 | Cholinergic synapse | 0.70532178 | | 0.941625314 | | 2 | |  |
| ko00520 | Amino sugar and nucleotide sugar metabolism | 0.706393601 | | 0.941625314 | | 1 | |  |
| ko04670 | Leukocyte transendothelial migration | 0.715897294 | | 0.941625314 | | 2 | |  |
| ko04720 | Long-term potentiation | 0.72103069 | | 0.941625314 | | 1 | |  |
| ko04012 | ErbB signaling pathway | 0.728073894 | | 0.941625314 | | 1 | |  |
| ko04920 | Adipocytokine signaling pathway | 0.728073894 | | 0.941625314 | | 1 | |  |
| ko04620 | Toll-like receptor signaling pathway | 0.734939676 | | 0.941625314 | | 1 | |  |
| ko04917 | Prolactin signaling pathway | 0.734939676 | | 0.941625314 | | 1 | |  |
| ko04390 | Hippo signaling pathway | 0.736093349 | | 0.941625314 | | 2 | |  |
| ko04010 | MAPK signaling pathway | 0.74434576 | | 0.941625314 | | 3 | |  |
| ko05210 | Colorectal cancer | 0.7481567 | | 0.941625314 | | 1 | |  |
| ko04141 | Protein processing in endoplasmic reticulum | 0.75042747 | | 0.941625314 | | 2 | |  |
| ko04923 | Regulation of lipolysis in adipocytes | 0.754516527 | | 0.941625314 | | 1 | |  |
| ko04971 | Gastric acid secretion | 0.76071611 | | 0.941625314 | | 1 | |  |
| ko04064 | NF-kappa B signaling pathway | 0.76071611 | | 0.941625314 | | 1 | |  |
| ko05412 | Arrhythmogenic right ventricular cardiomyopathy (ARVC) | 0.76071611 | | 0.941625314 | | 1 | |  |
| ko00020 | Citrate cycle (TCA cycle) | 0.772650554 | | 0.941625314 | | 1 | |  |
| ko05206 | MicroRNAs in cancer | 0.772830249 | | 0.941625314 | | 2 | |  |
| ko05140 | Leishmaniasis | 0.778393171 | | 0.941625314 | | 1 | |  |
| ko04391 | Hippo signaling pathway - fly | 0.783991062 | | 0.941625314 | | 1 | |  |
| ko04914 | Progesterone-mediated oocyte maturation | 0.783991062 | | 0.941625314 | | 1 | |  |
| ko05169 | Epstein-Barr virus infection | 0.785414445 | | 0.941625314 | | 2 | |  |
| ko04144 | Endocytosis | 0.788667144 | | 0.941625314 | | 3 | |  |
| ko00640 | Propanoate metabolism | 0.789447866 | | 0.941625314 | | 1 | |  |
| ko05231 | Choline metabolism in cancer | 0.789447866 | | 0.941625314 | | 1 | |  |
| ko00380 | Tryptophan metabolism | 0.799952312 | | 0.941625314 | | 1 | |  |
| ko05215 | Prostate cancer | 0.805006785 | | 0.941625314 | | 1 | |  |
| ko04668 | TNF signaling pathway | 0.814736674 | | 0.941625314 | | 1 | |  |
| ko04918 | Thyroid hormone synthesis | 0.819418419 | | 0.941625314 | | 1 | |  |
| ko04932 | Non-alcoholic fatty liver disease (NAFLD) | 0.823036461 | | 0.941625314 | | 2 | |  |
| ko03040 | Spliceosome | 0.823982118 | | 0.941625314 | | 1 | |  |
| ko00564 | Glycerophospholipid metabolism | 0.828430742 | | 0.941625314 | | 1 | |  |
| ko04723 | Retrograde endocannabinoid signaling | 0.832767184 | | 0.941625314 | | 1 | |  |
| ko04925 | Aldosterone synthesis and secretion | 0.841114747 | | 0.941625314 | | 1 | |  |
| ko04520 | Adherens junction | 0.841114747 | | 0.941625314 | | 1 | |  |
| ko04912 | GnRH signaling pathway | 0.841114747 | | 0.941625314 | | 1 | |  |
| ko04114 | Oocyte meiosis | 0.860209606 | | 0.941910786 | | 1 | |  |
| ko05010 | Alzheimer's disease | 0.860682691 | | 0.941910786 | | 3 | |  |
| ko04713 | Circadian entrainment | 0.867190167 | | 0.941910786 | | 1 | |  |
| ko05160 | Hepatitis C | 0.870548915 | | 0.941910786 | | 1 | |  |
| ko04722 | Neurotrophin signaling pathway | 0.873822911 | | 0.941910786 | | 1 | |  |
| ko04724 | Glutamatergic synapse | 0.886113257 | | 0.941910786 | | 1 | |  |
| ko04211 | Longevity regulating pathway | 0.886113257 | | 0.941910786 | | 1 | |  |
| ko04071 | Sphingolipid signaling pathway | 0.888994449 | | 0.941910786 | | 1 | |  |
| ko04550 | Signaling pathways regulating pluripotency of stem cells | 0.888994449 | | 0.941910786 | | 1 | |  |
| ko00330 | Arginine and proline metabolism | 0.918388434 | | 0.959651977 | | 1 | |  |
| ko04020 | Calcium signaling pathway | 0.919340232 | | 0.959651977 | | 2 | |  |
| ko05016 | Huntington's disease | 0.925773141 | | 0.959651977 | | 2 | |  |
| ko04022 | cGMP-PKG signaling pathway | 0.927304157 | | 0.959651977 | | 2 | |  |
| ko00190 | Oxidative phosphorylation | 0.951147579 | | 0.968712481 | | 1 | |  |
| ko03010 | Ribosome | 0.952107041 | | 0.968712481 | | 3 | |  |
| ko05168 | Herpes simplex infection | 0.952385866 | | 0.968712481 | | 1 | |  |
| ko05012 | Parkinson's disease | 0.966767442 | | 0.977753436 | | 1 | |  |
| ko04024 | cAMP signaling pathway | 0.976208007 | | 0.977974654 | | 1 | |  |
| ko00230 | Purine metabolism | 0.977974654 | | 0.977974654 | | 1 | |  |

| **TABLE S4 KEGG pathway analysis of DEGs in this study.** | | | | |  | |  | |
| --- | --- | --- | --- | --- | --- | --- | --- | --- |
|  |  | |  | |  | |  | |
| **Table S4D. Top 20 KEGG pathway of upregulated DEGs in the muscle between MS vs MC.** | | | | | | | | |
|  |  | |  | |  | |  | |
| Pathway ID | Description | P-Value | | Corrected P | | Unigene Count | |  |
| ko04530 | Tight junction | 4.79E-05 | | 0.010397616 | | 26 | |  |
| ko05010 | Alzheimer's disease | 0.0008011 | | 0.086917064 | | 24 | |  |
| ko04022 | cGMP-PKG signaling pathway | 0.001936 | | 0.140037449 | | 21 | |  |
| ko04260 | Cardiac muscle contraction | 0.0086856 | | 0.364224382 | | 13 | |  |
| ko04261 | Adrenergic signaling in cardiomyocytes | 0.0090371 | | 0.364224382 | | 16 | |  |
| ko04932 | Non-alcoholic fatty liver disease (NAFLD) | 0.0100707 | | 0.364224382 | | 15 | |  |
| ko04910 | Insulin signaling pathway | 0.0183524 | | 0.505924834 | | 13 | |  |
| ko04931 | Insulin resistance | 0.0186516 | | 0.505924834 | | 11 | |  |
| ko04020 | Calcium signaling pathway | 0.022741 | | 0.548311171 | | 17 | |  |
| ko05220 | Chronic myeloid leukemia | 0.027917 | | 0.605799016 | | 7 | |  |
| ko05212 | Pancreatic cancer | 0.0395643 | | 0.780495901 | | 7 | |  |
| ko04972 | Pancreatic secretion | 0.0437277 | | 0.79074271 | | 12 | |  |
| ko04013 | MAPK signaling pathway - fly | 0.0515981 | | 0.861291335 | | 8 | |  |
| ko05410 | Hypertrophic cardiomyopathy (HCM) | 0.0771474 | | 0.950975613 | | 9 | |  |
| ko04211 | Longevity regulating pathway | 0.0771474 | | 0.950975613 | | 9 | |  |
| ko04810 | Regulation of actin cytoskeleton | 0.0841799 | | 0.950975613 | | 14 | |  |
| ko04920 | Adipocytokine signaling pathway | 0.0929373 | | 0.950975613 | | 6 | |  |
| ko04930 | Type II diabetes mellitus | 0.0988594 | | 0.950975613 | | 5 | |  |
| ko00190 | Oxidative phosphorylation | 0.1054048 | | 0.950975613 | | 11 | |  |
| ko05012 | Parkinson's disease | 0.1111247 | | 0.950975613 | | 12 | |  |
|  |  | |  | |  | |  | |
|  |  | |  | |  | |  | |
|  |  | |  | |  | |  | |
| **Table S4E. Top 20 KEGG pathway of upregulated DEGs in the muscle between ML vs MC.** | | | | | | | | |
|  |  | |  | |  | |  | |
| Pathway ID | Description | P-Value | | Corrected P | | Unigene Count | |  |
| ko04512 | ECM-receptor interaction | 3.26E-03 | | 0.733927886 | | 21 | |  |
| ko04974 | Protein digestion and absorption | 0.0064145 | | 0.733927886 | | 24 | |  |
| ko04151 | PI3K-Akt signaling pathway | 0.0083496 | | 0.733927886 | | 53 | |  |
| ko04933 | AGE-RAGE signaling pathway in diabetic complications | 0.0158729 | | 0.733927886 | | 20 | |  |
| ko05142 | Chagas disease (American trypanosomiasis) | 0.0176137 | | 0.733927886 | | 23 | |  |
| ko05164 | Influenza A | 0.0228277 | | 0.733927886 | | 29 | |  |
| ko05222 | Small cell lung cancer | 0.0242443 | | 0.733927886 | | 16 | |  |
| ko04390 | Hippo signaling pathway | 0.0247474 | | 0.733927886 | | 26 | |  |
| ko05220 | Chronic myeloid leukemia | 0.0320288 | | 0.733927886 | | 14 | |  |
| ko04530 | Tight junction | 0.0347805 | | 0.733927886 | | 39 | |  |
| ko04722 | Neurotrophin signaling pathway | 0.0348143 | | 0.733927886 | | 21 | |  |
| ko05223 | Non-small cell lung cancer | 0.0395932 | | 0.733927886 | | 11 | |  |
| ko00510 | N-Glycan biosynthesis | 0.0445977 | | 0.733927886 | | 9 | |  |
| ko04514 | Cell adhesion molecules (CAMs) | 0.0454062 | | 0.733927886 | | 18 | |  |
| ko05146 | Amoebiasis | 0.0489554 | | 0.733927886 | | 16 | |  |
| ko04391 | Hippo signaling pathway - fly | 0.0489554 | | 0.733927886 | | 16 | |  |
| ko05212 | Pancreatic cancer | 0.0523381 | | 0.733927886 | | 14 | |  |
| ko04013 | MAPK signaling pathway - fly | 0.0548907 | | 0.733927886 | | 17 | |  |
| ko04071 | Sphingolipid signaling pathway | 0.0549759 | | 0.733927886 | | 21 | |  |
| ko04931 | Insulin resistance | 0.0549759 | | 0.733927886 | | 21 | |  |
|  |  | |  | |  | |  | |
|  |  | |  | |  | |  | |
|  |  | |  | |  | |  | |
| **Table S4F. Top 20 KEGG pathway of upregulated DEGs in the muscle between MS vs ML.** | | | | | | | | |
|  |  | |  | |  | |  | |
| Pathway ID | Description | P-Value | | Corrected P | | Unigene Count | |  |
| ko04260 | Cardiac muscle contraction | 2.44E-04 | | 0.008798326 | | 4 | |  |
| ko04261 | Adrenergic signaling in cardiomyocytes | 0.0079037 | | 0.102380144 | | 3 | |  |
| ko05416 | Viral myocarditis | 0.0085317 | | 0.102380144 | | 2 | |  |
| ko04150 | mTOR signaling pathway | 0.011616 | | 0.104544381 | | 2 | |  |
| ko04530 | Tight junction | 0.0159077 | | 0.114535395 | | 3 | |  |
| ko03010 | Ribosome | 0.0394565 | | 0.196790898 | | 3 | |  |
| ko04066 | HIF-1 signaling pathway | 0.040904 | | 0.196790898 | | 2 | |  |
| ko04910 | Insulin signaling pathway | 0.0437313 | | 0.196790898 | | 2 | |  |
| ko04973 | Carbohydrate digestion and absorption | 0.0869635 | | 0.347854142 | | 1 | |  |
| ko05205 | Proteoglycans in cancer | 0.1001542 | | 0.351065938 | | 2 | |  |
| ko00500 | Starch and sucrose metabolism | 0.1223034 | | 0.351065938 | | 1 | |  |
| ko04115 | p53 signaling pathway | 0.1302664 | | 0.351065938 | | 1 | |  |
| ko04151 | PI3K-Akt signaling pathway | 0.149257 | | 0.351065938 | | 2 | |  |
| ko04978 | Mineral absorption | 0.1537326 | | 0.351065938 | | 1 | |  |
| ko00640 | Propanoate metabolism | 0.1690307 | | 0.351065938 | | 1 | |  |
| ko04110 | Cell cycle | 0.1815717 | | 0.351065938 | | 1 | |  |
| ko00270 | Cysteine and methionine metabolism | 0.1963762 | | 0.351065938 | | 1 | |  |
| ko04974 | Protein digestion and absorption | 0.2157077 | | 0.351065938 | | 1 | |  |
| ko04922 | Glucagon signaling pathway | 0.2180918 | | 0.351065938 | | 1 | |  |
| ko00620 | Pyruvate metabolism | 0.2252017 | | 0.351065938 | | 1 | |  |

| **TABLE S4 KEGG pathway analysis of DEGs in this study.** | | | | |  | |  | |
| --- | --- | --- | --- | --- | --- | --- | --- | --- |
|  |  | |  | |  | |  | |
| **Table S4G. Top 20 KEGG pathway of downregulated DEGs in the muscle between MS vs MC.** | | | | | | | | |
|  |  | |  | |  | |  | |
| Pathway ID | Description | P-Value | | Corrected P | | Unigene Count | |  |
| ko03010 | Ribosome | 3.97E-09 | | 0.000000651 | | 27 | |  |
| ko00620 | Pyruvate metabolism | 0.0000365 | | 0.002991273 | | 11 | |  |
| ko00260 | Glycine, serine and threonine metabolism | 0.0002005 | | 0.010961812 | | 10 | |  |
| ko00020 | Citrate cycle (TCA cycle) | 0.0002965 | | 0.012156779 | | 8 | |  |
| ko00860 | Porphyrin and chlorophyll metabolism | 0.0005062 | | 0.016602781 | | 6 | |  |
| ko00630 | Glyoxylate and dicarboxylate metabolism | 0.0007205 | | 0.018972664 | | 8 | |  |
| ko00010 | Glycolysis / Gluconeogenesis | 0.0008098 | | 0.018972664 | | 10 | |  |
| ko00280 | Valine, leucine and isoleucine degradation | 0.0020809 | | 0.042659139 | | 9 | |  |
| ko00040 | Pentose and glucuronate interconversions | 0.0030501 | | 0.055579381 | | 5 | |  |
| ko00340 | Histidine metabolism | 0.0034242 | | 0.056156745 | | 5 | |  |
| ko00053 | Ascorbate and aldarate metabolism | 0.0130779 | | 0.194978994 | | 4 | |  |
| ko00190 | Oxidative phosphorylation | 0.0177173 | | 0.226859299 | | 8 | |  |
| ko00030 | Pentose phosphate pathway | 0.0206039 | | 0.226859299 | | 4 | |  |
| ko04146 | Peroxisome | 0.0211357 | | 0.226859299 | | 6 | |  |
| ko00130 | Ubiquinone and other terpenoid-quinone biosynthesis | 0.0220777 | | 0.226859299 | | 2 | |  |
| ko00561 | Glycerolipid metabolism | 0.0221326 | | 0.226859299 | | 5 | |  |
| ko00640 | Propanoate metabolism | 0.0282411 | | 0.238582367 | | 5 | |  |
| ko00970 | Aminoacyl-tRNA biosynthesis | 0.0282411 | | 0.238582367 | | 5 | |  |
| ko00380 | Tryptophan metabolism | 0.0316583 | | 0.238582367 | | 5 | |  |
| ko05012 | Parkinson's disease | 0.0321715 | | 0.238582367 | | 8 | |  |
|  |  | |  | |  | |  | |
|  |  | |  | |  | |  | |
|  |  | |  | |  | |  | |
| **Table S4H. Top 20 KEGG pathway of downregulated DEGs in the muscle between ML vs MC.** | | | | | | | | |
|  |  | |  | |  | |  | |
| Pathway ID | Description | P-Value | | Corrected P | | Unigene Count | |  |
| ko03010 | Ribosome | 8.84E-22 | | 1.91E-19 | | 64 | |  |
| ko00190 | Oxidative phosphorylation | 2.88E-06 | | 0.000311188 | | 22 | |  |
| ko05012 | Parkinson's disease | 0.0000157 | | 0.00112851 | | 22 | |  |
| ko05016 | Huntington's disease | 0.0000478 | | 0.002580125 | | 24 | |  |
| ko00620 | Pyruvate metabolism | 0.0001569 | | 0.006778986 | | 15 | |  |
| ko00020 | Citrate cycle (TCA cycle) | 0.0002069 | | 0.007448052 | | 12 | |  |
| ko04932 | Non-alcoholic fatty liver disease (NAFLD) | 0.0003485 | | 0.01075432 | | 18 | |  |
| ko00010 | Glycolysis / Gluconeogenesis | 0.0004409 | | 0.01190532 | | 16 | |  |
| ko04260 | Cardiac muscle contraction | 0.0006859 | | 0.01646054 | | 15 | |  |
| ko00860 | Porphyrin and chlorophyll metabolism | 0.0010721 | | 0.021052936 | | 8 | |  |
| ko00040 | Pentose and glucuronate interconversions | 0.0010721 | | 0.021052936 | | 8 | |  |
| ko05010 | Alzheimer's disease | 0.0013369 | | 0.024064455 | | 22 | |  |
| ko00130 | Ubiquinone and other terpenoid-quinone biosynthesis | 0.0017872 | | 0.029694695 | | 4 | |  |
| ko00260 | Glycine, serine and threonine metabolism | 0.0044325 | | 0.068386463 | | 12 | |  |
| ko03008 | Ribosome biogenesis in eukaryotes | 0.0062881 | | 0.09054854 | | 7 | |  |
| ko00970 | Aminoacyl-tRNA biosynthesis | 0.0092044 | | 0.124259983 | | 9 | |  |
| ko03018 | RNA degradation | 0.0100893 | | 0.128193812 | | 9 | |  |
| ko00480 | Glutathione metabolism | 0.0128176 | | 0.153811651 | | 7 | |  |
| ko00330 | Arginine and proline metabolism | 0.0247902 | | 0.281825445 | | 11 | |  |
| ko04150 | mTOR signaling pathway | 0.0326748 | | 0.352887315 | | 7 | |  |
|  |  | |  | |  | |  | |
|  |  | |  | |  | |  | |
|  |  | |  | |  | |  | |
| **Table S4I. Top 20 KEGG pathway of downregulated DEGs in the muscle between MS vs ML.** | | | | | | | | |
|  |  | |  | |  | |  | |
| Pathway ID | Description | P-Value | | Corrected P | | Unigene Count | |  |
| ko04974 | Protein digestion and absorption | 1.49E-04 | | 0.025962943 | | 9 | |  |
| ko04512 | ECM-receptor interaction | 0.0007779 | | 0.067677039 | | 7 | |  |
| ko04151 | PI3K-Akt signaling pathway | 0.0026225 | | 0.152105613 | | 13 | |  |
| ko04510 | Focal adhesion | 0.0064362 | | 0.279975129 | | 10 | |  |
| ko05222 | Small cell lung cancer | 0.009325 | | 0.290204154 | | 5 | |  |
| ko04978 | Mineral absorption | 0.010007 | | 0.290204154 | | 5 | |  |
| ko05100 | Bacterial invasion of epithelial cells | 0.0187821 | | 0.466868109 | | 5 | |  |
| ko00051 | Fructose and mannose metabolism | 0.0337836 | | 0.711800646 | | 3 | |  |
| ko05322 | Systemic lupus erythematosus | 0.040153 | | 0.711800646 | | 4 | |  |
| ko04210 | Apoptosis | 0.0453674 | | 0.711800646 | | 6 | |  |
| ko05146 | Amoebiasis | 0.0517777 | | 0.711800646 | | 4 | |  |
| ko05205 | Proteoglycans in cancer | 0.0528452 | | 0.711800646 | | 8 | |  |
| ko05164 | Influenza A | 0.0536965 | | 0.711800646 | | 6 | |  |
| ko04145 | Phagosome | 0.0572713 | | 0.711800646 | | 6 | |  |
| ko04060 | Cytokine-cytokine receptor interaction | 0.0622612 | | 0.722229691 | | 4 | |  |
| ko04933 | AGE-RAGE signaling pathway in diabetic complications | 0.0768106 | | 0.817885395 | | 4 | |  |
| ko04540 | Gap junction | 0.0799083 | | 0.817885395 | | 4 | |  |
| ko05130 | Pathogenic Escherichia coli infection | 0.0868517 | | 0.839565983 | | 3 | |  |
| ko04066 | HIF-1 signaling pathway | 0.0965854 | | 0.880237938 | | 5 | |  |
| ko04612 | Antigen processing and presentation | 0.1047042 | | 0.880237938 | | 3 | |  |
